# Optical detection of di- and triphosphate anions with mixed monolayer-protected gold nanoparticles containing zinc(II)–dipicolylamine complexes

**DOI:** 10.3762/bjoc.16.219

**Published:** 2020-11-02

**Authors:** Lena Reinke, Julia Bartl, Marcus Koch, Stefan Kubik

**Affiliations:** 1 Fachbereich Chemie - Organische Chemie, Technische Universität Kaiserslautern, Erwin-Schrödinger-Straße, 67663 Kaiserslautern, Germany, Fax: +49-631-205-3921; 2 INM – Leibniz Institute for New Materials, Campus D2 2, 66123 Saarbrücken, Germany

**Keywords:** chemosensor, diphosphate, gold nanoparticles, optical sensing, triphosphate

## Abstract

Gold nanoparticles covered with a mixture of ligands of which one type contains solubilizing triethylene glycol residues and the other peripheral zinc(II)–dipicolylamine (DPA) complexes allowed the optical detection of hydrogenphosphate, diphosphate, and triphosphate anions in water/methanol 1:2 (v/v). These anions caused the bright red solutions of the nanoparticles to change their color because of nanoparticle aggregation followed by precipitation, whereas halides or oxoanions such as sulfate, nitrate, or carbonate produced no effect. The sensitivity of phosphate sensing depended on the nature of the anion, with diphosphate and triphosphate inducing visual changes at significantly lower concentrations than hydrogenphosphate. In addition, the sensing sensitivity was also affected by the ratio of the ligands on the nanoparticle surface, decreasing as the number of immobilized zinc(II)–dipicolylamine groups increased. A nanoparticle containing a 9:1 ratio of the solubilizing and the anion-binding ligand showed a color change at diphosphate and triphosphate concentrations as low as 10 μmol/L, for example, and precipitated at slightly higher concentrations. Hydrogenphosphate induced a nanoparticle precipitation only at a concentration of ca. 400 μmol/L, at which the precipitates formed in the presence of diphosphates and triphosphates redissolved. A nanoparticle containing fewer binding sites was more sensitive, while increasing the relative number of zinc(II)–dipicolylamine complexes beyond 25% had a negative impact on the limit of detection and the optical response. Transmission electron microscopy provided evidence that the changes of the nanoparticle properties observed in the presence of the phosphates were due to a nanoparticle crosslinking, consistent with the preferred binding mode of zinc(II)–dipicolylamine complexes with phosphate anions which involves binding of the anion between two metal centers. This work thus provided information on how the behavior of mixed monolayer-protected gold nanoparticles is affected by multivalent interactions, at the same time introducing a method to assess whether certain biologically relevant anions are present in an aqueous solution within a specific concentration range.

## Introduction

Gold nanoparticles (AuNPs) are versatile platforms for the development of optical probes [1–6]. They are accessible in different sizes and shapes, can be stabilized by immobilizing suitable ligands, and can easily be functionalized with ligands containing binding sites that mediate the interaction with the analyte. Different strategies allow analyte sensing [1–6]. The analyte can be detected by relying on the enhancement of the Raman scattering intensity, if it is bound close to the gold surface (surface enhanced Raman scattering, SERS), for example, or by the release from the metal surface of a chromophore the fluorescence of which is quenched in the absence of the analyte by Förster resonance energy transfer (FRET). In the latter case, the analyte binding either causes the chromophore to dissociate from the nanoparticle, if it is bound noncovalently (indicator displacement), or to move away from the metal surface as a consequence of a conformational reorganization of the linker connecting the chromophore with the surface. The likely most frequently used strategy of analyte detection relies on the color change of AuNP solutions resulting from analyte-induced nanoparticle crosslinking. Depending on whether soluble or insoluble aggregates are formed, the solutions either lose their color because of nanoparticle precipitation or they adopt a different color. Dispersed spherical AuNPs with a diameter of ca. 10 nm lead to intensely colored red solutions, for example, which turn purple or blue upon nanoparticle aggregation because the localized surface plasmon resonance of the individual AuNPs starts to couple when they approach each other [7–9].

Early examples of optical probes working in this way are the nanoparticles introduced by Mirkin et al., containing immobilized oligonucleotides that aggregated in the presence of single-stranded DNA with a complementary base sequence [10], the AuNPs with peripheral carboxylate groups introduced by the Hupp group [11] that responded to divalent transition metal ions, or Chen at al.’s crown ether-decorated AuNPs that allowed the sensing of potassium ions [12]. Numerous other such probes have been described for analytes ranging from inorganic ions over low-molecular-weight neutral and charged organic compounds, such as carboxylic acids, amino acids, and nucleotides, to larger biomolecules such as peptides and proteins [1–6,13]. All of these systems have specific areas of application. AuNP-based probes for inorganic anions, for example, can serve to monitor the water quality [14–15]. An example is the probe for halides that was described by the Sessler group. It comprised AuNPs with immobilized calix[4]pyrrole residues that are known to interact with chloride and fluoride. The binding of these anions to the immobilized receptor units strengthened their interaction with a simultaneously present bis(imidazolium) ion, which in turn caused nanoparticle crosslinking [16–17]. Our group recently showed that a mixed monolayer-protected AuNP containing solubilizing triethylene glycol residues and cyclopeptides selectively precipitated from water in the presence of sulfate [18]. The anion sensing in this case relied on the sulfate affinity of the cyclopeptide and on the propensity of this receptor to bind a sulfate ion in the form of a sandwich-type 2:1 complexes, rendering the presence of an additional component in the solution unnecessary.

**Figure 1:**
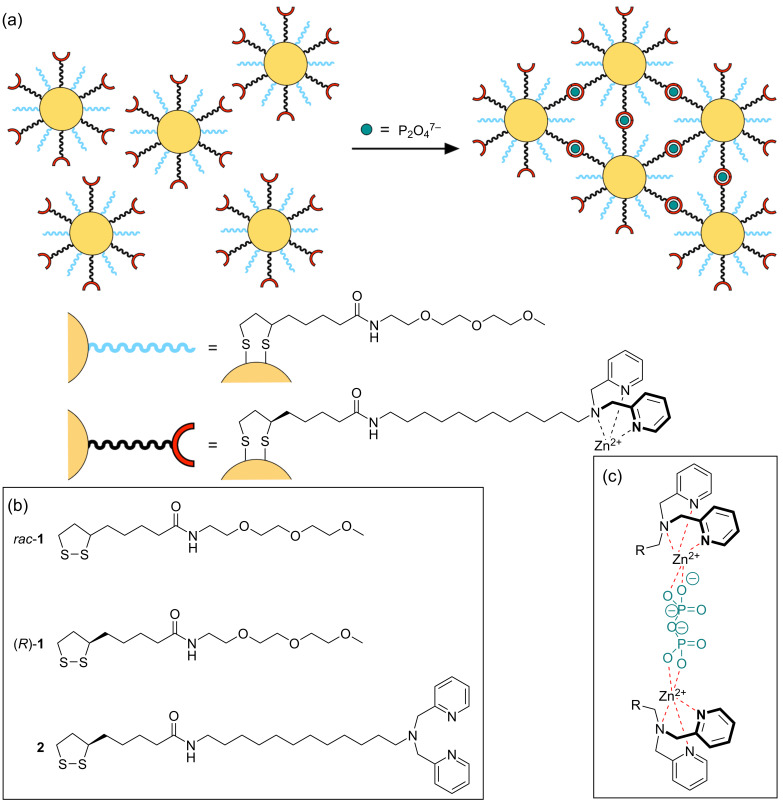
Schematic illustration of the analyte-induced crosslinking of gold nanoparticles containing a mixture of ligands of which one contains a peripheral zinc(II)–dipicolylamine unit while the other one mainly serves to control the degree of functionalization and nanoparticle solubility (a). The structures of the ligands **1** and **2** used in this work are shown in (b) and the potential mode of binding of two zinc(II)–dipicolylamine complexes to a diphosphate anion in (c).

This approach has several advantages: one is the flexibility with respect to the receptor units that can be used to mediate the anion recognition, which makes it possible to develop probes for anions other than sulfate. The only prerequisite is that the anion binding must involve more than one receptor unit to induce nanoparticle crosslinking (Figure 1). The use of mixed monolayer-protected AuNPs moreover allows varying the ratio of the ligands on the nanoparticle surface, of which one is responsible for the analyte recognition, while the other serves to dilute the receptor units on the surface to such an extent that analyte binding to units residing on the same nanoparticle becomes unlikely. The number of receptors should still be high enough to allow nanoparticle crosslinking to benefit from multivalent interactions [19–21]. The unfunctionalized ligand furthermore mediates the nanoparticle solubility, preferentially in an aqueous environment that is often most suited for practical applications. Since we wondered whether this concept could be extended to receptor types other than cyclopeptides, we sought for recognition motifs that also involve the binding of an anion to two identical functional groups.

A prominent example is the coordination of diphosphate ions to zinc(II)–dipicolylamine complexes, which is most efficient if the anion binds to two metal centers (Figure 1) [22–25]. This mode of binding is strong even in aqueous solution, potentially giving rise to highly selective receptors if the two binding sites are arranged at a distance that allows for binding a diphosphate, but not a larger triphosphate anion. The peptide and cyclopeptide-derived receptors introduced by Jolliffe are examples [26] along with a range of receptors based on other scaffolds [22–25].

To test whether this binding motif induces AuNP crosslinking, we synthesized nanoparticles containing peripheral zinc(II)–DPA complexes together with a solubilizing triethylene glycol-based ligand in different ratios and studied their interaction with anions (Figure 1). It turned out that these nanoparticles indeed responded to the presence of diphosphate in water/methanol 1:2 (v/v) by precipitating from the solution. Since the distance of the recognition units was not controlled, triphosphate anions produced similar effects, while an only a weak response was observed to hydrogenphosphate (and hydrogenarsenate). Other oxoanions and halides induced no visible effects and also did not interfere in diphosphate and triphosphate sensing when simultaneously present.

It should be noted that other strategies of detecting diphosphate anions by using immobilized receptors exist. Solutions of AuNPs containing tetracationic resorcinarene-derived cavitands were, for example, also shown to respond to the presence of diphosphate by a color change [27] due to the known diphosphate affinity of such cavitands [28]. Self-assembled monolayers on gold containing a bis(carbazolyl)urea-derived receptor allowed the sensitive detection of diphosphate in water by surface plasmon resonance (SPR) [29]. Silica nanoparticles containing a dye coordinated to a bis(zinc(II)–DPA) receptor released the dye upon diphosphate binding [30], and thus producing an optical signal. The extent of aggregation of AuNPs containing peripheral phosphate groups in the presence of an externally added low molecular weight bis(zinc(II)–DPA) complex could be controlled by diphosphate anions, which in turn allowed correlating the optical properties of the nanoparticle solution with the anion concentration [31]. Similar effects were achieved by using a tripodal copper(II) complex as the external crosslinker [32–33]. Finally, the metal ion-induced aggregation of AuNPs containing DPA residues in chloroform or acetonitrile/water was reversed by the addition of diphosphate anions (which thus caused the disassembly rather than the assembly of the AuNPs) [34]. While the limit of detection was sometimes very good (in one case less than 0.2 μmol/L [31]), these systems mostly involved the combination of several components and the analyte was in some cases only detected indirectly. In contrast, sensing of the AuNPs described here is a direct consequence of the analyte-induced nanoparticle aggregation, not requiring additional dyes, metal ions, or further components, which could be an advantage for the sensing of a biologically relevant anions in an aqueous environment [35–36].

## Results and Discussion

**Ligand synthesis**. The ligands used in this work are depicted in Figure 1. All of them derived from lipoic acid, which was chosen as the anchor group because the immobilization of a cyclic disulfide on gold involves the formation of two Au–S bonds, causing the ligands to have a smaller tendency to migrate and a generally higher stability with respect to thiol-containing AuNPs [37–39]. A further advantage is the straightforward ligand synthesis, which does not require the use of protecting groups as in the case of thiols.

**Scheme 1:**
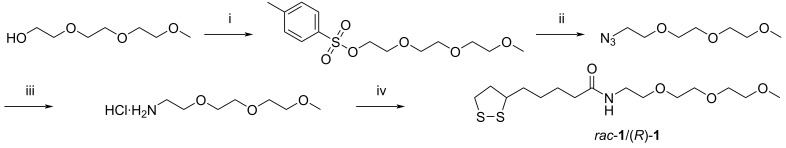
Syntheses of the ligands *rac*-**1** and (*R*)-**1**. Conditions: i) TsCl, NaOH, THF, 0 °C, 60 min → 25 °C, 80 min, 94%; ii) NaN_3_, acetone/water 5:1 (v/v), reflux 20 h, 95%; iii) H_2_, Pd/C (10%), 1 mol/L HCl (1 equiv), methanol, 25 °C, 8 d, 86%; iv) *rac*-lipoic acid (for *rac*-**1**) or (*R*)-lipoic acid (for (*R*)-**1**), TBTU, DIPEA, DMF, 25 °C, 6 d, *rac*-**1**: 51%, (*R*)-**1**: 44%.

The ligand **1** served as the solubilizing component and was synthesized in the racemic and the enantiomerically pure form. The racemate *rac*-**1** was obtained in four steps from triethylene glycol monomethyl ether by tosylation, substitution of the tosyl by an azide group, and reduction to obtain the corresponding amine (Scheme 1). This amine was coupled to racemic lipoic acid by using *O*-(1*H*-benzotriazol-1-yl)-*N*,*N*,*N*',*N*'-tetramethyluronium tetrafluoroborate (TBTU) as the coupling reagent. The yield in this step only amounted to 51% because the product had to be purified by preparative HPLC to obtain it in analytically pure form. The enantiomerically pure analog (*R*)-**1** was obtained in a similar fashion by using (*R*)-lipoic acid in the coupling reaction.

**Scheme 2:**
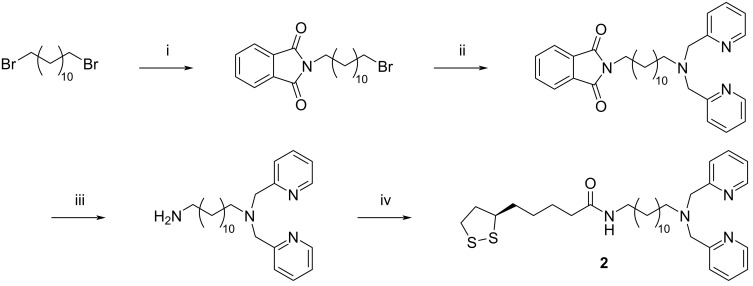
Synthesis of ligand **2**. Conditions: i) potassium phthalimide, DMF, 25 °C, 18 h, 67%; ii) 2,2'-dipicolylamine, K_2_CO_3_, KI, acetone, reflux 14 h, 50%; iii) N_2_H_4_⋅H_2_O, ethanol, 25 °C, 16 h, 56%; iv) (*R*)-lipoic acid, EDC⋅HCl, DMAP, CH_2_Cl_2_, 25 °C, 18 h, 52%.

The ligand **2** contained a peripheral 2,2'-dipicolylamine residue for zinc(II) coordination. This group was separated from the lipoic acid anchor group by a C_12_ chain to ensure that it had a sufficient distance from the nanoparticle surface after immobilization. Compound **2** was prepared by starting from potassium phthalimide and 1,12-dibromododecane (Scheme 2). The product resulting from this step was treated with bis(2-pyridylmethyl)amine to give a DPA derivative that was coupled to (*R*)-lipoic acid after deprotection to obtain **2** (synthetic details can be found in Supporting Information File 1). All products were obtained analytically pure and were fully characterized.

**Nanoparticle synthesis and characterization**. The AuNP precursor NP^cit^ was prepared by a modified Turkevich method that was reported to reliably afford nanoparticles with the desired diameter of 8–10 nm and a relatively narrow size distribution [40]. This method entailed the rapid addition of a hot aqueous solution of tetrachloroauric(III) acid to aqueous sodium citrate at 100 °C. After dialysis to remove excess citrate, the resulting solution was treated with a solution of a ligand or a mixture of ligands to exchange the protecting citrate molecules with the more strongly bound lipoic acid derivatives.

Initially, only *rac*-**1** was used as the ligand to establish a synthetic procedure for the nanoparticle preparation and to obtain information about the structure and properties of the products. The obtained nanoparticles NP*^rac^*^-^**^1^** were purified by using a combination of size exclusion chromatography and membrane filtration. They dispersed freely in water after isolation, giving a stable solution. The ^1^H NMR spectrum in D_2_O showed the typical broadened signals of the immobilized ligand molecules but no sharp signals, showing that the nanoparticles were not contaminated with unbound ligands or residual citrate. According to transmission electron microscopy (TEM), NP*^rac^*^-^**^1^** had an average diameter of 9.1 ± 2.4 nm and a maximum of the SPR band in the UV–vis spectrum at 528 nm (Figure S1 in Supporting Information File 1).

Further structural information was obtained by releasing the ligand molecules from the surface of NP*^rac^*^-^**^1^** with iodine, adding a known amount of 2,4,6-trimethoxy-1,3,5-triazine to the solution as an internal standard, and recording an ^1^H NMR spectrum. By relating the integral of the standard in the NMR spectrum to the integrals of the ligand signals, the amount of immobilized ligand was quantified. According to this experiment, the organic ligands made up ca. 5.3 ± 2.9% of the total mass of NP*^rac^*^-^**^1^**. The uncertainty of this measurement was large but nevertheless provided a rough estimate of the nanoparticle composition. The latter was calculated by assuming spherical nanoparticles with an average diameter of 9.1 nm, as determined by TEM. The AuNP volume thus amounted to 395 nm^3^ and the surface area to 260 nm^2^. Considering that the density of gold metal is 19.32 g/cm^3^, an average weight of 7.62 × 10^−9^ ng and a number of gold atoms of 23306 per AuNP resulted. From the determined gold-to-ligand mass ratio, we estimated that each particle contained on average 743 ± 426 ligands. Taking into account the surface area of the nanoparticles, the number of ligand molecules per nm^2^ hence amounted to 2.9 ± 1.6 or, in other words, each ligand occupied an area of 0.53 ± 0.34 nm^2^. The order of magnitude of these results is comparable to the footprint size of 0.21 nm^2^ reported for lipoic acid on 10 nm gold nanoparticles [41] and the number of citrate molecules per nm^2^ on citrate-protected AuNPs with a diameter of ca. 15 nm, which was determined to amount to 3.1 [42].

**Figure 2:**
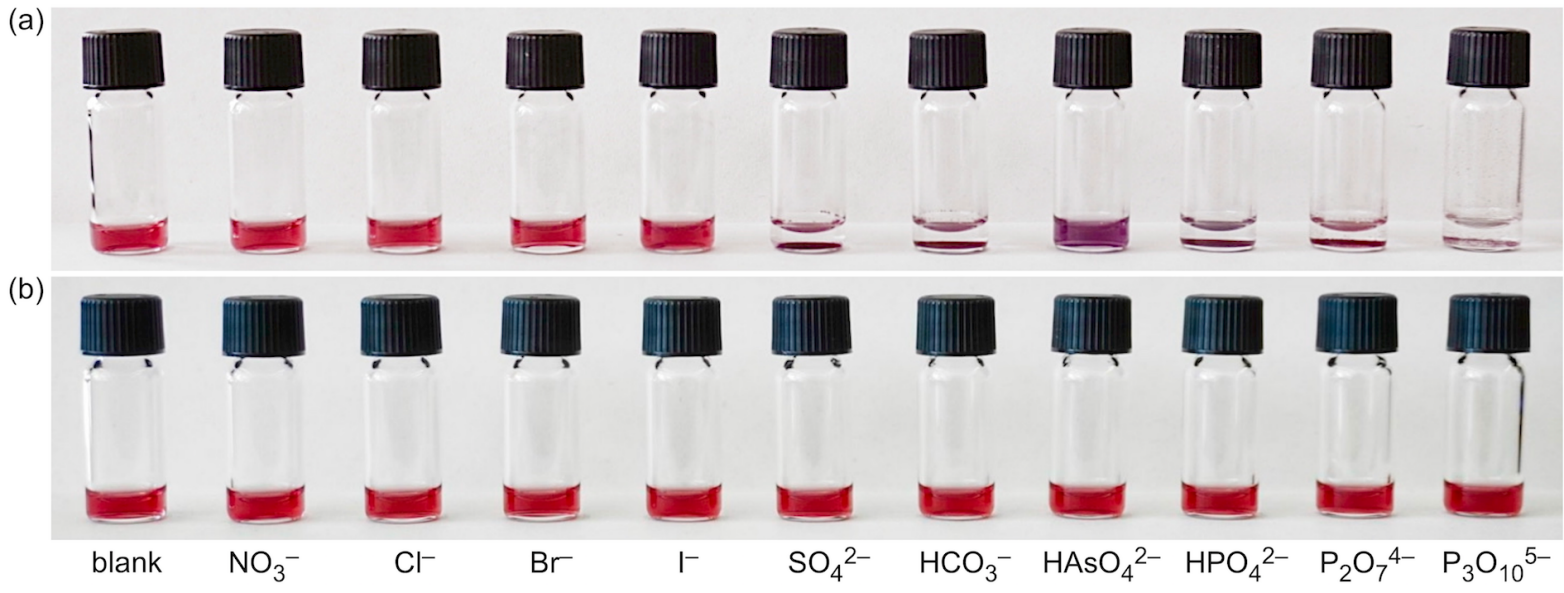
Photographs of solutions of NP*^rac^*^-^**^1^** in water (0.25 mg/mL) containing different sodium salts at a concentration of 50 mmol/L (a) and of solutions of NP^(^*^R^*^)-^**^1^** under the same conditions (b). The anions of the salts are specified below the vials and the counterion was sodium in all cases.

We also tested whether NP*^rac^*^-^**^1^** was stable in the presence of different salts. To this end, solutions of NP*^rac^*^-^**^1^** in water (0.25 mg/mL) were prepared to which solutions of sodium salts were added to reach a final salt concentration of 50 mmol/L. This concentration was chosen to ensure that the nanoparticles were stable even at ionic strengths much higher than that expected in the final assays. It turned out that the solutions were stable when they contained halides and nitrate but that the nanoparticles precipitated in the presence of the other tested oxoanions (Figure 2a). The effect of phosphates on nanoparticle solubility was particularly undesirable because it could potentially lead to unspecific responses. We therefore tested whether using the enantiomerically pure (*R*)-**1** as ligand would produce a different outcome.

The corresponding nanoparticles NP^(^*^R^*^)-^**^1^** were prepared in a similar fashion as NP*^rac^*^-^**^1^**. The characterization showed that they were pure, had an average diameter of 8.5 ± 2.1 nm, similar to the one determined for NP*^rac^*^-^**^1^**, and exhibited an SPR band when dissolved in water with a maximum at 528 nm (Figure S2 in Supporting Information File 1). Moreover, the solutions turned out to be stable even if those salts were present at a concentration of 50 mmol/L that caused the precipitation of NP*^rac^*^-^**^1^** (Figure 2b). At the moment, we have no conclusive explanation for the different behaviors of NP^(^*^R^*^)-^**^1^** and NP*^rac^*^-^**^1^**. The solutions of both nanoparticles were stable at low millimolar concentrations of all of the tested salts (data not shown). Differences in the behavior only materialized at high ionic strengths and in the presence of certain anions. We could not reliably detect differences in the composition of the two nanoparticles because of the large uncertainties associated with our method (see Supporting Information File 1), and we therefore could not exclude that NP^(^*^R^*^)-^**^1^** and NP*^rac^*^-^**^1^** differed in the composition. Alternatively, the organization of the ligands on the nanoparticles could also have been different. These structural differences were presumably small, causing the properties of the AuNPs derived from racemic and enantiomerically pure lipoic acid derivatives to be indistinguishable under most conditions. In light of the distinct response of NP*^rac^*^-^**^1^** to the presence of oxoanions we still felt more comfortable to continue working with the AuNPs containing the enantiomerically pure ligands.

We thus continued with the preparation of the mixed monolayer-protected AuNPs by subjecting the citrate-protected nanoparticles to mixtures of ligands (*R*)-**1** and **2**. In our first attempts to perform the exchange reaction, the aqueous solution of NP^cit^ was treated with a solution of (*R*)-**1** and **2** in methanol. Unfortunately it turned out that **2** was insufficiently soluble in the resulting solvent mixture. The ligand thus precipitated and did not react. We tried to address this solubility issue by increasing the fraction of the organic solvent during the exchange reaction, but the products thus obtained also did not contain immobilized **2** according to the ^1^H NMR spectroscopic characterization. For similar reasons, the simultaneous immobilization of (*R*)-**1** and the cyclopeptide-derived ligand used in our previous work had also failed, and we therefore had to use a two-step immobilization procedure to obtain the respective mixed monolayer-protected AuNPs [18]. Here, we tested whether performing the immobilization at a lower pH value at which **2** was partially protonated and therefore better water-soluble would allow immobilizing it together with (*R*)-**1**. As acid, we initially used diluted nitric acid because the eventual formation of the zinc complexes with zinc(II) nitrate would anyway lead to AuNPs containing nitrate anions. Adding the acid to the NP^cit^ solution prior to the addition of the ligand mixture caused the AuNPs to precipitate, likely because the protecting citrate molecules were protonated. Therefore, the reaction mixture was adjusted to pH 3 with 0.1 mol/L nitric acid shortly after adding the solution containing the ligands in methanol. A color change of the reaction mixture to purple was observed after the addition of the acid but the solution remained homogeneous. The nanoparticles were isolated after overnight equilibration at room temperature and characterized by ^1^H NMR spectroscopy. The presence of signals of aromatic residues in the corresponding spectra confirmed the successful immobilization of **2**. Unfortunately, it turned out that the extent of immobilization was not well reproducible, with reactions performed by using the same ratio of the two ligands affording nanoparticles with varying ratios of the immobilized ligands. One reason for the unsatisfactory reproducibility of this strategy was likely that the exact moment at which nitric acid was added had a strong effect on the extent to which **2** reacted with NP^cit^, and ensuring that the acid was always added at exactly the same moment was difficult.

We therefore considered the use of another acid and tested citric acid, which was attractive because we expected this acid to produce a buffer together with the citrate present on the nanoparticles, which should reduce the influence of possible pH changes on the exchange reaction. In this case, adjusting the aqueous solution of NP^cit^ to pH 3 by adding citric acid did not cause nanoparticle precipitation, allowing the addition of the acid prior to the ligand. The solution moreover remained homogeneous after the ligand solution was added. After equilibrating for 16 h at room temperature, the nanoparticles were isolated in the usual fashion and characterized. The presence of aromatic signals in the ^1^H NMR spectra of the products again confirmed the successful immobilization of **2**. Relating the integrals of these signals to those of prominent signals of (*R*)-**1** furthermore allowed determining the ratio of the two surface-bound ligands. This ratio turned out to be well reproducible for reactions performed under identical conditions, allowing us to control the surface composition of the products by varying the ratio of (*R*)-**1** and **2** in the exchange reactions (for details, see Supporting Information File 1). Four mixed monolayer-protected AuNPs were thus prepared, namely, NP^4^, NP^10^, NP^25^, and NP^35^, containing, ratios of the surface bound ligands (*R*)-**1**/**2** of 96:4, 90:10, 75:25, and 65:35, respectively (Figures S3–S6 in Supporting Information File 1). Based on our estimation of the composition of NP*^rac^*^-^**^1^**, these nanoparticles thus contained on average 30, 75, 190, and 260 DPA units, respectively. In all cases, the relative amount of the functionalized ligand **2** in the products was lower than that used during the exchange reaction, indicating that (*R*)-**1** was more prone to react with NP^cit^ than **2**.

**Figure 3:**
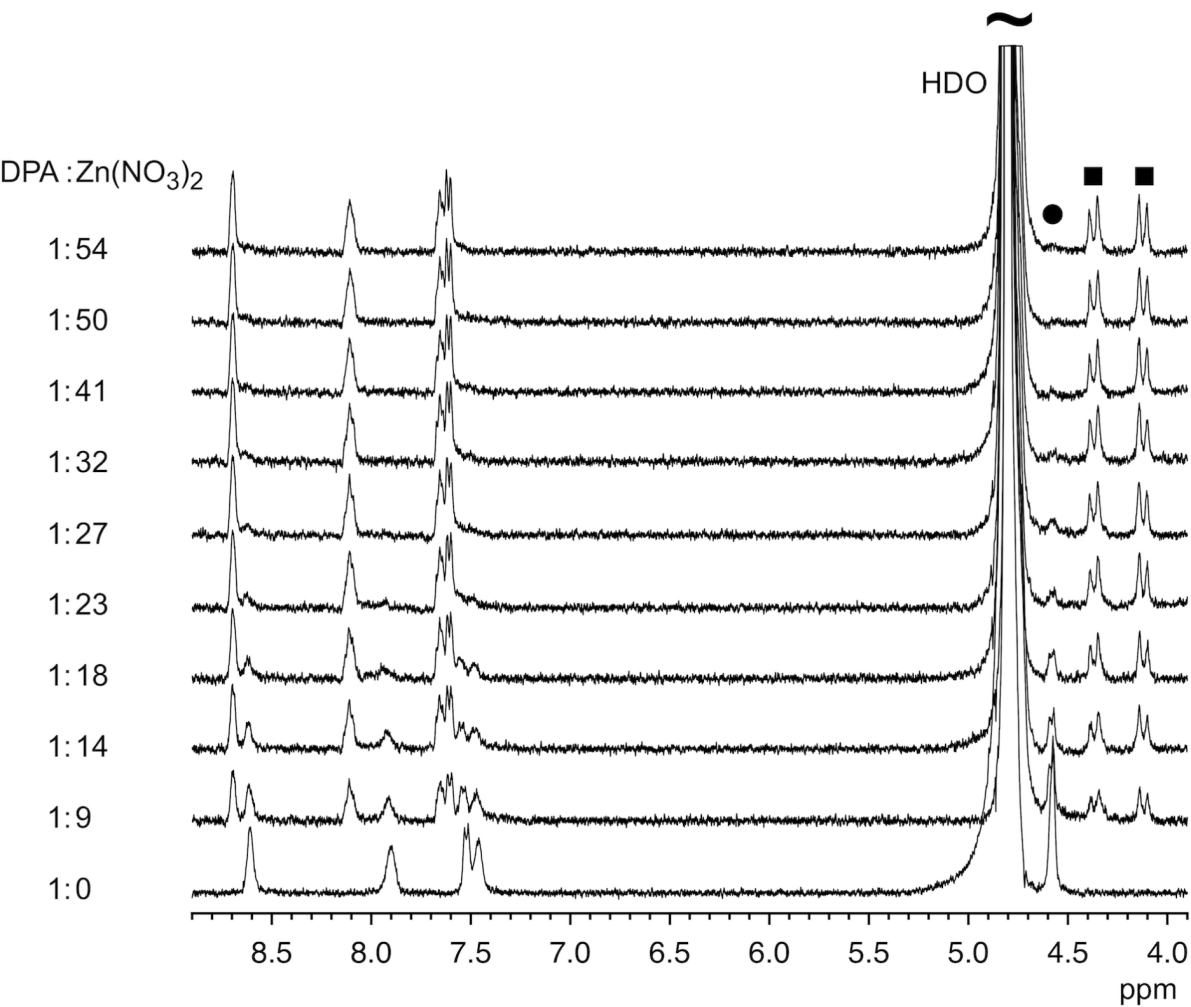
Sections of the ^1^H NMR spectra of solutions of NP^25^ in D_2_O/CD_3_OD 1:2 (v/v) between 8.9 and 3.9 ppm containing increasing amounts of Zn(NO_3_)_2_. The equivalents of Zn(NO_3_)_2_ per estimated number of DPA units are specified for each spectrum. The CH_2_ signals in uncomplexed and complexed DPA units are marked with a circle and with squares, respectively.

The final synthetic step involved converting the free DPA residues on the nanoparticle surface into the corresponding zinc(II) complexes. To ensure the successful metal complexation and to determine the minimum amount of zinc(II) nitrate required for a complete conversion, we followed the ^1^H NMR spectroscopic effects of the addition of a 0.1 mol/L zinc(II) nitrate solution to a solution of a known amount of NP^25^ in D_2_O/CD_3_OD 1:2 (v/v). In the absence of the zinc salt, the signals of the methylene groups of the DPA moiety of **2** appeared in the spectrum as a singlet at ca. 4.5 ppm (Figure 3). With the progressive addition of zinc(II) nitrate, this signal became smaller and two doublets appeared at lower ppm values. Once the concentration of the zinc salt exceeded a certain value, only these doublets were still visible. In addition, zinc complexation also affected the aromatic signals of the DPA ligands.

The spectral changes observed in the spectra confirmed that zinc complexation had occurred under the chosen conditions. The change of the multiplicity of the methylene signal accounted for the rigidification of the DPA moiety during metal binding, which rendered the corresponding protons diastereotopic. The rate of metal exchange was moreover slow on the NMR time scale and the reaction was complete once the zinc salt concentration amounted to at least 16 mmol/L, which was 50 times higher than the estimated concentration of the DPA moieties in the nanoparticle solution (see Supporting Information File 1).

We used this value to estimate the minimum amount of zinc(II) nitrate required to fully convert the DPA moieties on the surfaces of the different nanoparticles used in this work into their respective complexes. The formation of the zinc(II)–DPA-containing complexes thus involved dissolving the nanoparticles in D_2_O/CD_3_OD 1:2 (v/v) and treating the solution with 50 equiv of zinc(II) nitrate per estimated number of DPA groups. An ^1^H NMR spectrum of the mixture was recorded after 30 min to confirm that complex formation had occurred and was complete. The solution was then evaporated and the nanoparticles redissolved in water/methanol 1:2 (v/v) to afford the concentrations in the subsequent binding studies. The corresponding solutions thus contained residual unbound zinc(II) nitrate. Furthermore, we could not rule out that a partial dissociation of the surface-bound zinc complexes had occurred because the nanoparticle concentration in the binding studies was lower than that used for the zinc complexation. We nevertheless decided against using an even larger excess of the zinc salt because of potential interferences of salts in the binding studies containing anions that form sparingly soluble zinc salts. The nanoparticles obtained are denoted NP^4-Zn^, NP^10-Zn^, NP^25-Zn^, and NP^35-Zn^ in the following.

**Figure 4:**
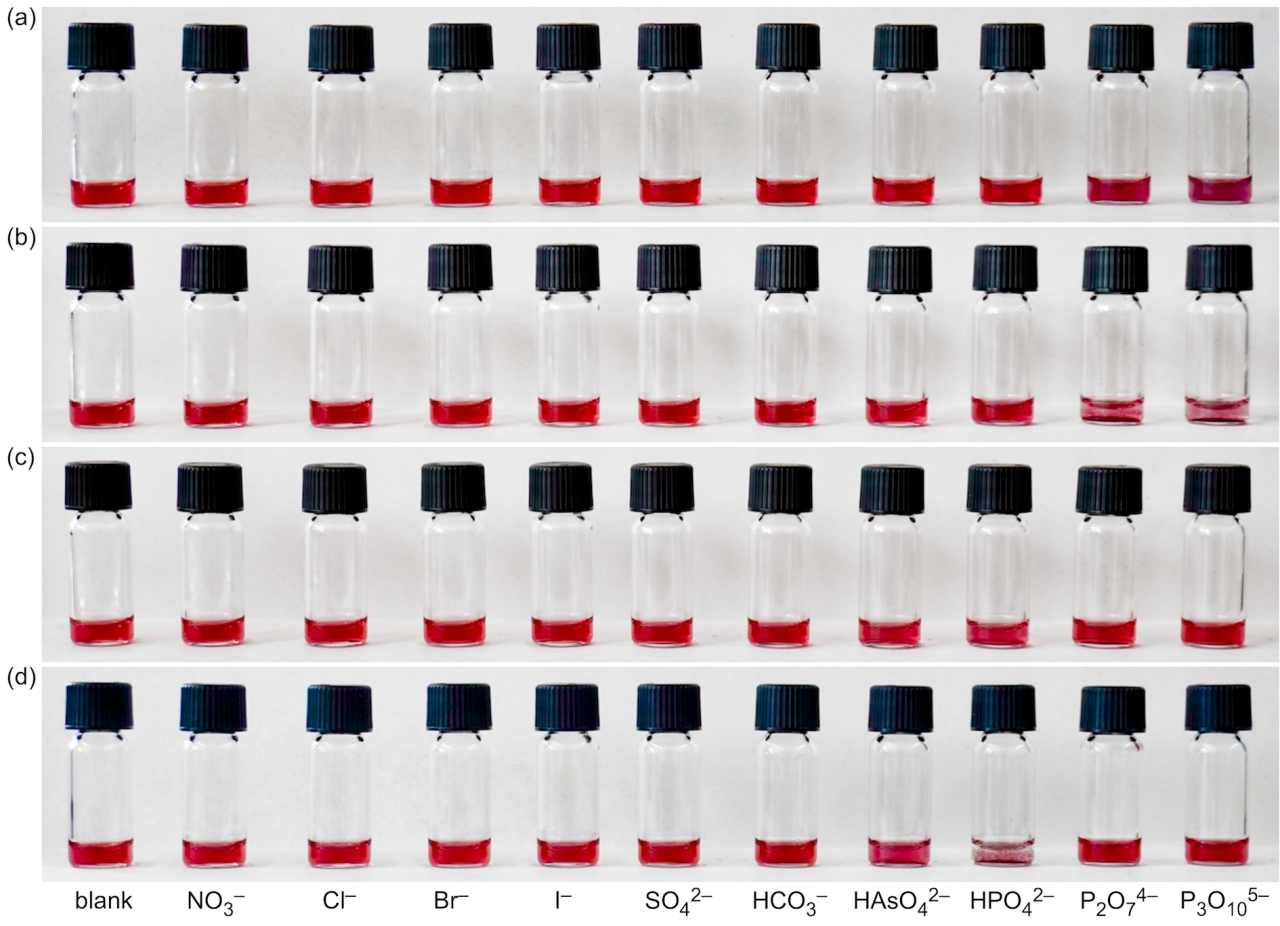
Images of vials containing solutions of NP^10-Zn^ (0.25 mg/mL) in water/methanol 1:2 (v/v) and additional sodium salts of the anions specified in the bottom row at concentrations of 99 μmol/L (a), 196 μmol/L (b), 291 μmol/L (c), and 476 μmol/L (d). The photos were taken 5 min after each salt addition.

**Binding studies**. The binding studies were performed in water/methanol 1:2 (v/v) because of an insufficient solubility of the AuNPs in water. Initially, we evaluated qualitatively whether the addition of the sodium salts of various anions induced visual changes in the solutions of the functionalized AuNPs. To this end, a nanoparticle solution, which was colored bright red in the absence of anions, was distributed over eleven glass vials. Then, aqueous solutions of the sodium salts of various anions (NO_3_^−^, Cl^−^, Br^−^, I^−^, SO_4_^2−^, HCO_3_^−^, HAsO_4_^2−^, HPO_4_^2−^, P_2_O_7_^4−^, P_3_O_10_^5−^) were added and the effects inspected. Note that all solutions additionally contained uncomplexed Zn^2+^ ions and the nitrate counterions. The images obtained for NP^10-Zn^ are depicted in Figure 4. They show that the first addition, leading to a salt concentration of 99 μmol/L, caused the solutions to which diphosphate and triphosphate were added to darken and acquire a purple color. Further increasing the salt concentrations induced nanoparticle precipitation in the same solutions. At even higher salt concentrations, the precipitates redissolved again and at the highest anion concentration, the nanoparticles in the solution containing sodium hydrogenphosphate precipitated. Only HAsO_4_^2−^ also caused the nanoparticle solution to become turbid at a high salt concentration, but no effects were observed in the presence of the other anions at any concentration, even when the solutions were kept overnight.

The same experiment was performed with NP^(^*^R^*^)-^**^1^**, lacking the zinc(II)–DPA recognition motifs. In this case, none of the nanoparticle solutions reacted to any of the salts (Figure S7 in Supporting Information File 1), demonstrating that the effects of the phosphates were related to the presence of the surface-bound zinc complexes. To ensure that the precipitation of the AuNPs after the addition of the phosphate salts was not due to the formation of insoluble zinc phosphates, a solution of NP^(^*^R^*^)-^**^1^** (0.21 mg/mL) was prepared, containing Zn(NO_3_)_2_ at a concentration of 8.3 mmol/L, significantly higher than the Zn(NO_3_)_2_ concentration in the solution of the functionalized AuNPs. The addition of one equivalent of Na_4_P_2_O_7_ caused the precipitation of a white solid. The solution remained red, however, indicating that insoluble zinc salts could not have been responsible for the precipitation of the functionalized AuNP in the presence of phosphates (Figure S11, Supporting Information File 1).

Similar binding studies were performed with the other nanoparticles. The images in Figures S8–S10 (Supporting Information File 1) show that, although all nanoparticles behaved qualitatively similar in that they only responded to hydrogenphosphate, diphosphate, and triphosphate, the anion concentrations necessary to induce visual changes depended sensitively on the extent of the surface functionalization. NP^4-Zn^ with the lowest amount of surface-bound recognition units, turned out to be slightly more sensitive than NP^10-Zn^. In this case, the presence of phosphates mainly caused color changes of the solution, but fine precipitates were also visible. These precipitates appeared when the concentrations of diphosphate and triphosphate amounted to 10 μmol/L, and disappeared at a concentration of 196 μmol/L in the case of diphosphate while a concentration of 476 μmol/L was required for triphosphate. The solutions containing hydrogenphosphate (and also that containing hydrogenarsenate) darkened already at 291 μmol/L. The solutions of NP^25-Zn^, in contrast, exhibited no changes at low anion concentrations, with nanoparticle precipitation only occurring when diphosphate and triphosphate were present at 291 μmol/L. Thus, this nanoparticle required a concentration of diphosphate and triphosphate to precipitate at which NP^4-Zn^ already responded to hydrogenphosphate. At an anion concentration of 476 μmol/L, NP^25-Zn^ was fully dissolved in all solutions and this nanoparticle only responded to hydrogenphosphate at even higher concentrations (vide infra). No pronounced effects were observed for NP^35-Zn^ at any anion concentration.

**Figure 5:**
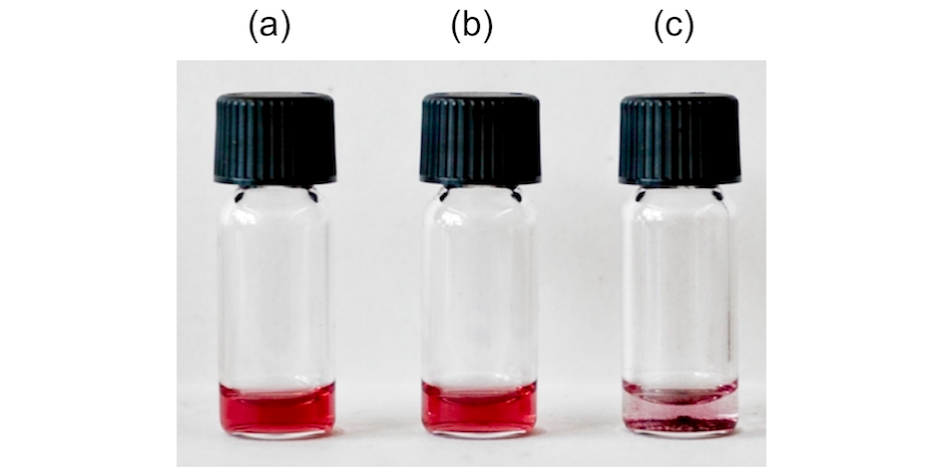
Photograph of the solutions of the competition experiment. Vial (a) only contained NP^10-Zn^ (and therefore also NO_3_^−^, 0.25 mg/mL), vial (b) additionally contained NaCl (1.4 mmol/L), NaNO_3_, (>1.4 mmol/L), and Na_2_SO_4_ (1.4 mmol/L), and vial (c) NaCl, NaNO_3_, and Na_2_SO_4_ at the aforementioned concentrations as well as Na_4_P_2_O_7_ (0.14 mmol/L).

To assess whether the diphosphate-induced nanoparticle precipitation was affected in the presence of simultaneously present competing anions, a solution of NP^10-Zn^ in water/methanol 1:2 (v/v) was prepared additionally containing NaCl, NaNO_3_, and Na_2_SO_4_ at concentrations of 1.4 mmol/L each. This solution expectedly did not react to the presence of the anions, consistent with the above results. Only after the addition of Na_4_P_2_O_7_ to give a diphosphate concentration of 0.14 mmol/L did the nanoparticles precipitate (Figure 5). Thus, neither chloride, nitrate (which partly already derived from the added Zn(NO_3_)_2_), nor sulfate interfered with diphosphate sensing, even when simultaneously present at concentrations significantly higher than the actual analyte.

**Figure 6:**
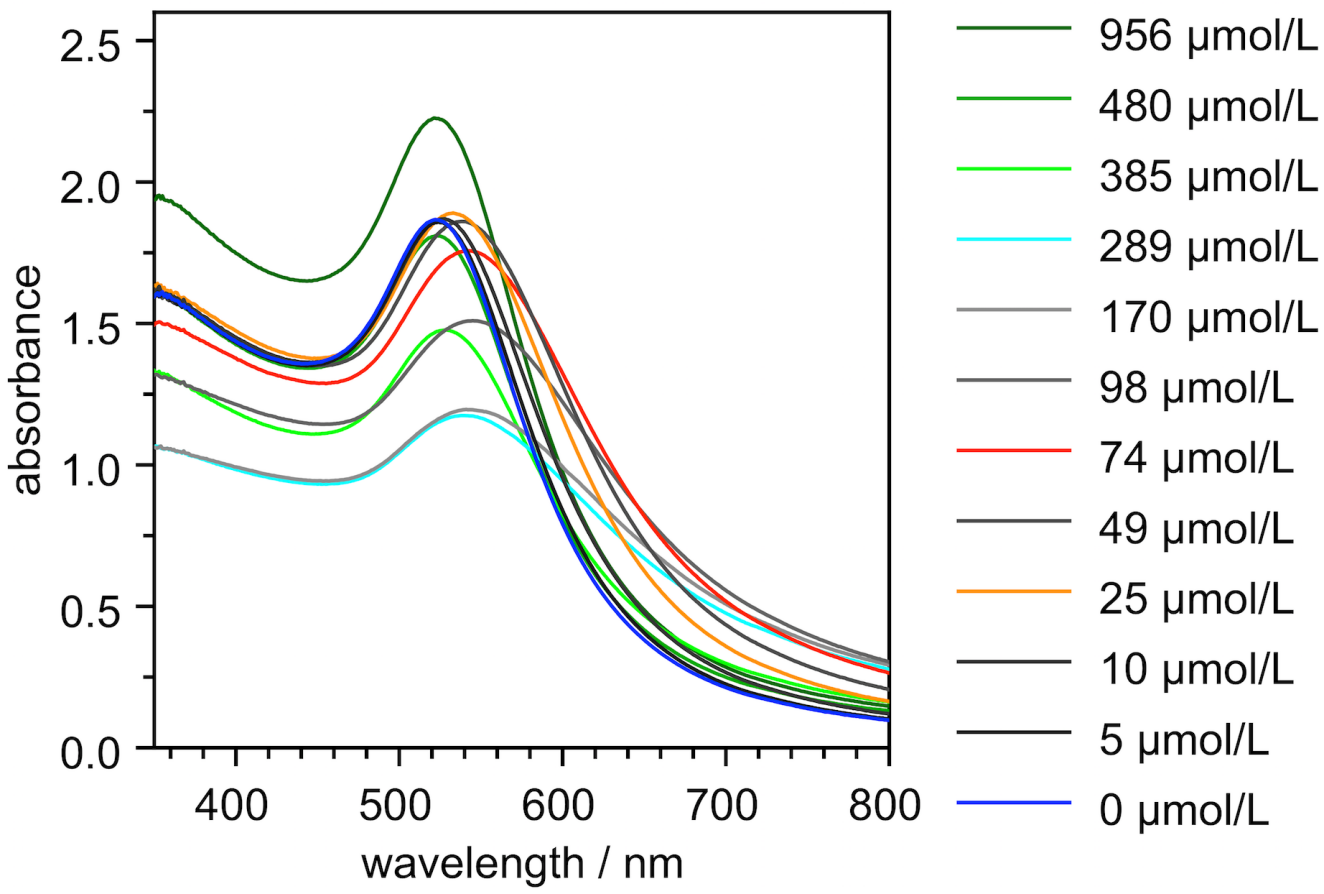
UV–vis spectra of NP^10-Zn^ (0.25 mg/mL in the initial measurement) in water/methanol 1:2 (v/v) containing between 0 and 956 μmol/L of Na_4_P_2_O_7_. The spectra were measured 10 min after each salt addition.

The effects of the phosphate salts on the solutions of all four nanoparticles were then followed in a more precise fashion by using UV–vis spectroscopy. To this end, the solutions of the four nanoparticles NP^4-Zn^, NP^10-Zn^, NP^25-Zn^, and NP^35-Zn^ (0.25 mg/mL) in water/methanol 1:2 (v/v) were treated with increasing amounts of either Na_2_HPO_4_, Na_4_P_2_O_7_, or Na_5_P_3_O_10_, and the UV–vis spectra of the resulting solutions were recorded between 350 and 800 nm to assess the effects of the salts on the position and intensity of the SPR band. Figure 6 shows the series of spectra obtained for the titration of NP^10-Zn^ with diphosphate to illustrate the typical course of such a measurement.

The first additions of the salt did not produce major changes in the AuNP spectrum. Since these additions caused a slight reduction of the AuNP concentration, a minor decrease of the intensity of the SPR band was mostly observed although the band sometimes also became slightly stronger in spite of the concentration decrease. Once a certain concentration was reached, the solutions started to appear purple, which was reflected in the UV–vis spectra in a red shift of the SPR band. The corresponding spectrum is shown in orange in Figure 6. At somewhat higher concentrations, the intensity of the SPR band dropped because nanoparticle precipitation started to set in (red spectrum) and a further increase of the salt concentration induced AuNP redissolution, which was accompanied by an increase in the intensity of the SPR band and a shift of its maximum back to the wavelength at which the salt-free AuNP initially absorbed (green spectra). The minimum concentrations at which the red shift of the SPR band was observed and that at which the redissolution of the precipitates occurred are summarized for all investigated nanoparticles in Table 1.

**Table 1:** Minimum salt concentrations required to precipitate and to subsequently redissolve NP^4-Zn^, NP^10-Zn^, NP^25-Zn^, and NP^35-Zn^ from solutions in water/methanol 1:2 (v/v) at a nanoparticle concentration of 0.25 mg/mL.

AuNP	*c*(Na_2_HPO_4_) / μmol/L		*c*(Na_4_P_2_O_7_) / μmol/L		*c*(Na_5_P_3_O_10_) / μmol/L	
	color change	dissolution	color change	dissolution	color change	dissolution

NP^4-Zn^	74	n.d.^a^	10	196	10	391
NP^10-Zn^	268	361	25	385	5	385
NP^25-Zn^	484	n.d.^a^	196	388	49	388
NP^35-Zn^	n.d.^a^	n.d.^a^	n.d.^a^	n.d.^a^	489	973

^a^not detected.

While the general course of all titrations was similar, characteristic effects of the degree of functionalization on the behavior of the nanoparticles were observed. For NP^4-Zn^, diphosphate and triphosphate concentrations of 10 μmol/L were sufficient to cause a red shift of the SPR band (Figure S12 in Supporting Information File 1). The band then decreased in intensity, which is a typical indication of nanoparticle precipitation, and subsequently moved back to the original wavelength at concentrations of diphosphate and triphosphate exceeding, respectively, 196 μmol/L and 391 μmol/L, consistent with the results of the visual binding study. NP^4-Zn^ also responded to the presence of HPO_4_^2−^ anions, although a significantly higher concentration was needed for nanoparticle precipitation to set in than in the case of P_2_O_7_^4−^ or P_3_O_10_^5−^. No redissolution was observed for the precipitate formed in the presence of hydrogenphosphate even at the highest anion concentration used.

The sensitivity of NP^10-Zn^ to P_3_O_10_^5−^ and P_2_O_7_^4−^ was similar or only slightly lower than that of NP^4-Zn^, but HPO_4_^2−^ had to be present at a higher concentration than in the case of NP^4-Zn^ to produce a color change and the concomitant nanoparticle precipitation. All precipitates redissolved when the solutions exceeded a concentration of ca. 380 μmol/L (Figure S13 in Supporting Information File 1). Even higher concentrations were needed to cause NP^25-Zn^ to respond to the presence of the anions. The precipitates moreover only redissolved in the presence of diphosphate and triphosphate but not with hydrogenphosphate (Figure S14, Supporting Information File 1). The addition of HPO_4_^2−^ or P_2_O_7_^4−^ to NP^35-Zn^ did not cause notable changes in the position of the SPR band or the formation of a precipitate, while the effects were minor and only visible at relatively high concentrations for P_3_O_10_^5−^ (Figure S15 in Supporting Information File 1).

**Figure 7:**
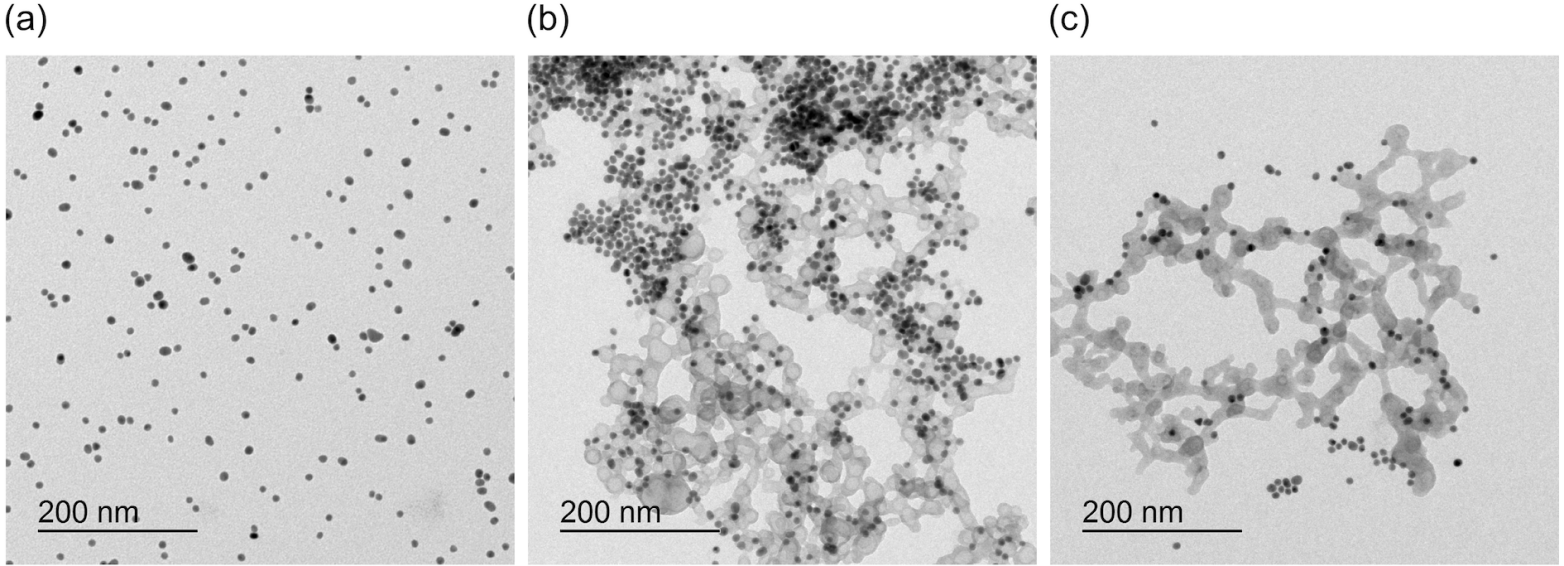
TEM images of NP^10-Zn^ (0.25 mg/mL) in water/methanol 1:2 (v/v) before (a) and after the addition of Na_4_P_2_O_7_ to give a concentration of 148 μmol/L (b). The image in (c) was obtained after increasing the diphosphate concentration to 476 μmol/L. Further images are depicted in Figure S16 of Supporting Information File 1.

Thus, we observed clear correlations between the selectivity and sensitivity of the AuNPs and the nature of the anion and the number of binding sites on the AuNP surface. All nanoparticles only responded to phosphates, consistent with the known affinity of zinc(II)–dipicolylamine complexes to these anions [22–25]. In the presence of these anions, the nanoparticle solutions initially changed color at a certain limiting concentration, indicating that the anions induced crosslinking and the formation of aggregates in which individual AuNPs were electronically coupled. We did not observe a continuous shift of the optical properties with the anion concentration, however, because the aggregates were stable only in a narrow concentration range. A further salt addition caused the nanoparticles to precipitate presumably because they became insoluble when they increased in size. To confirm this assumption, we also followed the effect of the salts on the AuNPs by TEM. According to the images obtained for NP^10-Zn^, the salt-free solution contained individually dispersed AuNPs as expected (Figure 7a). At a diphosphate concentration of 148 μmol/L, large aggregates of the nanoparticles were observed, consistent with the bluish color of the solution and the shift of the SPR band to a longer wavelength. A network of a lighter material also appeared in these images, which could indicate the presence of insoluble zinc(II) diphosphate (Figure 7b). The TEM images of a solution of NP^(^*^R^*^)-^**^1^** to which Zn(NO_3_)_2_ and sodium diphosphate were sequentially added indeed featured similar structures (Figure S17 in Supporting Information File 1). In the case of this nanoparticle, however, the AuNPs were not aggregated after diphosphate addition. TEM thus confirmed that nanoparticle aggregation was linked to the presence of the surface-bound receptors. Whether the aggregates themselves were insoluble or whether insoluble zinc salts contributed to the precipitation was difficult to distinguish, although the fact that the nanoparticle aggregates were usually found in the TEM images close to or even inside the lighter material suggested that the latter could be the case. Aggregates surrounded by zinc salts nevertheless remained responsive to the salt concentration because the increase of the diphosphate concentration to 476 μmol/L caused the AuNPs to mostly dissociate while the insoluble zinc salts remained (Figure 7c).

The formation of insoluble zinc salts accompanying aggregation, which was a consequence of the excess of zinc(II) nitrate that had to be used to form the surface-bound complexes, possibly interfered with the anion sensing. The estimation of the concentration of free Zn(NO_3_)_2_ in the nanoparticle solutions indicated, however, that the concentrations of free zinc ions in the solutions were always higher than the anion concentration required to induce a response of the AuNPs (Table S5 in Supporting Information File 1). The solutions of NP^10-Zn^ had a zinc(II) concentration of ca. 160 μmol/L but responded to diphosphate already when the concentration of the anion was only 25 μmol/L. Thus, although we cannot exclude an effect of the free zinc salt on the nanoparticle behavior, the minimum concentration of the anion required to induce aggregation was primarily governed by the nanoparticle composition. This aggregation moreover occurred for all nanoparticles in a defined concentration window, in which there was a relatively sharp transition from the dissolved to the aggregated state. This behavior could be attributed to the multivalent nature of the AuNPs that caused nanoparticle crosslinking to benefit from cooperative effects of the surface-bound receptor units [19–21]. Once initial linkages between individual nanoparticles were formed, interactions between the remaining vacant binding sites were facilitated, leading to the reinforcement of the aggregates. These aggregates were still susceptible to changes in the anion concentration since they dissociated when the anion concentration exceeded a certain value. The redissolution was likely caused by a shift of the binding mode from 2:1 complexes, in which two zinc(II)–dipicolylamine residues bind to one anion, to 1:1 complexes. We cannot exclude, however, that the metal ion was stripped from the zinc(II)–dipicolylamine at high phosphate concentrations and that insoluble zinc complexes were formed, which could also have been the reason for the observed nanoparticle dissociation.

Independent of the exact nature of this process, anion sensing was associated with a lower anion concentration, at which the AuNPs started to aggregate, and an upper limit, at which the aggregates dissociated, at least in the case of the more strongly bound diphosphate and triphosphate anions. For hydrogenphosphate, some of the nanoparticles only precipitated at a certain concentration but did not dissolve even at high hydrogenphosphate concentrations. The respective concentration limits depended on the nature of the anion and on the nanoparticle composition. With respect to the anion, the AuNP sensitivity increased from hydrogenphosphate over diphosphate to triphosphate, likely because of the increasing negative charge of the anion in this direction that strengthened the interactions. In addition, the sensitivity also depended on the surface composition of the AuNPs, with the sensitivity increasing as the number of surface-bound binding sites went down. A large number of surface-bound ligands therefore turned out to be detrimental for the sensitivity, maybe because efficient crosslinking required a large number of anions to overcompensate potential repulsive interactions between unused surface-bound zinc complexes when the degree of the surface functionalization was high. However, the weak and unspecific response of NP^35-Zn^ to diphosphate and triphosphate could also have been due to the extensive formation of unproductive complexes in which two ligands bound to the same nanoparticle engaged in phosphate binding. Favoring interparticle crosslinking over intraparticle interactions thus improved the sensing properties of the mixed monolayer-protected AuNPs. Varying the nature of the metal center could be an alternative means to fine-tune the behavior, which will be investigated in the future.

## Conclusion

Concluding, our work demonstrated that immobilizing recognition units on the surface of gold nanoparticles that form 2:1 complexes with anions represents a promising strategy for the development of optical probes. While the previously described nanoparticle-based probe containing cyclopeptides as recognition units only responded to sulfate [18], the high affinity of zinc(II)–dipicolylamine complexes for phosphate-derived anions caused the nanoparticles developed in this work to respond to the presence of hydrogenphosphate, diphosphate, and triphosphate (and in some cases also to HAsO_4_^2−^ because of the close relationship of this anion to HPO_4_^2−^) but to none of the eight other anions tested. Thus, anion selectivity was clearly controlled by the nature and the binding properties of the immobilized receptors. The sensitivity of the detection increased with an increasing charge of the phosphates but did not depend on additional structural parameters as in many low-molecular-weight phosphate probes, in which the distance of the two metal centers on a rigid scaffold contributes to anion selectivity. As a consequence, the diphosphate vs triphosphate selectivity of the nanoparticles was not very pronounced.

An important structural aspect distinguishing the AuNPs presented here from many other nanoparticle-based probes is that they not only contained the ligand responsible for the substrate recognition but an additional ligand that allowed controlling the number of active residues on the surface. This structural feature allowed us to assess how the degree of surface functionalization affects the sensitivity, and we found in this context that nanoparticles with fewer binding sites were more sensitive than those containing a larger percentage of the receptor units. The reason was likely that the increase in the number of surface-bound receptors also increased the possibility of the formation of unproductive complexes by receptors located on the same nanoparticle.

The anion-induced optical changes of the nanoparticle solution, moreover, did not shift continuously as the anion concentration increased but occurred in a relatively narrow concentration regime in which the nanoparticles underwent the transition from the nonaggregated to the aggregated state. This behavior is typical for multivalent systems in which individual receptor units act in a cooperative fashion, indicating that the simultaneous participation of the surface-bound receptor units in anion binding had a crucial effect on the AuNP behavior.

The lower limiting diphosphate and triphosphate concentrations that could be detected with the most sensitive AuNPs NP^4-Zn^ and NP^10-Zn^ amounted to 10 μmol/L, which compares favorably to the detection limit of many other diphosphate probes [22–26]. Diphosphate concentrations in this range are found in urine and saliva [43–44] but up to two orders of magnitude lower detection limits are required to determine diphosphate in blood plasma [45]. These nanoparticles moreover featured an upper analyte concentration at which the initially formed precipitates again dissolved. With the cyclopeptide-modified nanoparticles, we did not observe the dissolution of the precipitates formed in the presence of sulfate anions (at least not in the investigated concentration range), likely because a large thermodynamic driving force exists for the 1:1 cyclopeptide complexes to recruit the second cyclopeptide ring. The thus formed 2:1 complexes are therefore not very prone to dissociate again, which is likely different for the zinc(II)–dipicolylamine complexes with phosphates. In the context of sensing, these nanoparticles thus have the characteristic feature of not only qualitative reporting the presence of a certain anion but also whether the respective solution contains the analyte within a certain concentration range.

A better understanding of the effect of the surface composition of such nanoparticles on the sensitivity of detection and the cooperativity of the receptor units is now required to better control the properties. Future work will aim at establishing this correlation. In addition, the substrate scope of the mixed monolayer-protected AuNPs will be extended by introducing other receptor motifs. An attractive option would be the use of Schmuck’s guanidiniocarbonylpyrrole moiety, which should give rise to optical probes for dicarboxylates or peptides [46–48].

## Supporting Information

File 1 Description of the ligand syntheses, NMR, and mass spectra of compounds *rac*-**1**, (*R*)-**1**, and **2**, nanoparticle synthesis, characterization, and details about the binding studies.
