# Supplementary material for: Optical detection of di- and triphosphate anions with mixed monolayer-protected gold nanoparticles containing zinc(II)–dipicolylamine complexes
Source: Beilstein J Org Chem. 2020 Nov 2;16:2687–700. doi: 10.3762/bjoc.16.219 (PMC7607427; doi:10.3762/bjoc.16.219)
Supplement: File 1 — Description of the ligand syntheses, NMR, and mass spectra of compounds rac-1, (R)-1, and 2, nanoparticle synthesis, characterization, and details about the binding studies. [file Beilstein_J_Org_Chem-16-2687-s001.pdf]

BEILSTEIN JOURNAL OF ORGANIC CHEMISTRY

Supporting Information

for

Optical detection of di- and triphosphate anions with mixed monolayer-protected gold nanoparticles containing zinc(II)–dipicolylamine complexes

Lena Reinke, Julia Bartl, Marcus Koch and Stefan Kubik

Beilstein J. Org. Chem. **2020,** *16,* 2687–2700. doi:10.3762/bjoc.16.219

Description of the ligand syntheses, NMR, and mass spectra of compounds *rac*-1, (*R*)-1, and 2, nanoparticle synthesis, characterization, and details about the binding studies

License and Terms: This is a supporting information file under the terms of the Creative Commons Attribution License (https://creativecommons.org/licenses/by/4.0). Please note that the reuse, redistribution and reproduction in particular requires that the authors and source are credited.
The license is subject to the *Beilstein Journal of Organic Chemistry* terms and conditions: (https://www.beilstein-journals.org/bjoc)

Table of contents

Ligand syntheses S2
NMR and mass spectra S9
Nanoparticle synthesis and characterization S15
Binding studies S28
References S44

Ligand syntheses

General details. Solvents were dried according to standard procedures prior to use, if necessary. Starting materials and reagents were commercially available and were used without further purification. Analyses were carried out as follows: ATR-IR, Perkin Elmer Spectrum 100 FT-IR-Spectrometer with Universal ATR Sampling Accessory Unit; Centrifuge, Eppendorf Centrifuge 5702 R; Centrifugal Concentrators, Vivaspin® 15R (MWCO 5000 Da) Sartorius; elemental analysis, Elementar vario Micro cube; ESI-MS, Bruker Esquire 3000 Plus and Esquire 6000; NMR, Bruker AVANCE™ III 400 and 600 (peak assignments were confirmed by using H,H-COSY, HSQC and HMQC spectra, ^{1}H and ^{13}C NMR spectra were referenced to the residual solvent signals ($CDCl_3$: δ^H = 7.26 ppm, δ^C = 77.2 ppm; DMSO-d_6: δ^H = 2.50 ppm, δ^C = 39.52 ppm, MeOD-d_4: δ^H = 3.31 ppm, δ^C = 49.00 ppm); UV–vis spectroscopy, Varian Cary 100; MALDI-TOF-MS, Bruker Ultraflex TOF/TOF; melting points, Müller SPM-X 300; precision balance, Kern ABT 100-5M; preparative chromatography, silica gel 60 A (0.06–0.20 mm) Acros Organics; preparative HPLC, Dionex UltiMate 3000; column, ThermoFisher BetaBasic-18, 250 × 21.2 mm, 5 µm particle size; temperature, 25 °C; flow, 12 mL min^{-1}; eluent, water/acetonitrile for *rac*-**1** and *R*-**1** and 0.1 vol % TFA in water/acetonitrile for **2** with the following gradient: 0–5 min, 10% acetonitrile; 5–31 min, linear increase of 90% acetonitrile, 31–40 min, 90% acetonitrile, 40–41 min, linear decrease to 10% organic; 41–42 min, 10% acetonitrile; size exclusion chromatography, Sephadex® LH-20 and G-10 GE Healthcare; reversed-phase chromatography, RP-8 POLYGOPREP® 60-50 C8 (40–63 µm) Macherey Nagel; TEM, JEOL JEM-2100 LaB6 Transmission Electron Microscope equipped with a Gatan Orius SC1000 CCD camera; UV–vis, Varian Cary 100.

The following abbreviations are used: AuNP, gold nanoparticle; TBTU, *O*-(1*H*-benzotriazol-1-yl)-*N*,*N*,*N'*,*N'*-tetramethyluronium tetrafluoroborate; EDC, *N*-ethyl-*N'*-(3-dimethylaminopropyl)carbo-diimide hydrochloride.

2-(2-(2-Methoxyethoxy)ethoxy)ethyl 4-methylbenzenesulfonate.[1]

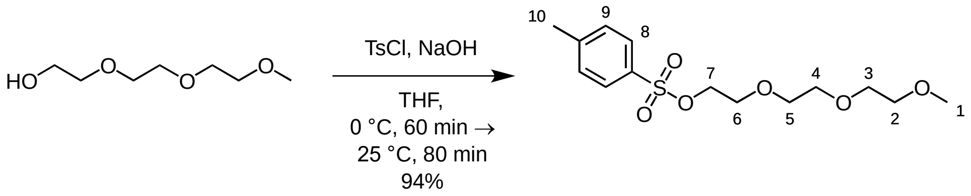

Triethylene glycol monomethylether (8.21 g, 50.0 mmol) was dissolved in THF (15 mL) and the resulting solution cooled to 0 °C. A solution of NaOH (3.86 g, 96.5 mmol) in water (16 mL) was added dropwise under stirring followed by a solution of 4-toluenesulfonyl chloride (12.4 g, 65.0 mmol) in THF (18 mL). The reaction mixture was stirred for 60 min at 0 °C and further 80 min at 25 °C. Afterwards, the solution was diluted with diethyl ether (125 mL) and 1 mol/L NaOH (40 mL) and the aqueous phase was separated. The organic phase was washed with water (2 × 50 mL) dried over $MgSO_4$ and concentrated in vacuo. Yield: 15.0 g (47.1 mmol, 94%) colourless oil; ^1H NMR (400 MHz, 25 °C, $CDCl_3$): δ = 7.78 (m, 2H, H^8), 7.33 (m, 2H, H^9), 4.15 (t, 2H, ^3J = 4.0 Hz, H^7), 3.67 (t, 2H, ^3J = 4.0 Hz, H^6), 3.51-3.61 (m, 8H, H^2, H^3, H^4, H^5), 3.36 (s, 3H, H^1), 2.44 (s, 3H, H^{10}) ppm.

1-Azido-2-(2-(2-methoxyethoxy)ethoxy)ethane.[1]

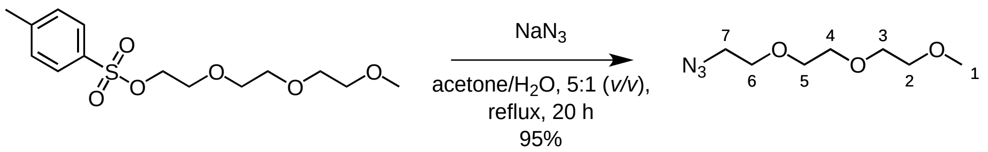

2-(2-(2-Methoxyethoxy)ethoxy)ethyl 4-toluenesulfonate (7.96 g, 25.0 mmol) and sodium azide (2.44 g, 37.5 mmol) were dissolved in acetone (30 mL) and water (6 mL). The mixture was heated to reflux for 20 h. Afterwards, the acetone was removed in vacuo and the remaining solution was diluted with water (20 mL). The reaction mixture was extracted with diethyl ether (4 × 20 mL), the combined organic phases were dried over $MgSO_4$, and concentrated in vacuo. Yield: 4.51 g (23.8 mmol, 95%) colourless oil; ^1H NMR (400 MHz, 25 °C, $CDCl_3$): δ = 3.64-3.68 (m, 8H, H^2, H^3, H^4, H^5), 3.53-3.57 (m, 2H, H^6), 3.37-3.39 (m, 5H, H^1, H^7) ppm.

2-(2-(2-Methoxyethoxy)ethoxy)ethanamine hydrochloride.

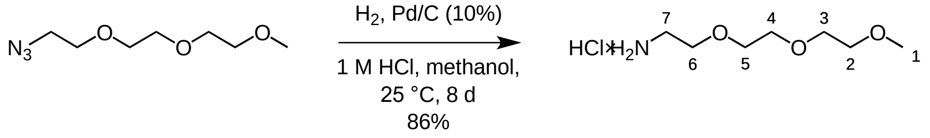

1-Azido-2-(2-(2-methoxyethoxy)ethoxy)ethane (5.67 g, 30.0 mmol) was dissolved in methanol (100 mL). A suspension of Pd/C (570 mg, 10 wt %) in water (5 mL) and 1 mol/L aqueous HCl (33.0 mL, 33.0 mmol) were added. The reaction mixture was stirred for 8 d at 25 °C under an

atmosphere of hydrogen. Afterwards, the catalyst was removed by filtering the reaction mixture filtered through celite and the filtrate was evaporated to dryness. Yield: 5.17 g (25.9 mmol, 86%) pale yellow oil; ^{1}H NMR (400 MHz, 25 °C, $CDCl_3$): δ = 6.31 (s, 2H, NH), 3.77 (t, 2H, ^{3}J = 4.0 Hz, H^2), 3.61-3.68 (m, 6H, H^3, H^4, H^5), 3.35-3.39 (m, 5H, H^1, H^6), 3.05-3.15 (m, 2H, H^7) ppm.

Ligand 1

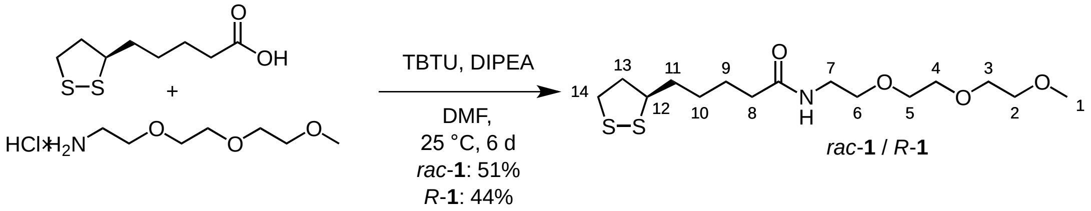

Lipoic acid (4.13 g, 20.0 mmol) (*rac*-lipoic acid for the synthesis of *rac*-**1** or (*R*)-lipoic acid for the synthesis of *R*-**1**) and 1-amino-2-(2-(2-methoxyethoxy)ethoxy)ethane hydrochloride (5.17 g, 25.9 mmol) were dissolved in DMF (50 mL). TBTU (8.31 g, 25.9 mmol) and DIPEA (13.2 mL, 77.7 mmol) were added, and the reaction mixture was stirred for 6 d at 25 °C. Afterwards, the solvent was removed in vacuo and the residue was purified chromatographically over silica (ethyl acetate/petroleum ether 2:1 (v/v) → acetone). The thus obtained crude product was further purified by preparative HPLC. The pure fractions were collected and evaporated to dryness.

Ligand *rac*-**1**. Yield: 3.59 g (10.2 mmol, 51%) pale yellow oil; specific rotation, $[\alpha]_D^{25}$ = 0 (*c* = 1, methanol), ^{1}H NMR (400 MHz; $CDCl_3$): δ 6.40 (s, 1H, NH), 3.67-3.61 (m, 11H, H^2, H^3, H^4, H^5, H^6, H^{12}), 3.47-3.43 (m, 2H, H^7), 3.38 (s, 3H, H^1), 3.20-3.07 (m, 2H, H^{14}), 2.49-2.41 (m, 1H, H^{13}), 2.22 (t, ^{3}J = 7.5 Hz, 2H, H^8), 1.94-1.86 (m, 1H, H^{13}), 1.74-1.59 (m, 4H, H^9, H^{11}), 1.52-1.39 (m, 2H, H^{10}) ppm; ^{13}C NMR (101 MHz, 25 °C, $CDCl_3$): δ = 173.8 (CO), 72.0 (C^2), 72.6 + 70.5 + 70.3 + 69.8 (C^3 + C^4 + C^5 + C^6), 59.1 (C^1), 56.5 (C^7), 40.4 (C^{12}), 39.5 (C^{14}), 38.6 (C^{13}), 36.3 (C^8), 34.7 (C^{11}), 29.0 (C^9), 25.6 (C^{10}) ppm; MS (MALDI-TOF) *m/z* (%): 352.1 $[M+H]^+$ (100%), 374.1 $[M+Na]^+$ (50%), 390.1 $[M+K]^+$ (27%); IR (ATR): 3308 (w), 2922 (m), 2863 (m), 1645 (s), 1095 (s), 848 (w) cm^{-1}; elemental analysis (%) calcd for $C_{15}H_{29}NO_4S_2\cdot0.5H_2O$: C, 49.97%, H, 8.39%, N, 3.88%, S, 17.79%, found: C, 49.98%, H, 8.35%, N, 3.92%, S, 17.55 %.

Ligand *R*-**1**: Yield: 3.11 g (8.84 mmol, 44%) pale yellow oil; specific rotation, $[\alpha]_D^{25}$ = 57.2 (*c* = 1, methanol), ^{1}H NMR (400 MHz; $CDCl_3$): δ 6.30 (s, 1H, NH), 3.64-3.52 (m, 11H, H^2, H^3, H^4, H^5, H^6,

H^{12}), 3.46-3.39 (m, 2H, H^7), 3.36 (s, 3H, H^1), 3.19-3.06 (m, 2H, H^{14}), 2.49-2.39 (m, 1H, H^{13}), 2.17 (t, ^3J = 7.5 Hz, 2H, H^8), 1.94-1.83 (m, 1H, H^{13}), 1.74-1.57 (m, 4H, H^9, H^{11}), 1.52-1.37 (m, 2H, H^{10}) ppm; ^{13}C NMR (101 MHz, 25 °C, $CDCl_3$): δ = 173.0 (CO), 72.0 (C^2), 70.6 + 70.5 + 70.2 + 70.0 (C^3 + C^4 + C^5 + C^6), 59.1 (C^1), 56.5 (C^7), 40.3 (C^{12}), 39.2 (C^{14}), 38.5 (C^{13}), 36.4 (C^8), 34.7 (C^{11}), 29.0 (C^9), 25.5 (C^{10}) ppm; MS (MALDI-TOF) *m/z* (%): 352.3 $[M+H]^+$ (100%), 374.3 $[M+Na]^+$ (7%); IR (ATR): 3311 (w), 2923 (m), 2863 (m), 1646 (s), 1095 (s), 850 (w) cm^{-1}; elemental analysis (%) calcd for $C_{15}H_{29}NO_4S_2 \cdot 0.5H_2O$: C, 49.35%, H, 8.42%, N, 3.84%, S, 17.57%, found: C, 49.24%, H, 8.09%, N, 3.68%, S, 17.39 %.

Bis(2-pyridylmethyl)amine.[2]

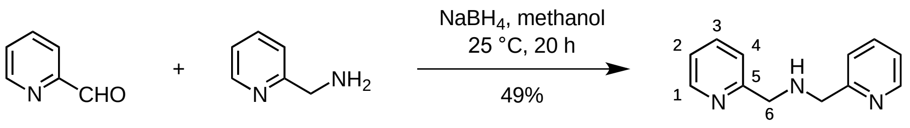

To a solution of 2-(aminomethyl)pyridine (5.40 g, 5.50 mL, 50.0 mmol) in methanol (15 mL) was slowly added a solution of pyridine-2-carboxaldehyde (5.35 g, 4.75 mL, 50.0 mmol) in methanol (15 mL). The yellow reaction mixture was stirred for 1 h at room temperature. Afterwards, $NaBH_4$ (1.89 g, 50.0 mmol) was added in portions at 0 °C. Once the addition was completed, the solution was stirred for 20 h at ambient temperature. The reaction mixture was poured onto ice (50 g) and the mixture was adjusted to a pH of 4 with HCl conc. The solvent mixture was evaporated and the crude product dissolved in H_2O (25 mL). The solution was washed with dichloromethane (6 × 30 mL). The aqueous layer was treated with saturated aqueous Na_2CO_3 to adjust the pH to 10, extracted with dichloromethane (3 × 40 mL), and dried over magnesium sulfate. The solvent was removed in vacuo to obtain a yellow oil. Yield: 4.87 g (24.4 mmol, 49%); ^{1}H NMR (400 MHz, $CDCl_3$): δ = 8.50 (d, 2H, ^3J = 4.8 Hz, H^1), 7.57 (td, 2H, ^3J = 7.7 Hz, ^4J = 1.6 Hz, H^3), 7.30 (d, 2H, ^3J = 7.8 Hz, H^4), 7.09 (dd, 2H, ^3J = 7.3 Hz, ^3J = 5.1 Hz, H^2), 3.92 (s, 4H, H^6), 2.70 (s, 1H, NH) ppm; ^{13}C NMR (101 MHz, $CDCl_3$): δ = 159.7 (C^5), 149.3 (C^1), 136.4 (C^3), 122.3 (C^4), 121.9 (C^2), 54.8 (C^6) ppm; MALDI-TOF MS *m/z* (%): 199.8 (100) $[M+H]^+$.

2-(12-Bromododecyl)isoindoline-1,3-dione.[3]

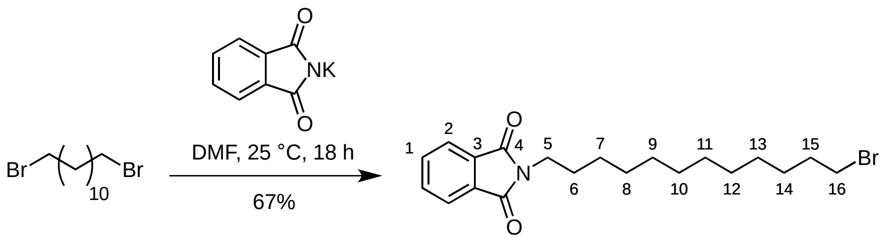

To a suspension of potassium phthalimide (3.94 g, 21.3 mmol) in dimethylformamide (40 mL) was added a solution of 1,12-dibromododecane (13.9 g, 42.5 mmol) in dimethylformamide (40 mL). The reaction mixture was stirred for 18 h at ambient temperature. The solid was removed by filtration and the solvent evaporated. The crude product was purified by column chromatography (SiO_2, hexane → ethyl acetate/hexane 1:4 (v/v)) to afford the product as colorless solid. Yield: 5.61 g (14.2 mmol, 67%); ^{1}H NMR (400 MHz, $CDCl_3$): δ = 7.84 (m, 2H, H^2), 7.70 (m, 2H, H^1), 3.67 (t, 2H, ^3J = 7.3 Hz, H^5), 3.40 (t, 2H, ^3J = 6.9 Hz, H^{16}), 1.84 (quin, 2H, ^3J = 7.1 Hz, H^6), 1.66 (quin, 2H, ^3J = 7.2 Hz, H^{15}), 1.40 (quin, 2H, ^3J = 7.2 Hz, H^7), 1.32-1.25 (m, 14H, H^8-H^{14}) ppm; ^{13}C NMR (101 MHz, $CDCl_3$): δ = 168.6 (C^4), 134.0 (C^1), 132.3 (C^3), 123.3 (C^2), 38.2 (C^5), 34.2 (C^{16}), 33.0, 29.62, 29.60, 29.57, 29.53, 29.3, 28.9, 28.3, 27.0 (C^6-C^{15}) ppm; elemental analysis (%) calcd for $C_{20}H_{28}NO_2$ (M.W. 394.35): C 60.91, H 7.16, N 3.55; found C 60.94, H 7.16, N 3.49.

2-(12-(Bis(pyridin-2-ylmethyl)amino)dodecyl)isoindoline-1,3-dione.

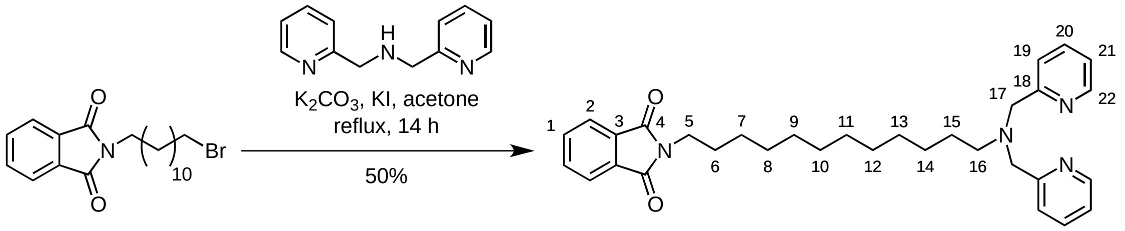

Bis(2-pyridylmethyl)amine (2.79 g, 14.0 mmol) and 2-(12-bromododecyl)isoindolin-1,3-dione (5.53 g, 14.0 mmol) were each dissolved in acetone (30 mL). These solutions were added to a suspension of potassium carbonate (5.80 g, 42.0 mmol) and potassium iodide (230 mg, 1.40 mmol) in acetone (30 mL). The reaction mixture was refluxed for 14 h. Afterwards, the solid was separated by filtration and the filtrate was evaporated. The resulting orange oil was purified by column chromatography (SiO_2, acetone) and then subjected to preparative HPLC. Yield: 3.61 g (7.04 mmol, 50%); ^{1}H NMR (400 MHz, $CDCl_3$): δ = 8.69 (d, 2H, ^3J = 5.0 Hz, H^{22}), 7.95 (td, 2H, ^3J = 7.8 Hz, ^4J

= 1.4 Hz, H[21]), 7.82-7.80 (m, 2H, H[2]), 7.71-7.67 (m, 4H, H1, H[19]), 7.49-7.46 (m, 2H, H[21]), 4.49 (s, 4H, H[17]), 3.64 (t, 2H, ^3J = 7.3 Hz, H[5]), 3.04 (t, 2H, ^3J = 8.0 Hz, H[16]), 1.73-1.60 (m, 4H, H[6], H[15]), 1.29-1.18 (m, 16H, H[7]-H[14]) ppm; ^{13}C NMR (101 MHz, $CDCl_3$): δ = 168.6 (C[4]), 161.2 (TFA), 151.3 (C[18]), 147.3 (C[22]), 140.5 (C[20]), 134.0 (C[3]), 132.2 (C[1]), 125.7 (C[19]), 124.8 (C[2]), 123.2 (C[22]), 56.9 (C[17]), 54.7 (C[16]), 38.2 (C[5]), 29.50, 29.48, 29.44, 29.32, 29.21, 29.03, 28.66, 26.9, 26.6, 25.0 (C[6]-C[15]).ppm; MALDI-TOF MS *m/z* (%): 513 (100) $[M+H]^+$; elemental analysis (%) calcd for $C_{32}H_{40}N_4O_2$·1.5 H_2O·1.5 CF_3COOH (M.W. 710.75): C 59.19, H 6.31, N 7.88 found C 59.22, H 6.11, N 7.83.

***N*[1],*N*[1]-Bis(pyridin-2-ylmethyl)dodecane-1,12-diamine**.

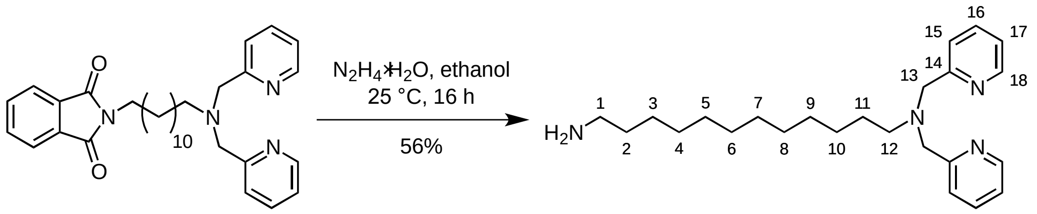

2-(12-(Bis(pyridin-2-ylmethyl)amino)dodecyl)isoindoline-1,3-dione (3.44 g, 6.71 mmol) was dissolved in ethanol (100 mL). This solution was added dropwise to a solution of hydrazine monohydrate (3.36 g, 3.00 mL, 67.1 mmol) in ethanol (100 mL). The reaction mixture was stirred for 16 h at ambient temperature. Afterwards, the suspension was filtered and the solvent was evaporated. The crude product was dissolved in dichloromethane (50 mL) and washed with 1 mol/L aqueous NaOH (1 × 50 mL) and water (1 × 50 mL). The organic layer was dried over $MgSO_4$ and the solvent was evaporated. Yield: 1.44 g (3.76 mmol, 56%) yellow oil; ^{1}H NMR (400 MHz, DMSO-d_6): δ = 8.45 (m, 2H, H[18]), 7.75 (td, 2H, ^3J = 7.7 Hz, ^4J = 1.7 Hz, H[16]), 7.50 (d, 2H, ^3J = 7.8 Hz, H[15]), 7.23 (dd, 2H, ^3J = 6.7 Hz, ^3J = 5.1 Hz, H[17]), 3.70 (s, 4H, H[13]), 2.48-2.46 (m, 2H, H[1]), 2.41 (t, 2H, ^3J = 7.1 Hz, H[12]), 1.47-1.40 (m, 2H, H[2]), 1.31-1.27 (m, 2H, H[11]), 1.20-1.16 (m, 16H, H[3]-H[10]) ppm; ^{13}C NMR (101 MHz, DMSO-d_6): δ = 159.7 (C[14]), 148.7 (C[18]), 136.5 (C[16]), 122.6 (C[15]), 122.1 (C[17]), 59.9 (C[13]), 53.5 (C[1]), 41.8 (C[12]), 33.4 (C[2]), 29.2, 29.1, 29.0, 29.0, 28.8, 26.6, 26.5, 26.5 (C[3]-C[11]) ppm; MALDI-TOF MS *m/z* (%): 245 (9) $[C_{15}H_{18}NO_2+H]^+$, 292 (75) $[C_{17}H_{23}N_3+Na]^+$, 383 (100) $[M+H]^+$, 405 (61) $[M+Na]^+$; elemental analysis (%) calcd for $C_{24}H_{38}N_4$·0.5 H_2O (M.W. 391.60): C 73.61, H 10.04, N 14.31 found C 73.81, H 9.96, N 14.20.

Ligand 2.

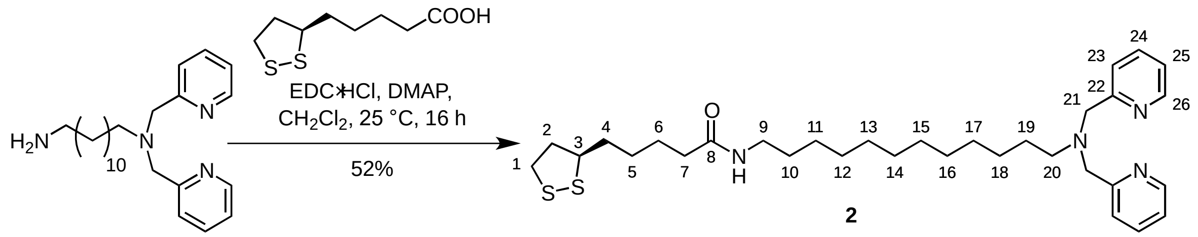

Lipoic acid (521 mg, 2.53 mmol) was dissolved in dichloromethane (10 mL). This solution was added dropwise to a solution of EDC·HCl (533 mg, 2.78 mmol) and 4-dimethylaminopyridine (30.9 mg, 0.25 mmol) in dichloromethane (15 mL). A solution of N^1,N^1-bis(pyridin-2-ylmethyl)dodecane-1,12-diamine (1.06 g, 2.78 mmol) in dichloromethane (10 mL) was added dropwise. The reaction mixture was stirred for 16 h at ambient temperature. Afterwards, water (50 mL) was added and the mixture was stirred for 5 min. The layers were separated and the procedure was repeated once more. The solvent was removed and the crude product was purified by reversed-phase column chromatography (RP-8, gradient 0.1 vol % aqueous trifluoroacetic acid/acetonitrile, 9:1 → 4:1 → 1:1 (v/v)). The solvent was removed and the residue subjected to preparative HPLC. The solvent was removed, the yellow oil was dissolved in dichloromethane (50 mL) and washed with saturated aqueous $NaHCO_3$ (3 × 50 mL). The organic layer was dried over $MgSO_4$ and the solvent was evaporated to dryness. Yield: 781 mg, (1.32 mmol, 52%) yellow oil; ^{1}H NMR (400 MHz, $CDCl_3$): δ = 8.45 (d, 2H, ^{3}J = 4.9 Hz, H^{26}), 7.60 (td, 2H, ^{3}J = 7.6 Hz, ^{4}J = 1.8 Hz, H^{24}), 7.50 (ddd, 2H, ^{3}J = 7.2 Hz, ^{3}J = 4.9 Hz, ^{4}J = 0.9 Hz, H^{23}), 7.15-7.12 (m, 2H, H^{25}), 5.80 (t, 1H, ^{3}J = 5.1 Hz, NH), 3.75 (s, 4H, H^{21}), 3.54-3.47 (m, 1H, H^{3}), 3.17 (q, 2H, ^{3}J = 6.7 Hz, H^{9}), 3.14-3.01 (m, 2H, H^{1}), 2.47 (t, 2H, ^{3}J = 7.4 Hz, H^{20}), 2.43-2.35 (m, 1H, H^{2}), 2.11 (t, 2H, ^{3}J = 7.3 Hz, H^{7}), 1.88-1.80 (m, 1H, H^{2}), 1.70-1.53 (m, 4H, H^{4}, H^{6}), 1.49-1.35 (m, 6H, H^{10}, H^{11}, H^{19}), 1.21-1.16 (m, 16H, H^{5}, H^{12}-H^{18}) ppm; ^{13}C NMR (101 MHz, $CDCl_3$): δ = 172.7 (C^{8}), 160.2 (C^{22}), 148.9 (C^{26}), 136.4 (C^{24}), 122.8 (C^{23}), 121.9 (C^{25}), 60.5 (C^{21}), 56.5 (C^{20}), 54.5 (C^{3}), 40.3 (C^{2}), 39.6 (C^{9}), 36.5 (C^{1}), 34.7 (C^{7}), 29.7, 29.5, 29.4, 29.3, 27.3, 27.0, 26.9, 25.5 (C^{4}-C^{6}, C^{10}-C^{19}) ppm; MALDI-TOF MS *m/z* (%): 571.2 (100) [$M+H^{+}$]; elemental analysis (%) calcd for $C_{32}H_{50}N_4OS_2$·0.8 H_2O (M.W. 585.31): C 65.67, H 8.89, N 9.57, S 10.96; found C 65.88, H 8.71, N 9.77, S 10.39.

NMR and mass spectra of *rac*-1

^{1}H NMR (400 MHz, $CDCl_3$)

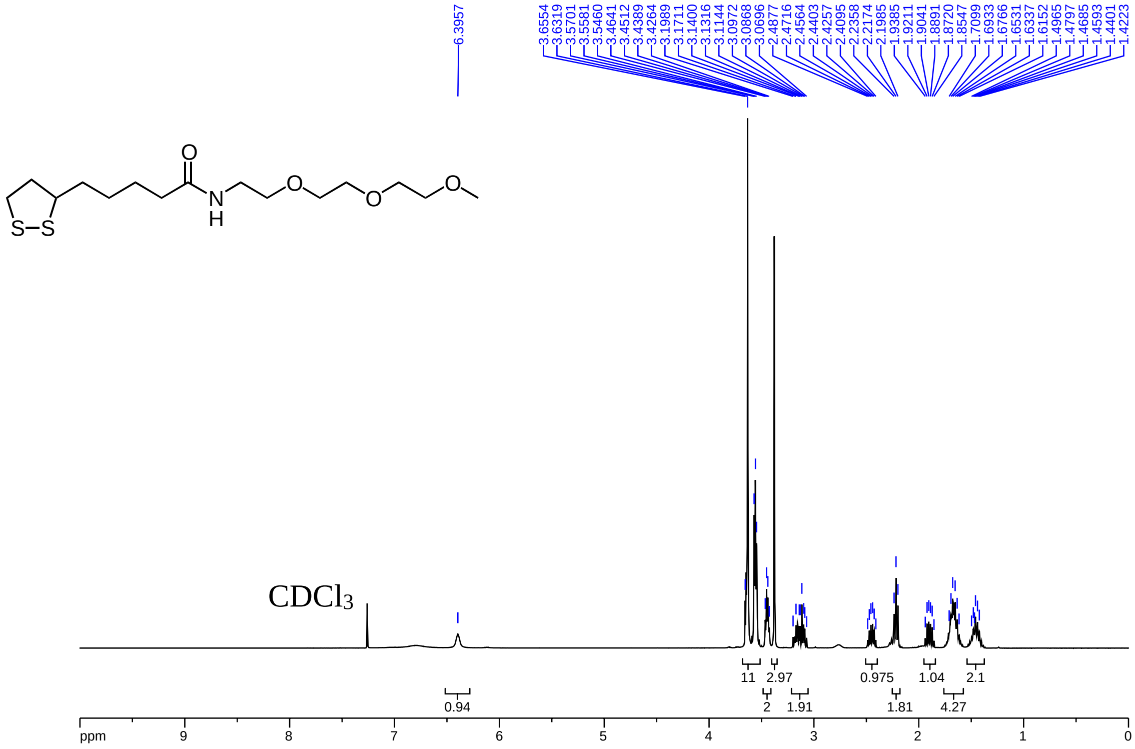

^{13}C NMR (101 MHz, $CDCl_3$)

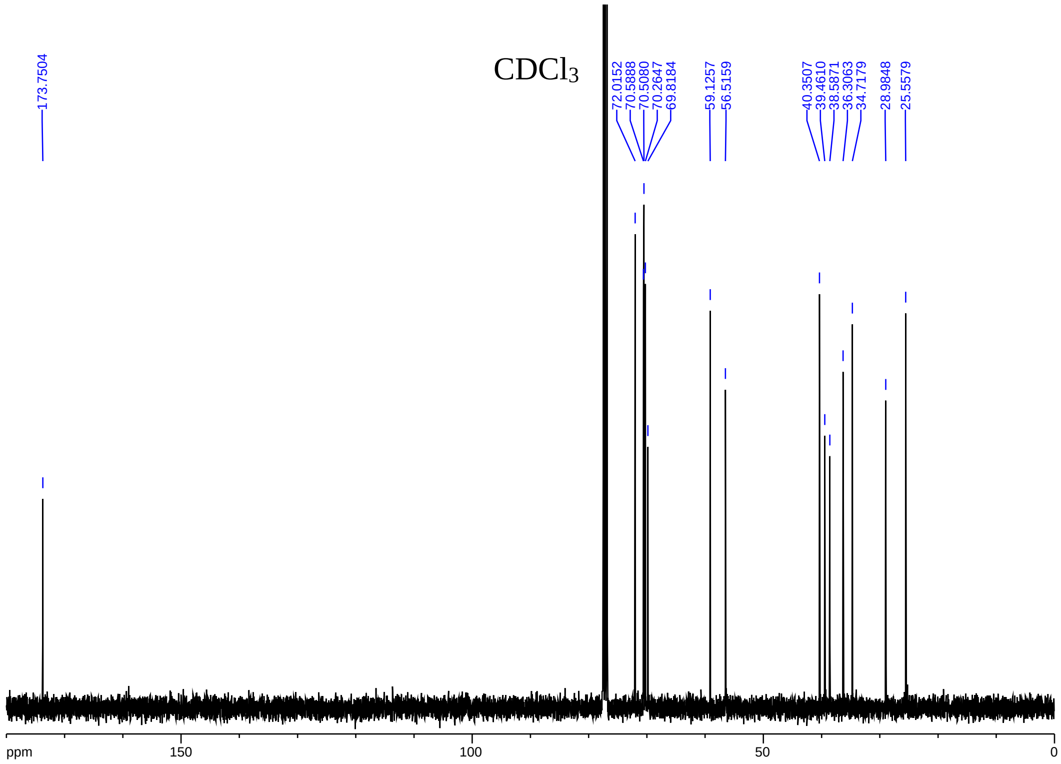

MALDI-TOF MS (positive mode)

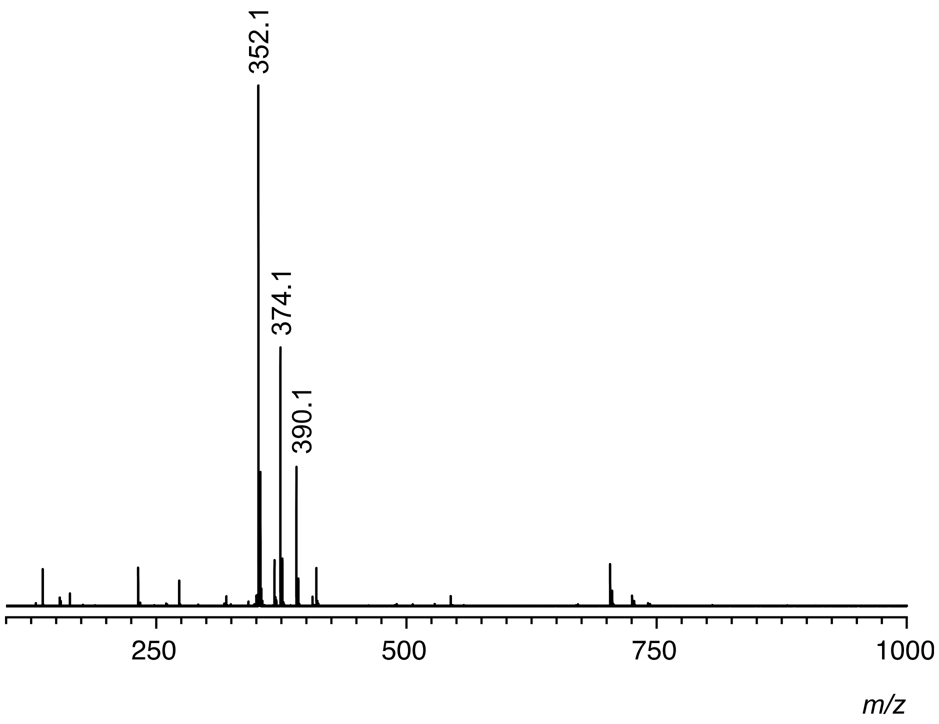

		m/z calcd.	*m/z exp.*
$[M+H]^+$	$C_{15}H_{29}NO_4S_2 + H^+$	352.2	352.1
$[M+Na]^+$	$C_{15}H_{29}NO_4S_2 + Na^+$	374.1	374.1
$[M+K]^+$	$C_{15}H_{29}NO_4S_2 + K^+$	390.1	374.1

NMR and mass spectra of *R*-1

^{1}H NMR (400 MHz, $CDCl_3$)

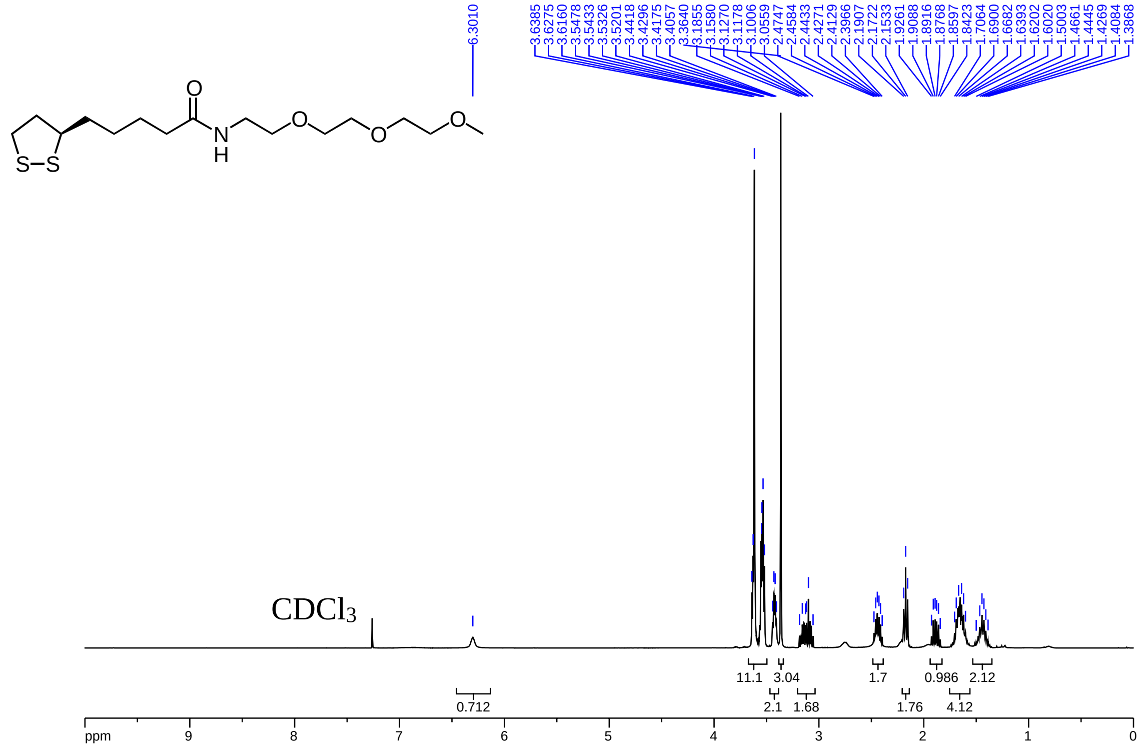

^{13}C NMR (101 MHz, $CDCl_3$)

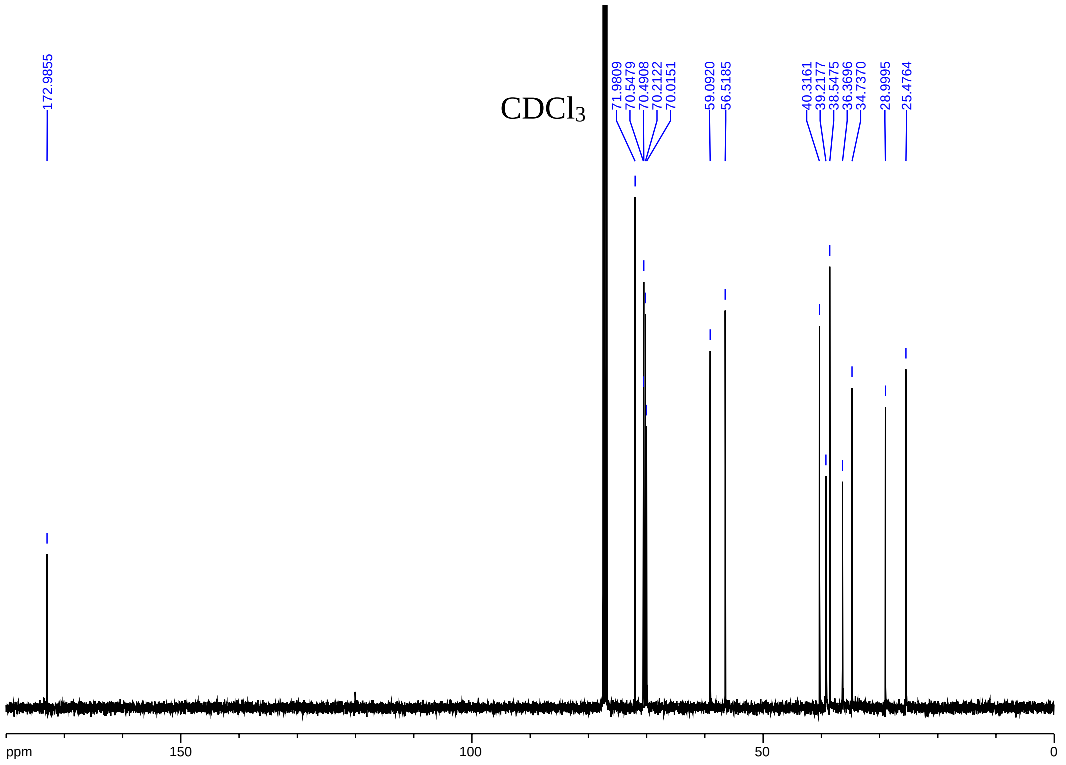

MALDI-TOF MS (positive mode)

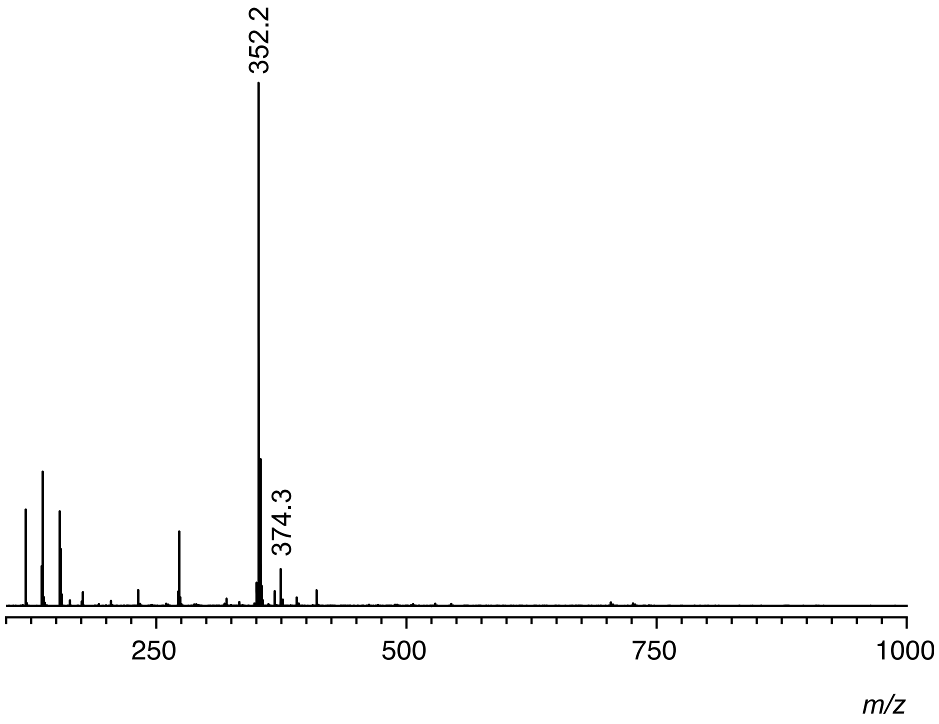

		m/z calcd.	*m/z exp.*
$[M+H]^+$	$C_{15}H_{29}NO_4S_2 + H^+$	352.2	352.3
$[M+Na]^+$	$C_{15}H_{29}NO_4S_2 + Na^+$	374.1	374.3

NMR and mass spectra of 2

^{1}H NMR ($CDCl_3$, 400 MHz)

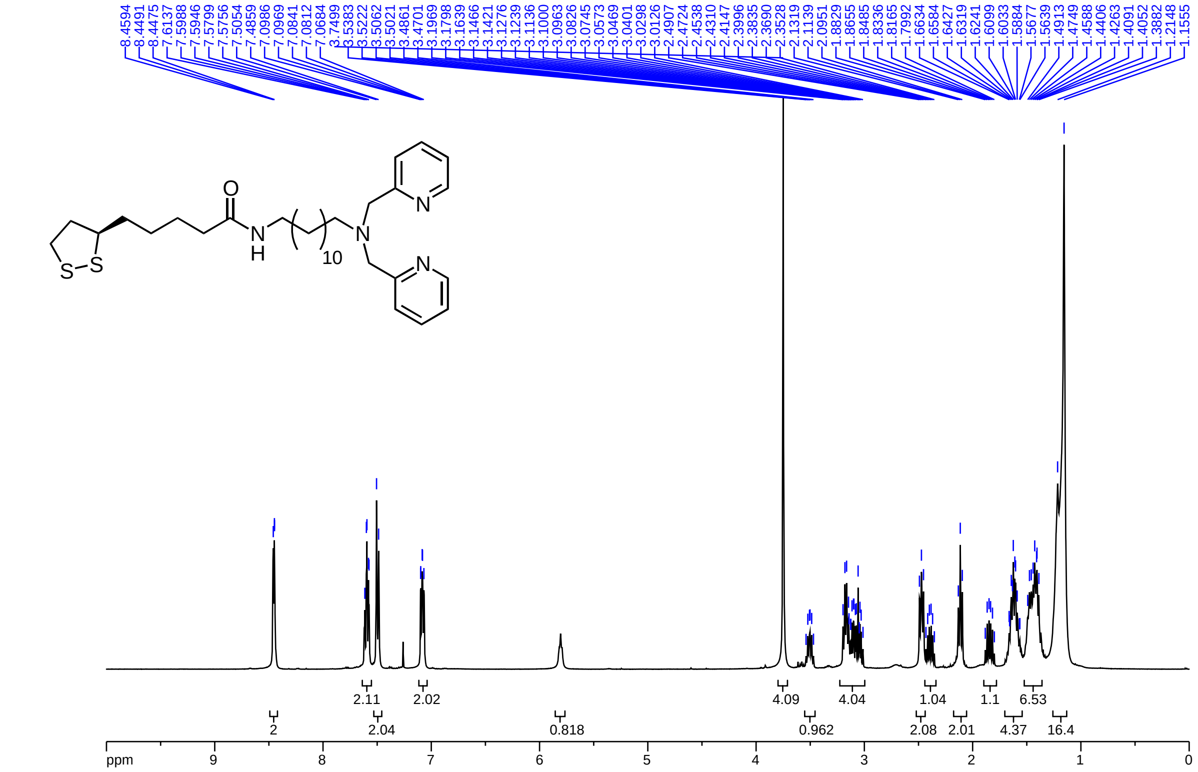

^{13}C NMR ($CDCl_3$, 101 MHz)

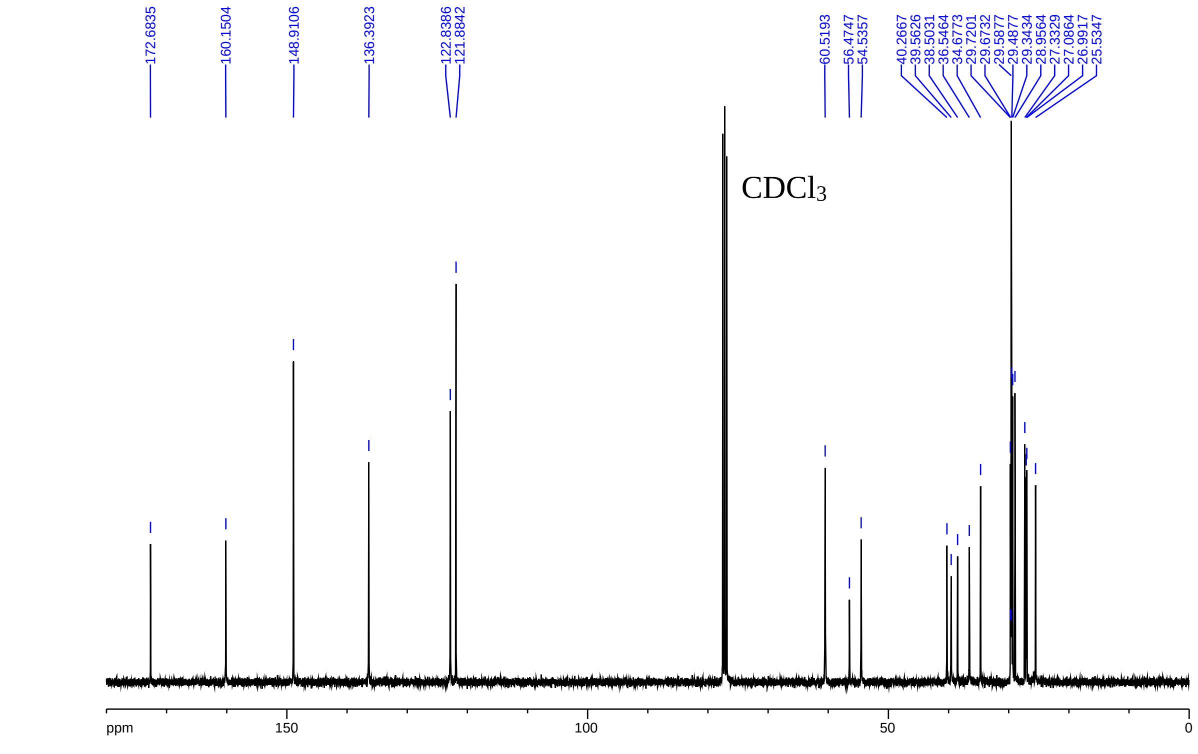

MALDI-TOF MS (positive mode)

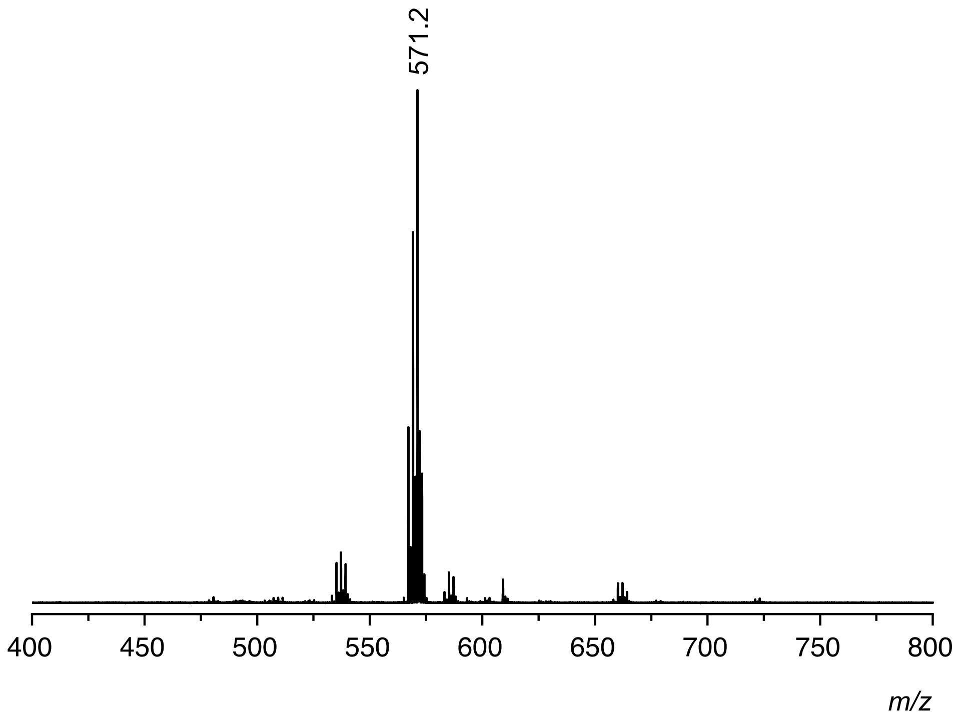

		m/z calcd.	*m/z* exp.
$[M+H]^+$	$C_{32}H_{50}N_4OS_2 + H^+$	571.4	571.2

Nanoparticle synthesis and characterization

Analytical methods

^{1}H NMR spectroscopy. The ^{1}H NMR spectroscopic measurements were performed on a Bruker Avance™ III 400 spectrometer at 400 MHz and 22 °C. CD_3OD and D_2O were used as purchased.

UV–vis spectroscopy. The UV–vis measurements were performed by using a Varian Cary 100 spectrometer in semi-micro PMMA disposable cuvettes. All measurements were performed at 22 °C by using HPLC grade water as the solvent. The spectra were recorded between 350 and 800 nm. The AuNPs and the salts were weighed by using an analytical precision balance. Blank measurements were performed with water or water/methanol 1:2 (v/v).

Transmission electron microscopy. A droplet of an aqueous AuNP solution was placed on a holey carbon grid (Plano S147-4) and dried under ambient conditions. A JEOL JEM-2100 LaB6 Transmission Electron Microscope (TEM) equipped with a Gatan Orius SC1000 CCD camera was used for bright-field imaging at 200 kV accelerating voltage. The images have a size of 1024 × 1024 pixels (acquisition time 0.5 s). The average diameters of the AuNPs were determined by processing the images with ImageJ followed by statistical analysis with MS Excel.

Qualitative binding studies. All experiments were performed by using HPLC grade water and the nanoparticles and salts weighed by using an analytical precision balance.

Syntheses

Citrate-stabilized AuNPs (NP^{cit}).[4] Trisodium citrate dihydrate (484 mg, 1.65 mmol) was dissolved in water (250 mL) and the resulting solution was refluxed for 15 min. Meanwhile, a solution of $HAuCl_4$ (44.8 mg, 132 µmol) in water (1 mL) was also heated to 100 °C and then added quickly to the refluxing citrate solution. The reaction mixture was refluxed for additional 20 min and then allowed to cool to 25 °C. Prior to functionalization, an aliquot of the resulting nanoparticles solution was dialyzed against water for 24 h at 25 °C.

TEG-stabilized AuNPs NP$^{rac\text{-}\mathbf{1}}$. To the dialyzed NP^{cit} stock solution (20 mL), a solution of *rac*-**1** (1.00 mL, 100 µmol, 0.1 mol/L in methanol) was added and the reaction mixture was stirred for 24 h at 25 °C. The solvent was removed in vacuo, the residue was redissolved in water (1.0 mL), and the AuNPs purified first by size exclusion chromatography (Sephadex® G-10, water/methanol 1:1 (v/v)) and then by membrane filtration. For this, the AuNPs were dissolved in water/methanol 1:1 (v/v), the solution subjected to centrifugal concentrators with a MWCO membrane (5000 Da) and filtered off by centrifugation (3000 rpm, 12 °C). The membrane filtration was repeated three times. The resulting AuNPs were collected and dried. Yield: 4.9 mg.

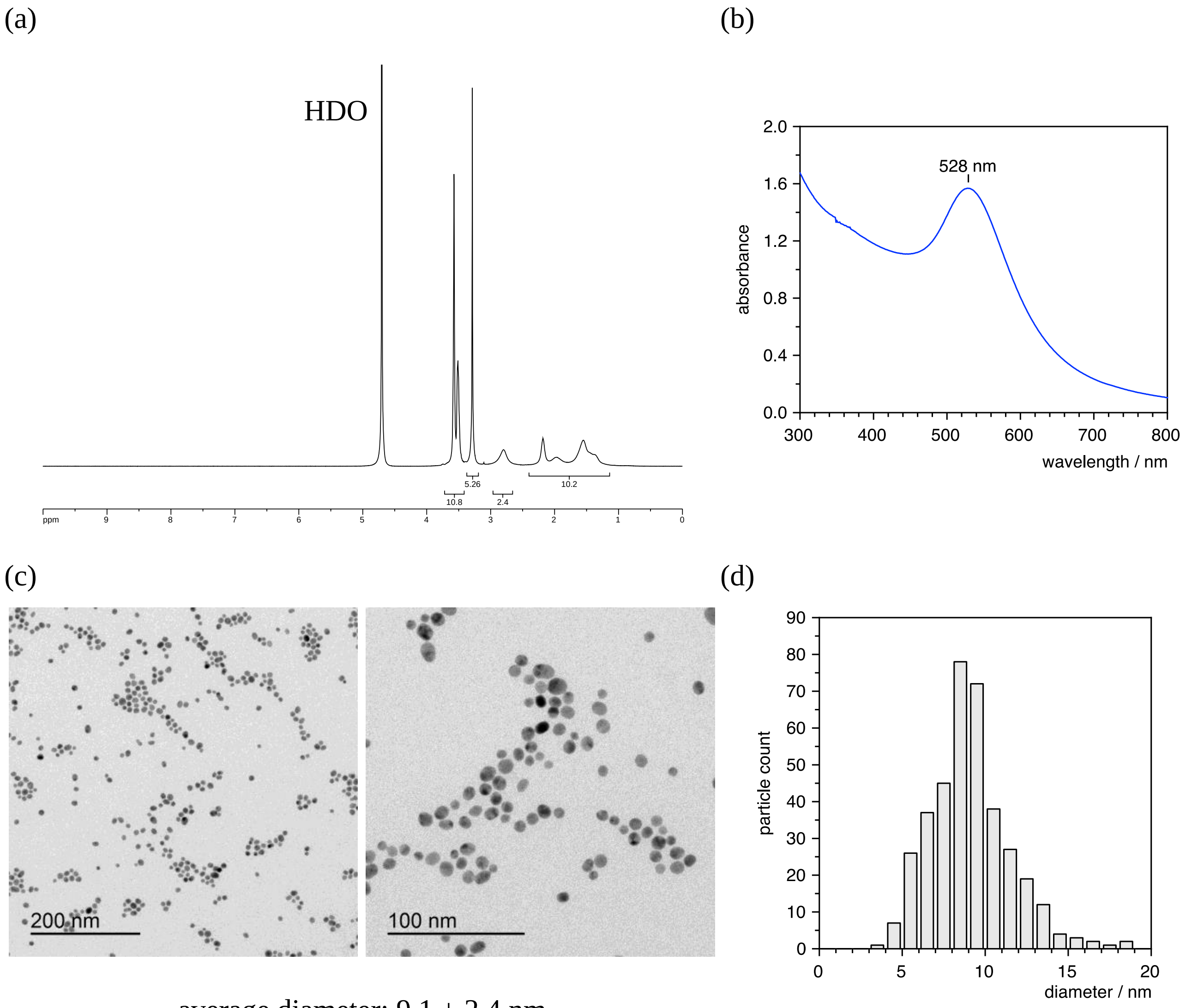

average diameter: 9.1 ± 2.4 nm

Figure S1: ^{1}H NMR spectrum in D_2O (a) and UV–vis spectrum (b) of NP$^{rac\text{-}\mathbf{1}}$ in water, and TEM image (c) together with the histogram illustrating the size distribution of the nanoparticles derived from the TEM image (d).

TEG-stabilized AuNPs NP$^{R\text{-}\mathbf{1}}$. These nanoparticles were prepared in a similar fashion as NP$^{R\text{-}\mathbf{1}}$ by using *R*-**1** instead of *rac*-**1**. Yield: 4.8 mg.

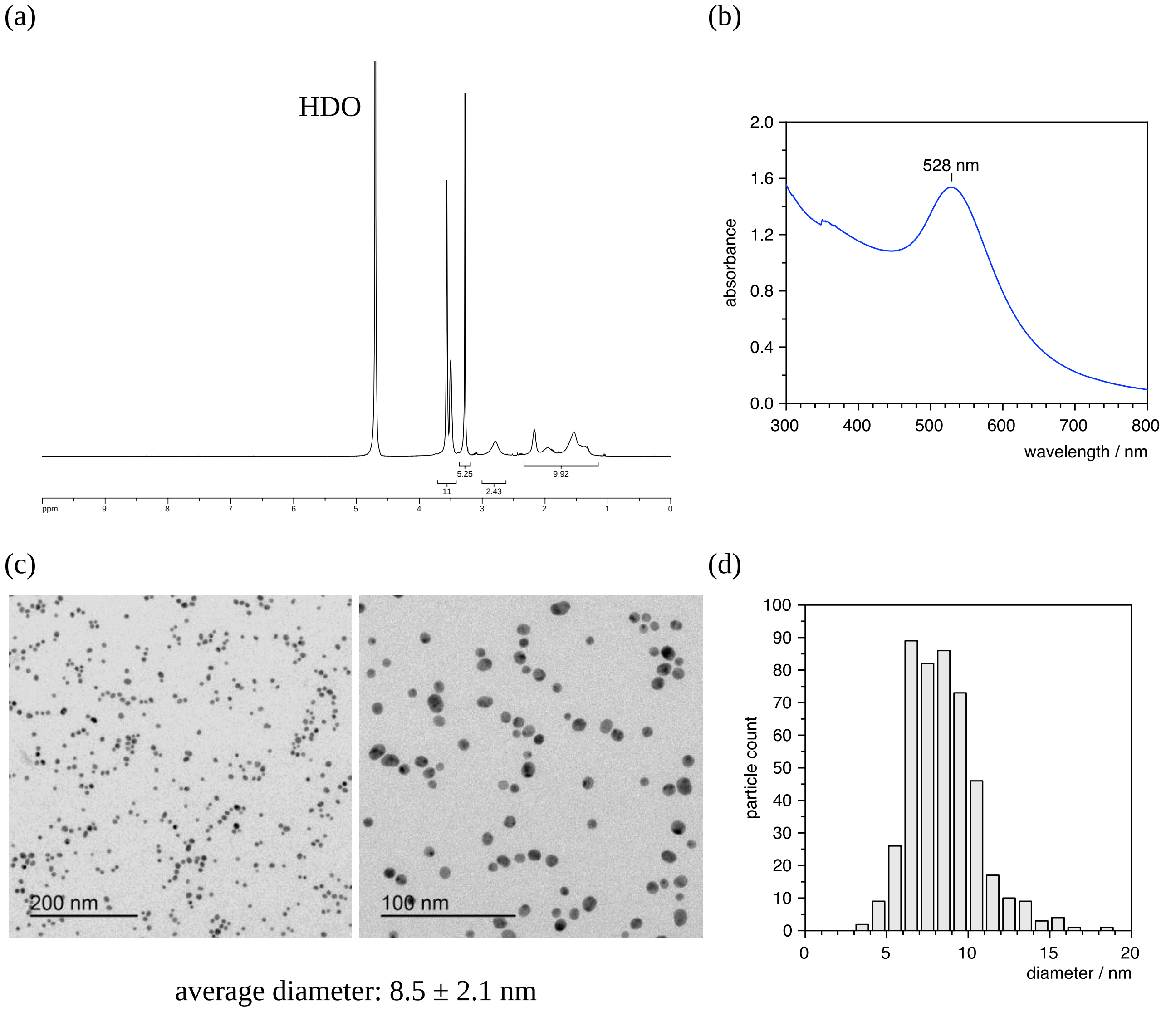

Figure S2: ^{1}H NMR spectrum in D_2O (a) and UV–vis spectrum (b) of NP$^{R\text{-}\mathbf{1}}$ in water, and TEM image (c) together with the histogram illustrating the size distribution of the nanoparticles derived from the TEM image (d).

Determination of nanoparticle composition by iodine decomposition. A known amount of a nanoparticle, weighed with a precision balance, was dissolved in an NMR tube in D_2O (500 µL). A solution of iodine in CD_3OD (0.1 mol/L, 100 µL) was added and the resulting mixture treated for 2 h in an ultrasound bath at 40 °C. A stock solution of 2,4,6-trimethoxy-1,3,5-triazine in CD_3OD (0.1 mol/L, 10 µL) was added and the ^{1}H NMR spectrum recorded.

For the evaluation, the integral of the 2,4,6-trimethoxy-1,3,5-triazine signal was set to 9, the integrals

of the ligand signals were determined and the sum divided by the number of absorbing protons. Relating the resulting value with the known amount of 2,4,6-trimethoxy-1,3,5-triazine in the solution (1 µmol) gave the molar amount of the ligand, which allowed calculating the amount of ligand in mg. The amount of gold in the sample accordingly amounted to the difference between the total amount of nanoparticle and the amount of ligand. The following ^{1}H NMR spectrum, corresponding to entry 7 in Table **S1**, is an example of such a measurement.

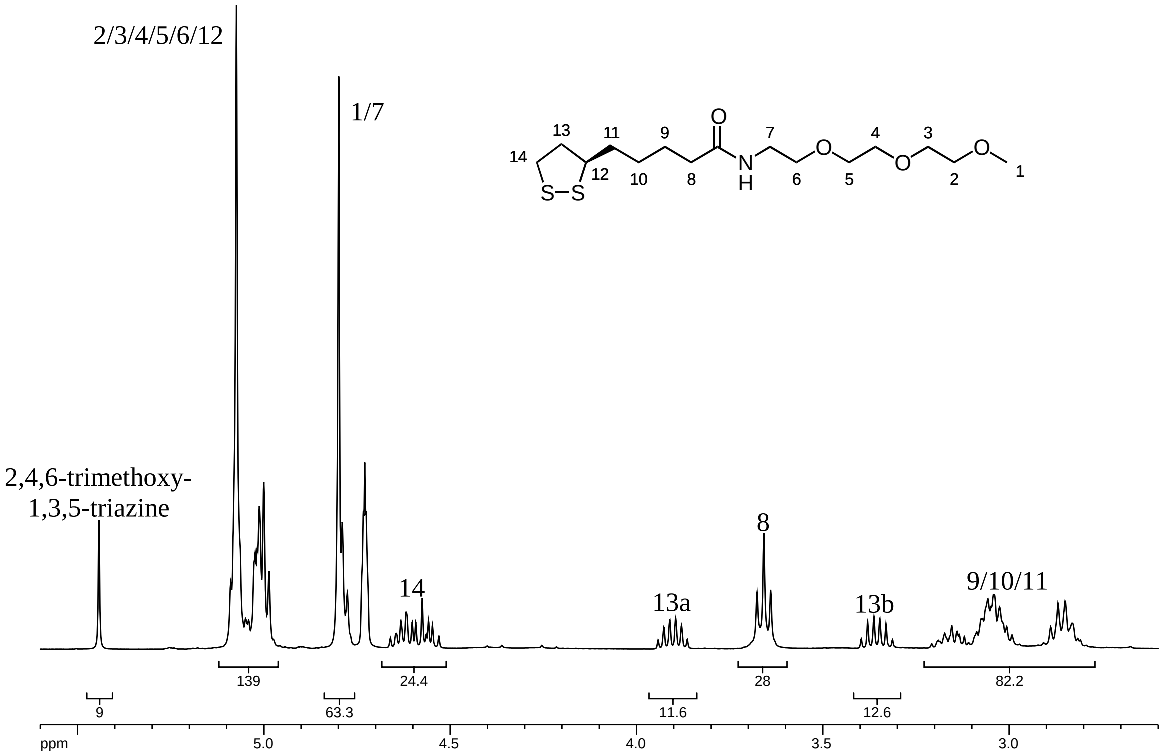

In this spectrum, the signal of the internal standard is marked as are prominent ligand signals. The signal of protons 2/3/4/5/6/12 was used to determine the ligand ratio. The corresponding integral amounted to 139 and the number of absorbing protons 11. Accordingly, the ratio of internal standard and ligand was 1:1.40 and the molar amount of ligand 1.40 µmol. Since 6.34 mg of NP$^{rac\text{-}\mathbf{1}}$ were used, the sample contained 5.85 mg of gold and 0.49 mg of ligand. Of the total amount of nanoparticle, 7.8 wt % were thus ligand molecules. Table S1 summarizes the results obtained for NP$^{rac\text{-}\mathbf{1}}$ and NP$^{R\text{-}\mathbf{1}}$ in different measurements.

Table S1: Compositions of nanoparticles NP$^{rac\text{-}\mathbf{1}}$ and NP$^{R\text{-}\mathbf{1}}$ obtained in different measurements by iodine decomposition.

entry	nanoparticle	total amount / mg	∫ ligand signals / proton	molar amount of ligand / mol	m(ligand) / mg	m(Au) / mg	weight% ligand
1	NP$^{rac\text{-}\mathbf{1}}$	1.83	2.45	2.75×10^{-7}	0.096	1.73	5.3
2		4.40	2.15	2.39×10^{-7}	0.084	4.32	1.9
3		0.69	1.66	1.85×10^{-7}	0.065	0.63	9.4
4		2.71	5.25	5.81×10^{-7}	0.204	2.51	7.5
5		2.41	1.45	1.60×10^{-7}	0.056	2.35	2.3
						Average:	5.3 ± 2.9
6	NP$^{R\text{-}\mathbf{1}}$	4.03	8.01	8.90×10^{-7}	0.313	3.72	7.8
7		6.34	12.64	14.00×10^{-7}	0.494	5.85	7.8
8		1.96	1.65	1.84×10^{-7}	0.065	1.89	3.3
9		2.12	2.05	2.28×10^{-7}	0.080	2.04	3.8
10		0.88	1.40	1.56×10^{-7}	0.055	0.82	6.2
						Average:	5.8 ± 1.9

The results were furthermore used to determine the nanoparticle compositions. These calculations were performed by considering the average nanoparticle diameter of 9.1 nm determined by TEM and assuming spherical shapes. The AuNPs thus had a volume of 395 nm^3 and a surface area of 260 nm^2. With the density of gold of 19.32 g/cm^3, an average weight of 7.62×10^{-9} ng and a number of gold atoms of 23306 per AuNP resulted. These estimates allowed determining the number of ligands per AuNP by using the results in Table S1. First, the determined amounts of gold and ligand were used to calculate the corresponding numbers of nanoparticles and ligand molecules. The ratio of these numbers then yielded the number of ligands per nanoparticle. The results of the corresponding calculations are summarized in Table S2. Accordingly, NP$^{rac\text{-}\mathbf{1}}$ and NP$^{R\text{-}\mathbf{1}}$ contained on average 743 ± 426 and 806 ± 280 ligand molecules, respectively.

Table S2: Compositions of nanoparticles NP$^{rac\text{-}\mathbf{1}}$ and NP$^{R\text{-}\mathbf{1}}$ obtained in different measurements by iodine decomposition.

entry	nanoparticle	# AuNPs	# ligands	# ligands / # AuNPs	nm^2 / ligand	# ligands / nm^2
1	NP$^{rac\text{-}\mathbf{1}}$	2.27×10^{14}	1.65×10^{17}	728	0.36	2.80
2		5.66×10^{14}	1.44×10^{17}	254	1.02	0.98
3		0.82×10^{14}	1.11×10^{17}	1358	0.19	5.22
4		3.29×10^{14}	3.50×10^{17}	1064	0.24	4.09
5		3.09×10^{14}	0.96×10^{17}	311	0.84	1.20
			Average:	743 ± 426	0.53 ± 0.34	2.86 ± 1.64
6	NP$^{R\text{-}\mathbf{1}}$	4.88×10^{14}	5.36×10^{17}	1099	0.24	4.22
7		7.67×10^{14}	8.46×10^{17}	1102	0.24	4.24
8		2.48×10^{14}	1.11×10^{17}	446	0.58	1.72
9		2.68×10^{14}	1.37×10^{17}	513	0.51	1.97
10		1.08×10^{14}	0.94×10^{17}	868	0.30	3.34
			Average:	806 ± 280	0.37 ± 0.14	3.10 ± 1.08

Table S2 also contains the calculated footprint sizes of the ligands and number of ligands per nm, which were calculated by using the number of ligands per AuNP and the nanoparticle surface of 260 nm^2. The means of these values amount to 0.53 ± 0.34 nm^2/ligand and 2.86 ± 1.64 ligands/nm^2 for NP$^{rac\text{-}\mathbf{1}}$ and 0.37 ± 0.14 nm^2/ligand and 3.10 ± 1.08 ligands/nm^2 for NP$^{R\text{-}\mathbf{1}}$.

Evaluation of nanoparticle stability. Nanoparticle solutions with a concentration of 0.5 mg/mL and salt solutions with a concentration of 0.1 mol/L were prepared in water. As salts, $NaNO_3$, NaCl, NaBr, NaI, Na_2SO_4, $NaHCO_3$, Na_2HAsO_4, Na_2HPO_4, $Na_4P_2O_7$, and $Na_5P_3O_{10}$ were used. Eleven vials were prepared, each containing 100 µL of the nanoparticle stock solution. To one vial, water (100 µL) was added while all other vials were treated with a salt solution (100 µL). Photographs of the vials were taken after 30 minutes. The images obtained are shown in Figure 2 of the main article.

Synthesis of the mixed monolayer-protected gold nanoparticles. Aqueous citric acid (10 mL, 0.1 mol/L) was added to a dialyzed stock solution of NP^{cit} (40 mL) to adjust the pH to 3. A ligand mixture was prepared by mixing stock solutions of *R*-**1** and **2** in methanol (0.1 mol/L) and adding citric acid (2 mL, 0.1 mol/L). The amounts of the stock solutions used for the preparation of NP^4,

NP^{10}, NP^{25}, and NP^{35} are specified in Table S3. The ligand solution was added to the reaction mixture, which was then stirred at room temperature for 16 h. The solvent was removed in vacuo and the residue was redissolved in water/methanol 1:2 (v/v, 1.00 mL) and acidified with 0.1 mol/L HNO_3 to pH 3. The nanoparticles were purified by size exclusion chromatography (Sephadex® G-10) by using the same solvent mixture as eluent in which the nanoparticles were dissolved. Subsequently, the obtained nanoparticles were further purified by using centrifugal concentrators. For this, they were dissolved in the same solvent mixture also used for the size exclusion chromatography. The solution was subjected to concentrators with a MWCO membrane (5000 Da) and the solvent removed by centrifugation (3000 rpm, 12 °C). This step was repeated three times. The resulting AuNPs were collected and dried in vacuo. Selected analytical results for each type of nanoparticle thus prepared are collected in Figure S3 and Figure S4. These figures also provide information about the determination of the surface-bound ligand ratio from the corresponding ^{1}H NMR spectra.

Table S3: Amounts of stock solutions of ligands *R*-**1** and **2** in methanol (0.1 mol/L) used for the preparation of NP^{4}, NP^{10}, NP^{25}, and NP^{35}.

	V(*R*-**1**) / mL	*V*(**2**) / mL	*V*(*R*-**1**)/*V*(**2**) during reaction	*V*(*R*-**1**)/*V*(**2**) in product	Yield / mg
NP^{4}	1.80	0.20	90:10	96:4	3.1
NP^{10}	1.60	0.40	80:20	90:10	5.7
NP^{25}	1.20	0.80	60:40	75:25	7.0
NP^{35}	1.00	1.00	50:50	65:35	5.5

(a)

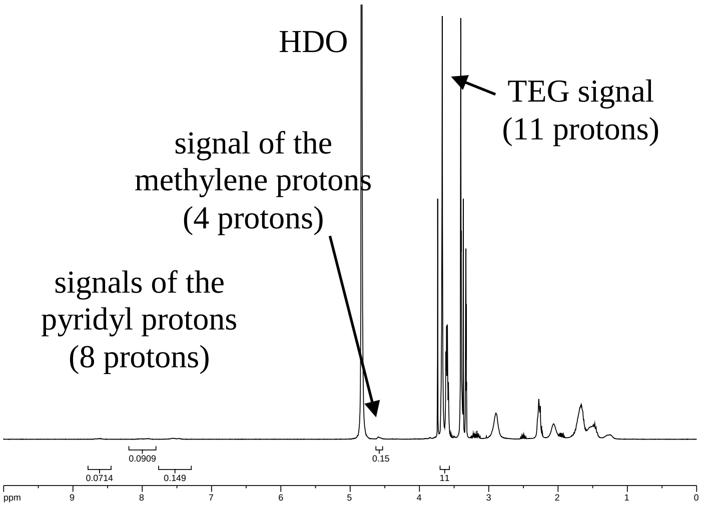

(b)

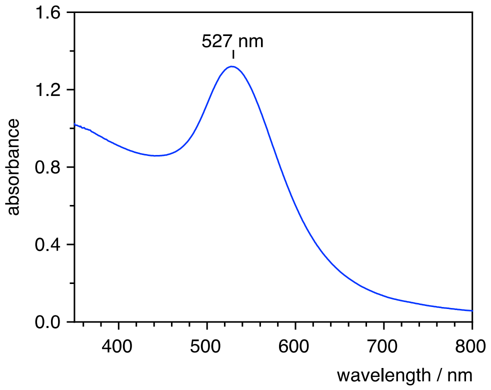

relative content of the functionalized ligand:

$$Content\ (\%) = \frac{0.461/12}{(0.461/12 + 11/11)} \times 100 = 3.7\%$$

(c)

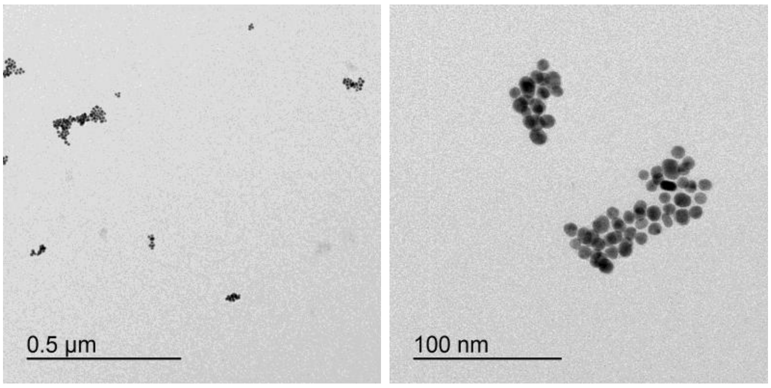

average diameter: 9.2 ± 1.7 nm

(d)

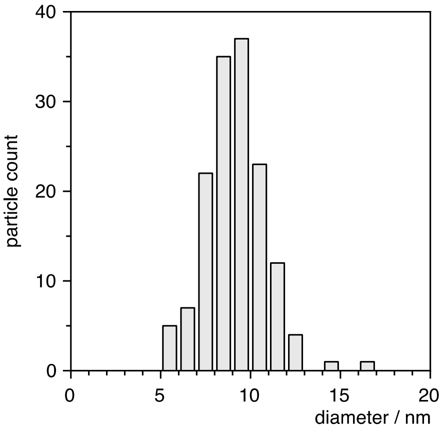

Figure S3: ^{1}H NMR spectrum in D_2O/CD_3OD 1:2 (v/v) (a) and UV–vis spectrum (b) of NP^4 in water/methanol 1:2 (v/v), and a TEM image (c) together with the histogram illustrating the size distribution of the nanoparticles derived from the TEM image (d).

(a)

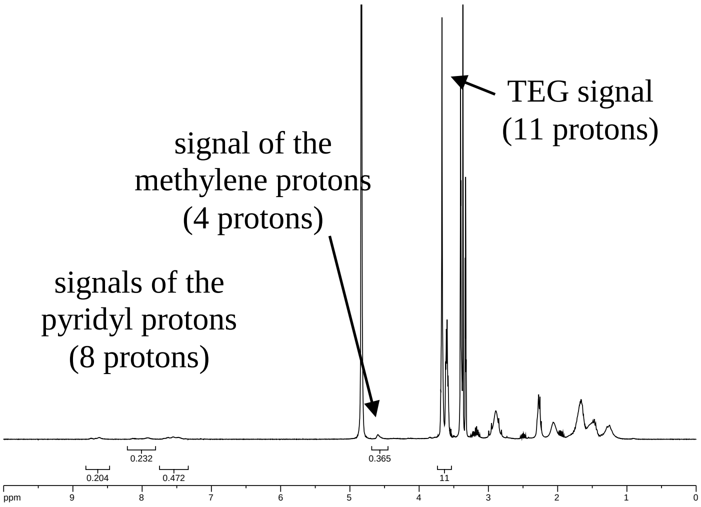

(b)

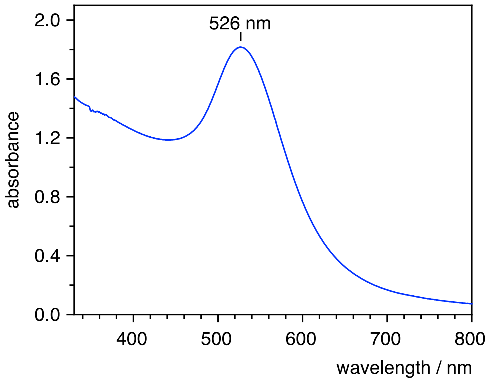

relative content of the functionalized ligand:

$$Content\ (\%) = \frac{1.273/12}{(1.273/12 + 11/11)} \times 100 = 9.6\%$$

(c)

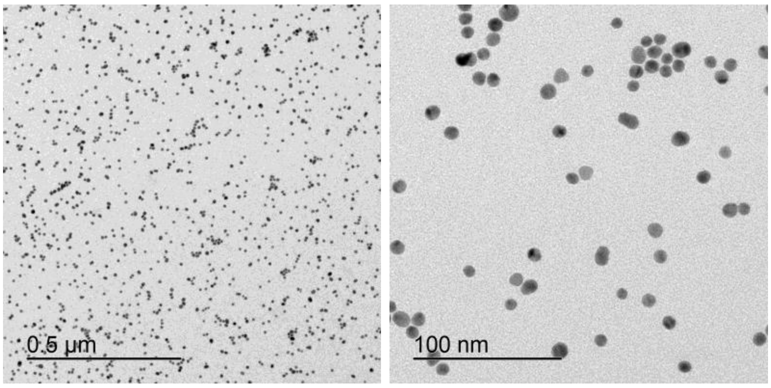

(d)

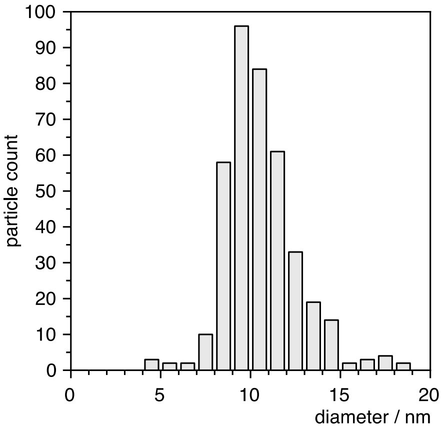

average diameter: 10.5 ± 2.1 nm

Figure S4: ^{1}H NMR spectrum in D_2O/CD_3OD 1:2 (v/v) (a) and UV–vis spectrum (b) of NP^{10} in water/methanol 1:2 (v/v), and a TEM image (c) together with the histogram illustrating the size distribution of the nanoparticles derived from the TEM image (d).

(a)

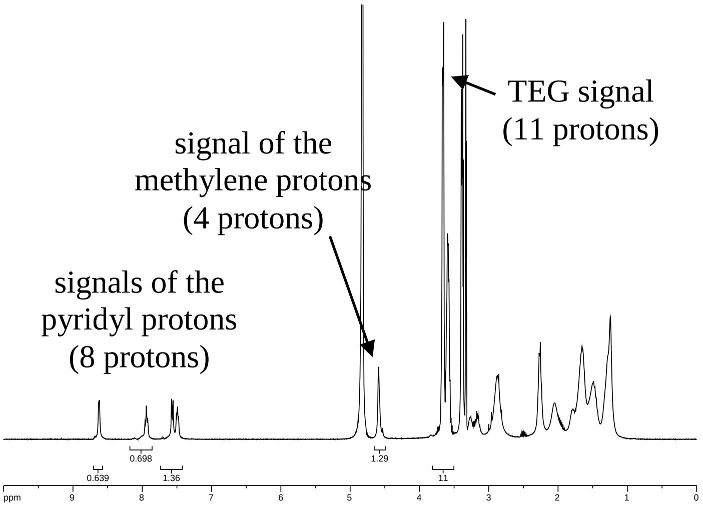

(b)

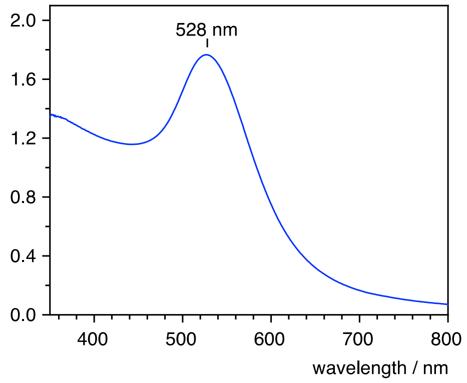

relative content of the functionalized ligand:

$$Content\ (\%) = \frac{3.987/12}{(3.987/12 + 11/11)} \times 100 = 24.9\%$$

(c)

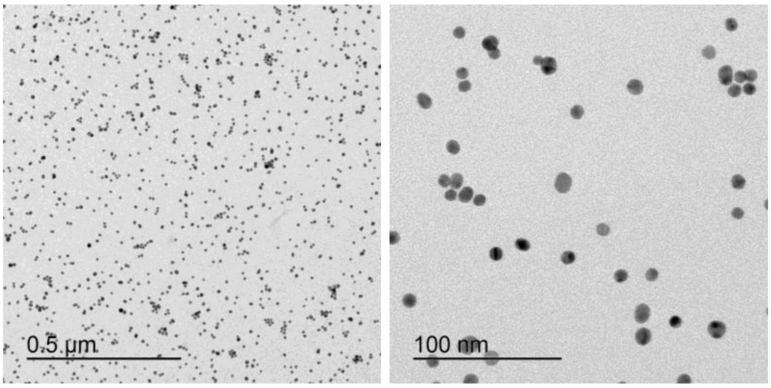

(d)

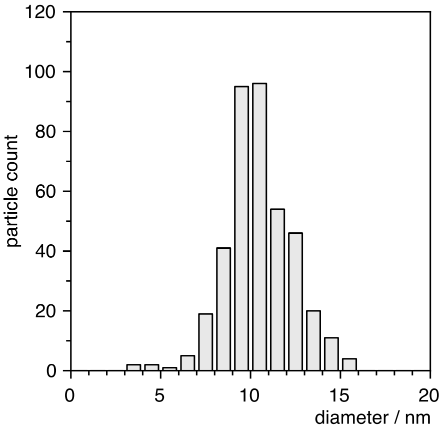

average diameter: 10.3 ± 1.7 nm

Figure S5: ^{1}H NMR spectrum in D_2O/CD_3OD 1:2 (v/v) (a) and UV–vis spectrum (b) of NP[25] in water/methanol 1:2 (v/v), and a TEM image (c) together with the histogram illustrating the size distribution of the nanoparticles derived from the TEM image (d).

(a)

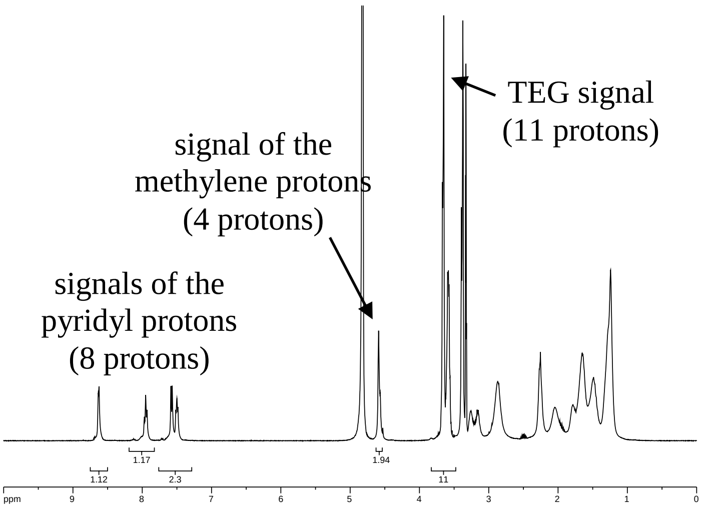

(b)

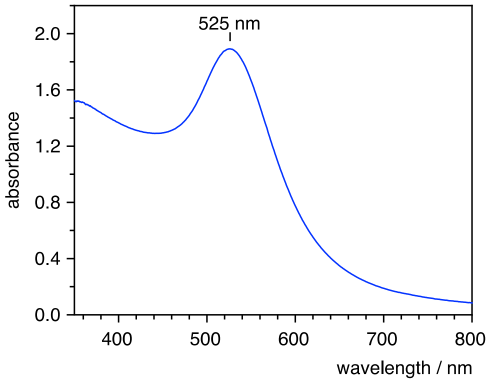

relative content of the functionalized ligand:

$$\mathit{Content}\ (\%) = \frac{6.530/12}{(6.530/12 + 11/11)} \times 100 = 35.2\%$$

(c)

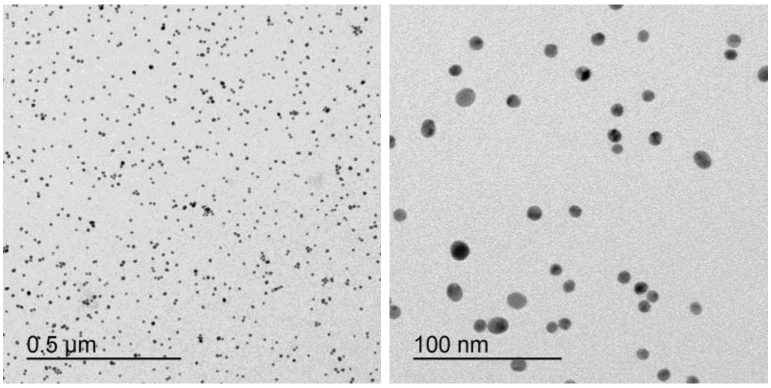

(d)

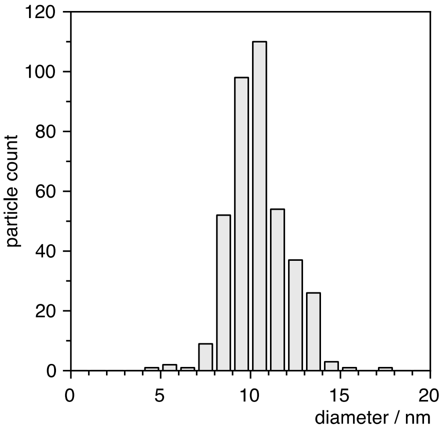

average diameter: 10.3 ± 1.7 nm

Figure S6: ^{1}H NMR spectrum in D_2O/CD_3OD 1:2 (v/v) (a) and UV–vis spectrum (b) of NP[35] in water/methanol 1:2 (v/v), and a TEM image (c) together with the histogram illustrating the size distribution of the nanoparticles derived from the TEM image (d).

Zinc complexation studies. NP^{25} (7.0 mg) was dissolved in D_2O/CD_3OD 1:2 (v/v) (600 µL). To this solution, a solution of $Zn(NO_3)_2$ (0.1 mol/L) in D_2O/CD_3OD 1:2 (v/v) was progressively added as indicated in Table S4. After each addition, the sample was shaken and the ^{1}H NMR spectrum was recorded after 30 min. The concentration of ligand **2** in these solutions was estimated by assuming that NP^{25} contained 5.5 wt % of ligands (average of the contents estimated for NP$^{rac\text{-}\mathbf{1}}$ and NP$^{R\text{-}\mathbf{1}}$, Table S1). The solution thus contained 0.055 × 7.0 mg = 0.385 mg of a mixture of ligands *R*-**1** and **2** in a 75:25 ratio. To determine the molar amount of **2**, a weighted molecular weight of the ligands was calculated by using their relative amounts and corresponding molecular weight: 0.25 × 570.93 + 0.75 × 351.53 = 406.38. The molar amount of **2** in the solution thus amounted to 0.25 × (0.385 mg/406.38 mg/mmol) = 0.24 µmol and the corresponding concentration 3.95×10^{-4} mol/L. The obtained ^{1}H NMR spectra are shown in Figure 3 of the main article.

Table S4: Amounts of the $Zn(NO_3)_2$ stock solution (0.1 mol/L) added to the solution of NP^{25} in D_2O/CD_3OD 1:2 (v/v) and estimated concentrations of **2** and zinc(II) ions in the solutions resulting after each addition.

entry	*V*(Zn(NO$_3$)$_2$) / µl	*V*(total) / µl	*M*(Zn^{2+}) / µmol	*M*(**2**) / µmol	*M*(Zn^{2+}) / *M*(**2**)
1	0.0	600.0	0.00	0.24	0
2	2.1	602.1	0.21	0.24	1
3	19.3	621.4	2.14	0.24	9
4	10.7	632.1	3.21	0.24	14
5	10.7	642.8	4.28	0.24	18
6	10.7	653.5	5.35	0.24	23
7	10.7	664.2	6.42	0.24	27
8	10.7	674.9	7.49	0.24	32
9	10.7	685.6	8.56	0.24	36
10	10.7	696.3	9.63	0.24	41
11	10.7	707.0	10.70	0.24	45
12	11.0	718.0	11.80	0.24	50
13	10.0	728.0	12.80	0.24	54

For the preparation of the nanoparticles $NP^{4\text{-}Zn}$, $NP^{10\text{-}Zn}$, $NP^{25\text{-}Zn}$, and $NP^{35\text{-}Zn}$, a known amount of the respective precursor was dissolved in D_2O/CD_3OD 1:2 (v/v) (600 µL). A solution of $Zn(NO_3)_2$ (0.1 mol/L) in D_2O/CD_3OD 1:2 (v/v) was added such that the resulting mixture contained 50 equiv of $Zn(NO_3)_2$ per DPA moiety. The number of DPA moieties was estimated from the weights of the nanoparticles by using the above approach. The exact conditions for selected experiments are summarized in Table S5. An ^{1}H NMR spectrum was recorded after 30 min to confirm that complexation was complete. Afterward, the solutions were evaporated, the residues dried and used in the subsequent binding studies. The last column in Table S5 specifies the total concentration of Zn^{2+} (of which 2% were bound to the DPA moieties) in a nanoparticle solution with a concentration of 0.25 mg/mL as used in the binding studies.

Table S5: Conditions of the preparation of nanoparticles $NP^{4\text{-}Zn}$, $NP^{10\text{-}Zn}$, $NP^{25\text{-}Zn}$, and $NP^{35\text{-}Zn}$ from the respective metal-free precursors.

AuNP	*m*(AuNP) / mg	*m*(ligands) / mg	weighted M.W. of ligands / g mol^{-1}	*M*(**2**) / µmol	*M*($Zn(NO_3)_2$) / µmol	*V*($Zn(NO_3)_2$) stock solution / µl	*c*(Zn^{2+}) in binding studies / µmol/L
$NP^{4\text{-}Zn}$	3.1	0.171	360.3	0.02	0.95	9.5	72
$NP^{10\text{-}Zn}$	5.7	0.314	373.5	0.08	4.20	42.0	162
$NP^{25\text{-}Zn}$	7.0	0.385	406.4	0.24	11.84	118.4	320
$NP^{35\text{-}Zn}$	5.5	0.303	428.3	0.25	12.36	123.6	394

Binding studies

Visual binding study. All experiments were performed by using HPLC grade water and methanol. The nanoparticles and salts were weighed by using an analytical precision balance. The nanoparticles were dissolved in water/methanol 1:2 (v/v) and the sodium salts in water to obtain solutions containing 0.25 mg/mL of nanoparticles ($NP^{R\text{-}\mathbf{1}}$, $NP^{4\text{-}Zn}$, $NP^{10\text{-}Zn}$, $NP^{25\text{-}Zn}$, and $NP^{35\text{-}Zn}$) and salt solutions with a concentration of 10 mmol/L. As salts, $NaNO_3$, NaCl, NaBr, NaI, Na_2SO_4, $NaHCO_3$, Na_2HAsO_4, Na_2HPO_4, $Na_4P_2O_7$, and $Na_5P_3O_{10}$ were used. Eleven vials were prepared, each containing 200 µL of the nanoparticle stock solution. One vial was used as blank while each of the other vials was treated with a salt solution. Initially, 2 µL of the salt solutions were added, followed by two further additions of 2 µL and a final addition of 4 µL to afford salt concentrations after each addition of 99, 196, 291, and 476 µmol/L. Photographs of the vials were taken 5 min after each addition. This experiment was repeated at least three times. The results of all experiments are shown in Figure S7–Figure S10.

Competition experiment. A stock solution of $NP^{10\text{-}Zn}$ (0.25 mg/mL) was prepared in water/methanol 1:2 (v/v) and stock solutions of NaCl (0.1 mol/L), $NaNO_3$ (0.1 mol/L), Na_2SO_4 (0.1 mol/L), and $Na_4P_2O_7$ (10 mmol/L) were prepared in HPLC grade water. Three vials were set up, each containing the $NP^{10\text{-}Zn}$ stock solution (200 µL). No salt solutions were added to the first vial. The NaCl, $NaNO_3$, and Na_2SO_4 stock solutions (3 µL each) were added to the two other vials. To the third vial, the $Na_4P_2O_7$ stock solution (3 µL) was additionally added. All vials were made up to total volumes of 212 µL with water/methanol 1:2 (v/v). A photograph of the vials, taken after 5 min, is shown in Figure 5 of the main article.

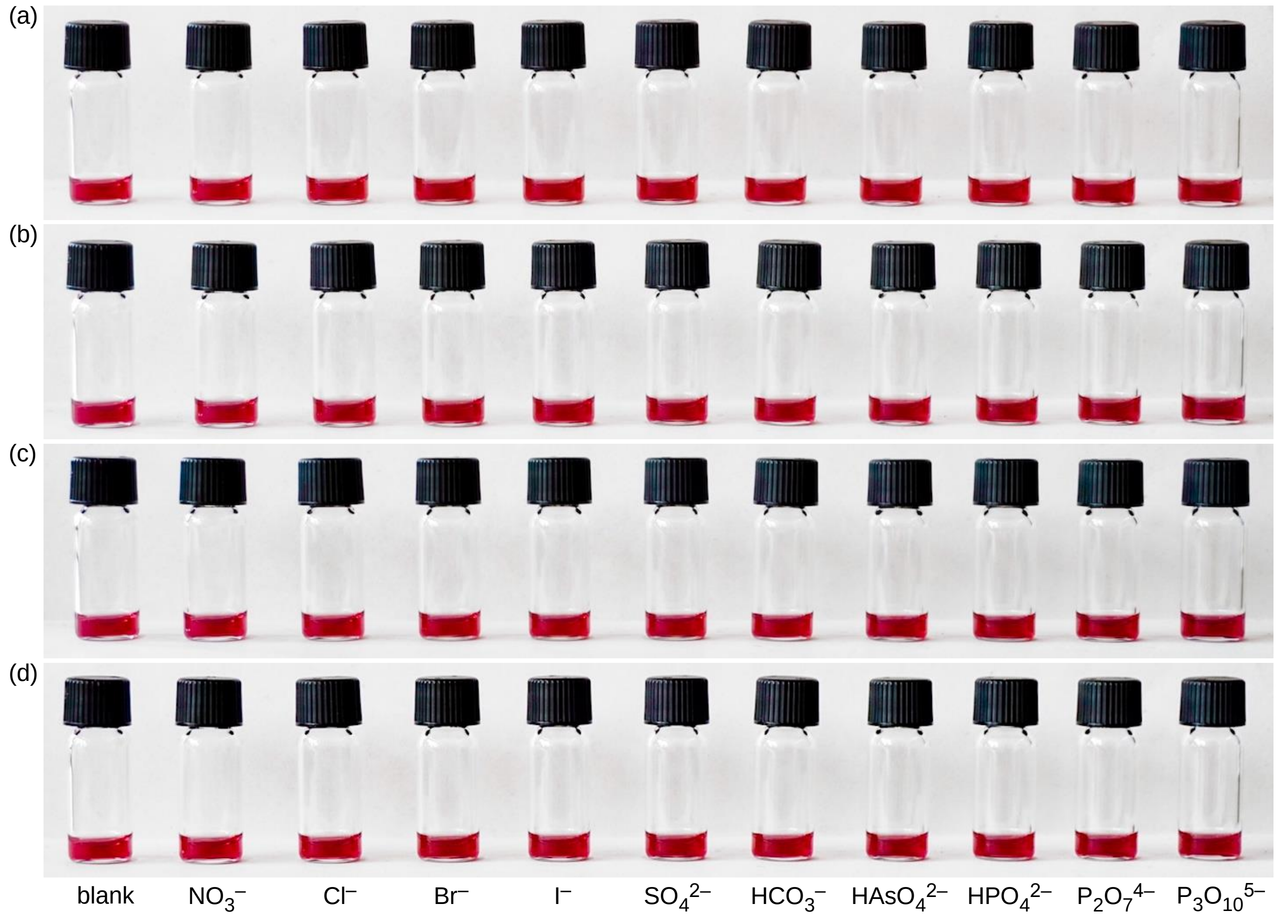

Figure S7: Images of vials containing solutions of $NP^{R\text{-}1}$ (0.25 mg/mL) in water/methanol 1:2 (v/v) containing additional sodium salts of the anions specified in the bottom row at concentrations of 99 µmol/L (a), 196 µmol/L (b), 291 µmol/L (c), and 476 µmol/L (d). The photos were taken 5 min after the salt additions.

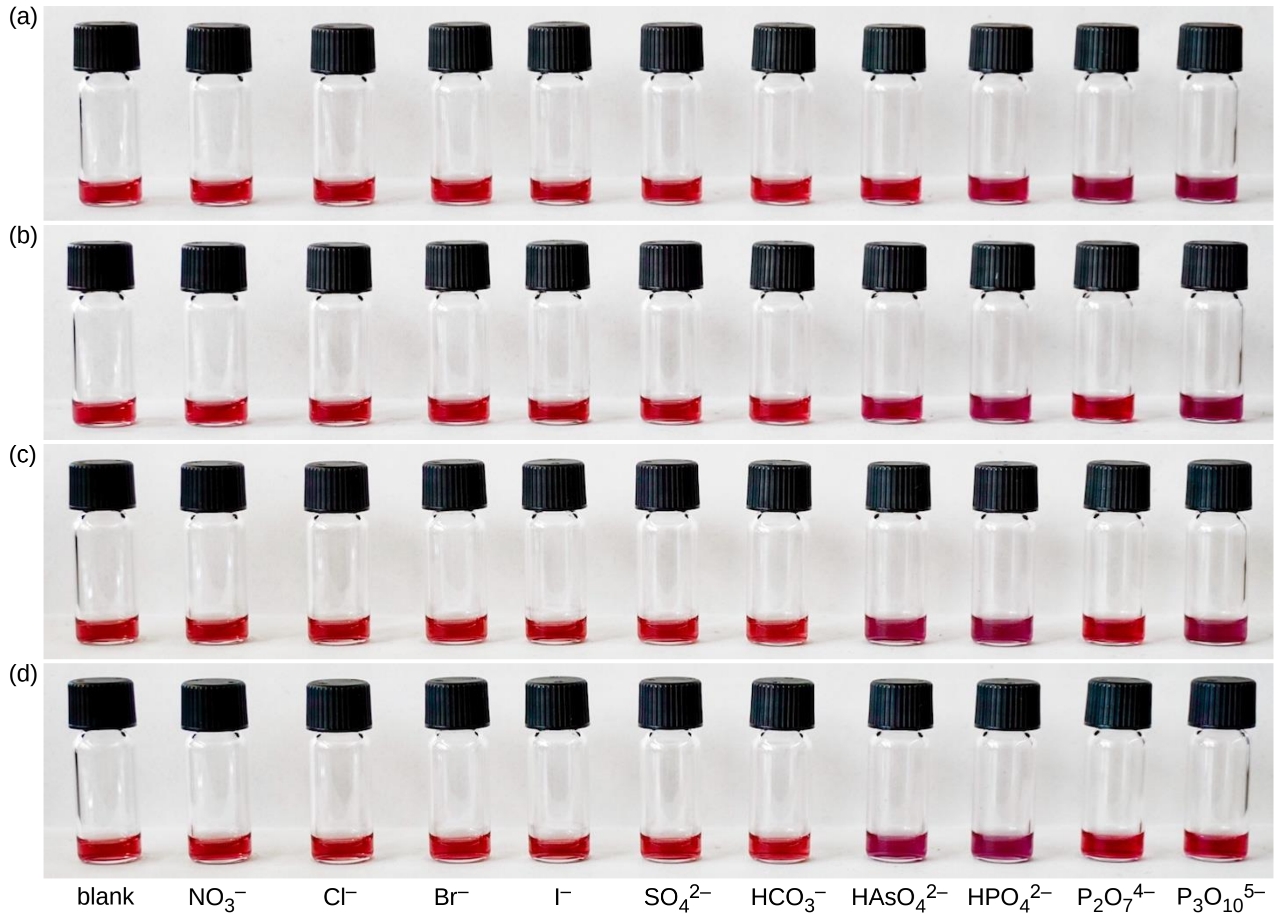

Figure S8: Images of vials containing solutions of $NP^{4\text{-}Zn}$ (0.25 mg/mL) in water/methanol 1:2 (v/v) containing additional sodium salts of the anions specified in the bottom row at concentrations of 99 µmol/L (a), 196 µmol/L (b), 291 µmol/L (c), and 476 µmol/L (d). The photos were taken 5 min after the salt additions.

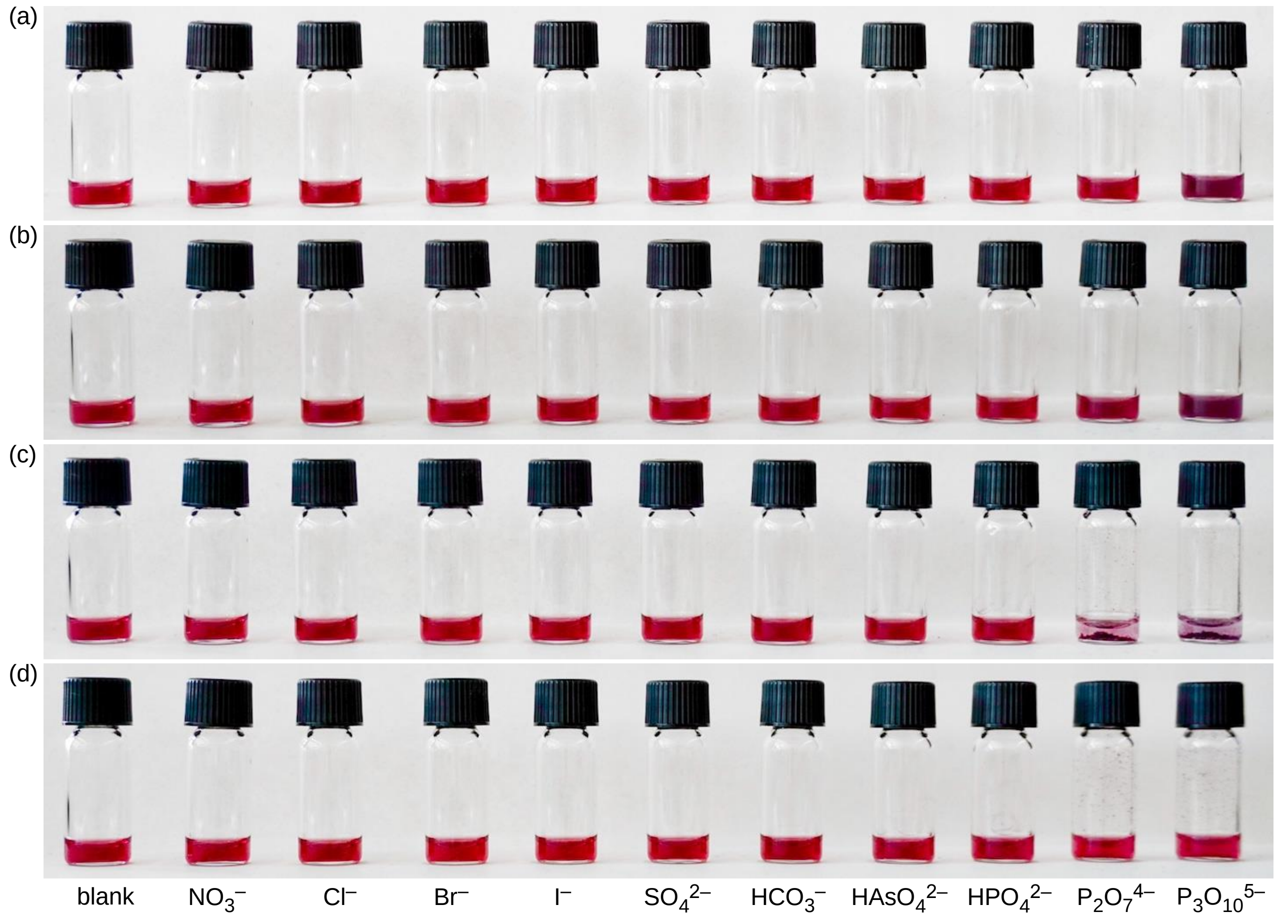

Figure S9: Images of vials containing solutions of $NP^{25\text{-}Zn}$ (0.25 mg/mL) in water/methanol 1:2 (v/v) containing additional sodium salts of the anions specified in the bottom row at concentrations of 99 µmol/L (a), 196 µmol/L (b), 291 µmol/L (c), and 476 µmol/L (d). The photos were taken 5 min after the salt additions.

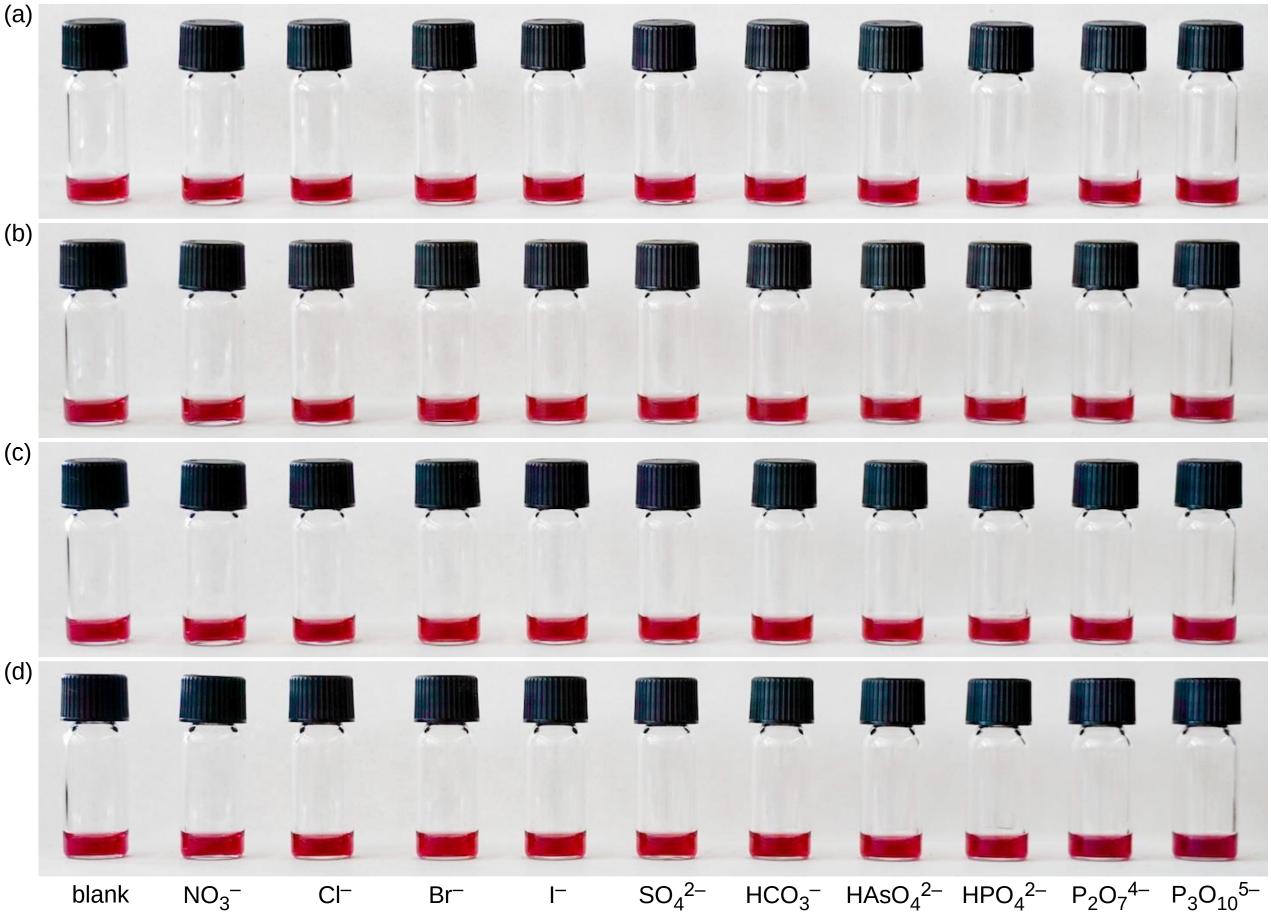

Figure S10: Images of vials containing solutions of $NP^{35\text{-}Zn}$ (0.25 mg/mL) in water/methanol 1:2 (v/v) containing additional sodium salts of the anions specified in the bottom row at concentrations of 99 µmol/L (a), 196 µmol/L (b), 291 µmol/L (c), and 476 µmol/L (d). The photos were taken 5 min after the salt additions.

Effect of insoluble zinc phosphates on the nanoparticles. Stock solutions of $NP^{R\text{-}\mathbf{1}}$ (0.5 mg/mL) and $Zn(NO_3)_2$ (0.1 mol/L) were prepared in water/methanol 2:1 (v/v). A stock solution of $Na_4P_2O_7$ (0.1 mol/L) was prepared in HPLC grade water. Two vials were prepared, each containing the $NP^{R\text{-}\mathbf{1}}$ stock solution (100 µL) and additional water/methanol 1:2 (v/v, 100 µL). No salt solutions were added to the first vial. To the second vial, the $Zn(NO_3)_2$ stock solution (20 µL) and the $Na_4P_2O_7$ stock solution (20 µL) were added. The first vial was made up to 240 µL with water/methanol 1:2 (v/v). The nanoparticles in both solutions remained in solution, rendering the solutions colored red, although in the second vial the precipitation of a white solid was observed (Figure S11).

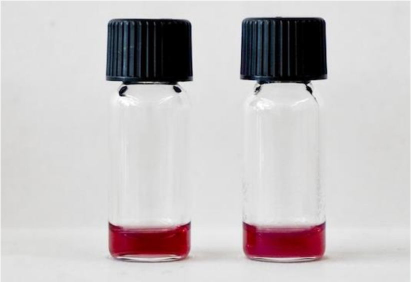

Figure S11: Images of vials containing solutions of $NP^{R\text{-}\mathbf{1}}$ (0.21 mg/mL) in water/methanol 1:2 (v/v). To the right vial $Zn(NO_3)_2$ and $Na_4P_2O_7$ were added to give concentrations of 8.3 mmol/L each. All photos were taken 5 min after preparation of the samples.

UV–vis spectroscopic binding study. The measurements were performed at 22 °C by using semi-micro PMMA disposable cuvettes with a 1 cm path length and HPLC grade water and methanol as solvents. The nanoparticles, Na_2HPO_4, $Na_4P_2O_7$, and $Na_5P_3O_{10}$ were weighed by using an analytical precision balance. Stock solutions of $NP^{4\text{-}Zn}$, $NP^{10\text{-}Zn}$, $NP^{25\text{-}Zn}$, and $NP^{35\text{-}Zn}$ were prepared in water/methanol 1:2 (v/v) containing 0.25 mg/mL of the corresponding nanoparticle. For each salt, three stock solutions with concentrations of 1 mmol/L (salt solution #1), 10 mmol/L (salt solution #2), and 100 mmol/L (salt solution #3) were prepared in HPLC grade water. As blank, water/methanol 1:2 (v/v) was used.

Each series of measurements was performed in one cuvette. The first measurement involved adding the nanoparticle solution (1 mL) to the cuvette and recording the UV-vis spectrum between 350 and 800 nm. For the following measurements, a defined volume of a salt stock solution was added, the cuvette shaken, and the UV–vis spectrum recorded exactly 10 min after the addition. The exact amounts of the salt solutions and the sequence of their addition are summarized in Table S6–Table S9. The obtained spectra are shown in Figure S12–Figure S15. In these figures, the spectrum of the AuNPs in the absence of the salts is shown in blue. The spectrum at the concentration at which a blue shift of the maximum wavelength of the SPR band was first observed is depicted in orange, that at which precipitation started to set in in red, and that at which precipitation was maximum in cyan. In some cases, a maximum effect was observed immediately when precipitation first occurred and, in these cases, only the cyan spectrum is shown. Spectra in shades of green depict solutions in which the precipitate had redissolved at higher salt concentrations.

Table S6: Amounts of salt solutions and sequence of addition to the solution of $NP^{4\text{-}Zn}$ in water/methanol 1:2 (v/v) and concentrations resulting after each addition.

entry	# stock solution	*c*(stock solution) / mmol/L	*V*(salt solution) / µL	*c*(salt) in sample / µmol/L
1	-	-	0	0
2	1	1	1	1
3	1	1	4	5
4	1	1	5	10
5	2	10	1.5	25
6	2	10	2.5	49
7	2	10	2.5	74
8	2	10	2.5	98
9	3	100	1	196
10	3	100	1	294
11	3	100	1	391
12	3	100	1	489
13	3	100	5	973

(a)

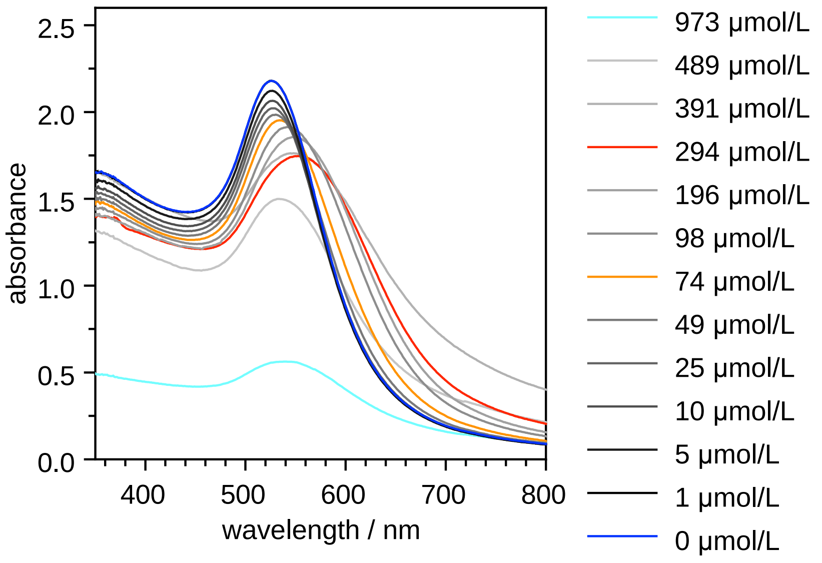

(b)

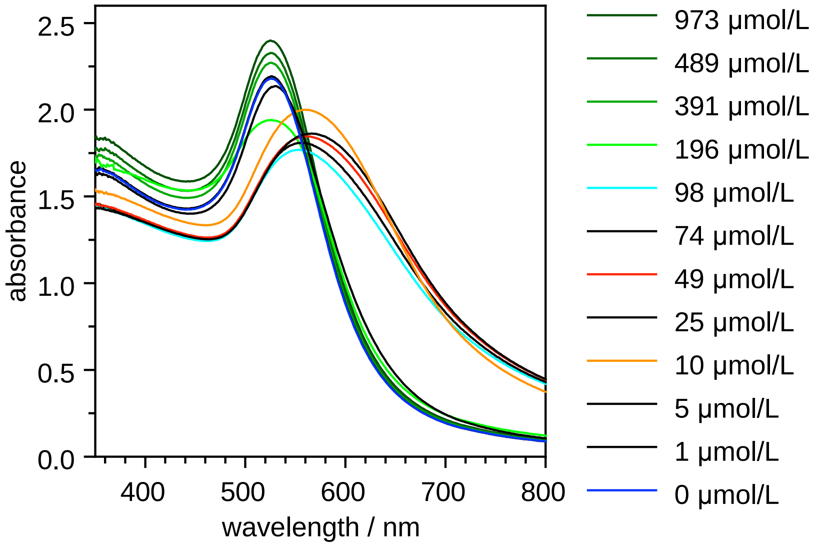

(c)

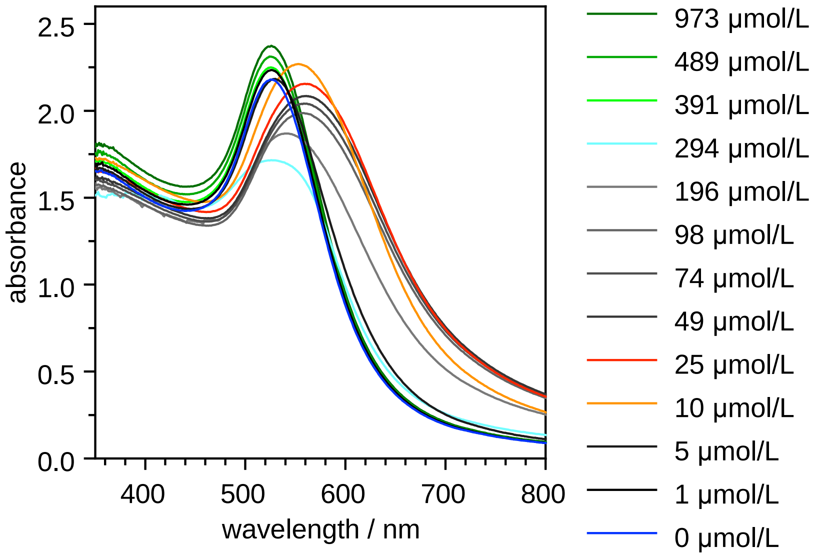

Figure S12: Selected UV–vis spectra of $NP^{4\text{-}Zn}$ (0.25 mg/mL in the initial measurement) in water/methanol 1:2 (v/v) containing between 0 and 973 µmol/L of Na_2HPO_4 (a), $Na_4P_2O_7$, (b), and $Na_5P_3O_{10}$ (c). The spectra were measured 10 min after each addition.

Table S7: Amounts of diphosphate and triphosphate solutions and sequence of addition to the solution of $NP^{10\text{-}Zn}$ in water/methanol 1:2 (v/v) and concentrations resulting after each addition.

entry	# stock solution	*c*(stock solution) / mmol/L	*V*(salt solution) / µL	*c*(salt) in sample / µmol/L
1	-	-	0	0
2	1	1	1	1
3	1	1	4	5
4	1	1	5	10
5	2	10	1.5	25
6	2	10	2.5	49
7	2	10	2.5	74
8	2	10	2.5	98
9	2	10	2.5	122
10	2	10	2.5	146
11	2	10	2.5	170
12	2	10	2.5	194
13	2	10	2.5	218
14	2	10	2.5	242
15	2	10	2.5	265
16	2	10	2.5	289
17	3	100	1	385
18	3	100	1	480
19	3	100	5	956

The HPO_4^{2-} titration was performed as indicated in Table S8 only that after entry 7, 2.5 µL of stock solution #2 was added eight times, followed by 1 µL and then 5 µL of stock solution #3.

(a)

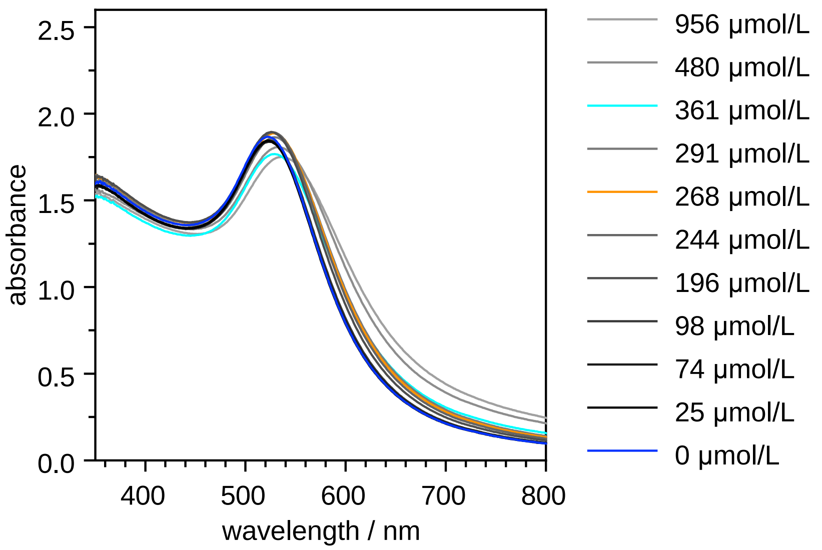

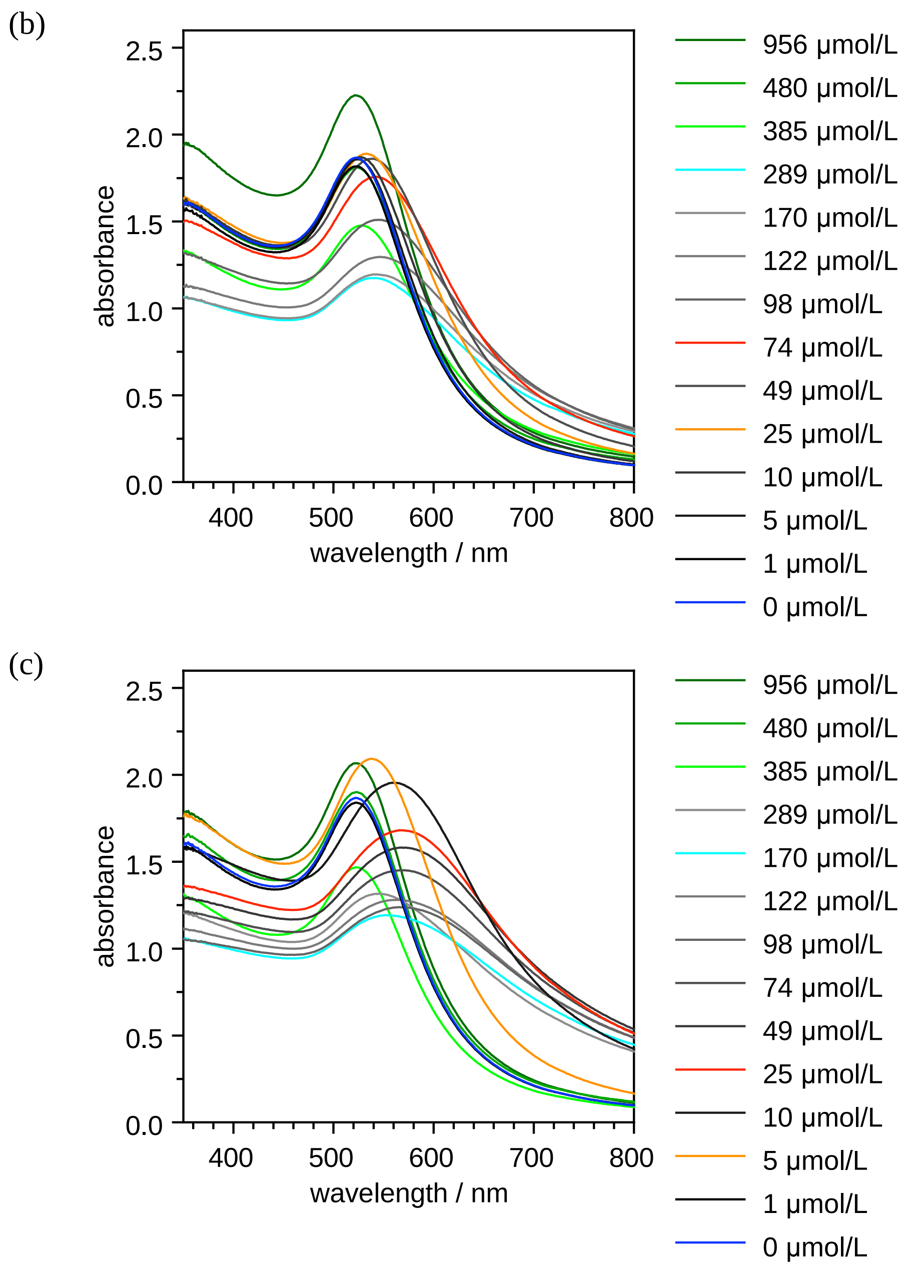

Figure S13: Selected UV–vis spectra of $NP^{10\text{-}Zn}$ (0.25 mg/mL in the initial measurement) in water/methanol 1:2 (v/v) containing between 0 and 956 μmol/L of Na_2HPO_4 (a), $Na_4P_2O_7$, (b), and $Na_5P_3O_{10}$ (c). The spectra were measured 10 min after each addition.

Table S8: Amounts of salt solutions and sequence of addition to the solution of $NP^{25\text{-}Zn}$ in water/methanol 1:2 (v/v) and concentrations resulting after each addition.

entry	# stock solution	*c*(stock solution) / mmol/L	*V*(salt solution) / µL	*c*(salt) in sample / µmol/L
1	-	-	0	0
2	1	1	10	10
3	2	10	1.5	25
4	2	10	2.5	49
5	2	10	2.5	74
6	2	10	2.5	98
7	3	100	1	196
8	2	10	2.5	220
9	2	10	2.5	244
10	2	10	2.5	268
11	2	10	2.5	291
12	3	100	1	388
13	3	100	1	484
14	3	100	5	964

(a)

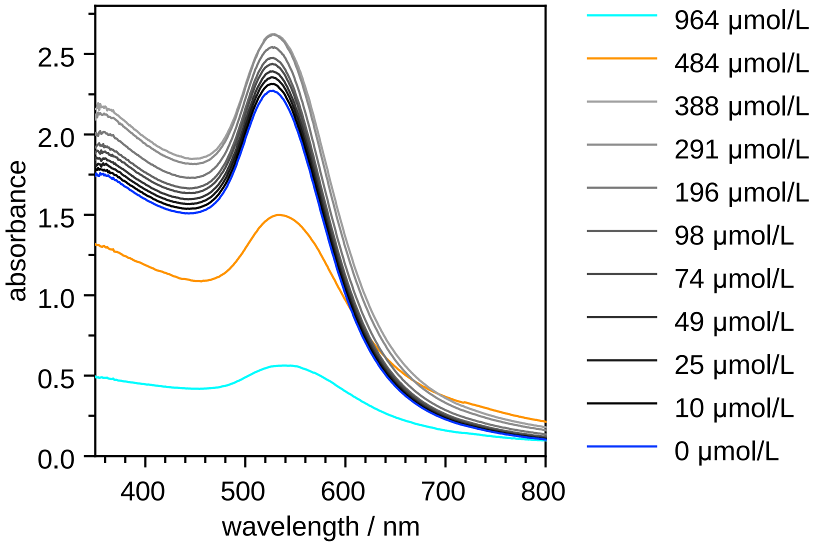

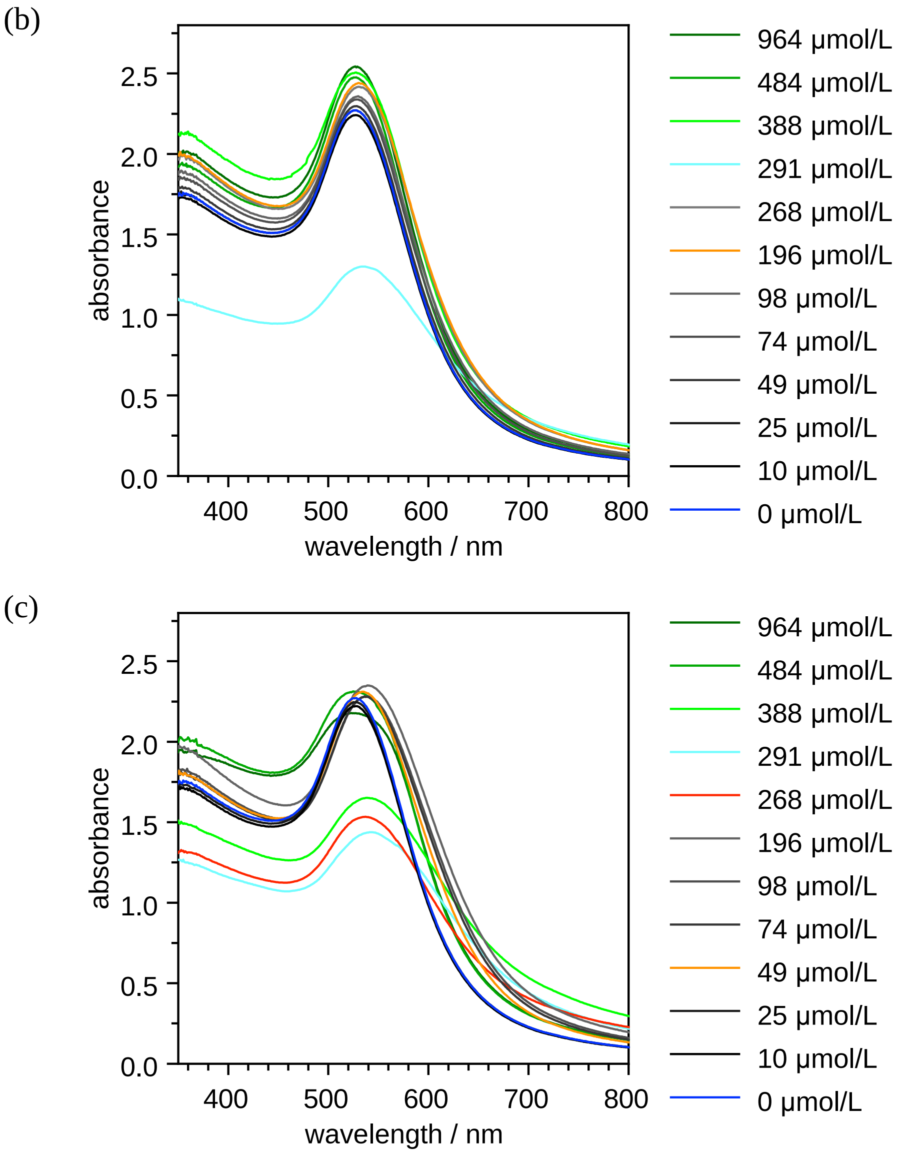

Figure S14: Selected UV–vis spectra of $NP^{25\text{-}Zn}$ (0.25 mg/mL in the initial measurement) in water/methanol 1:2 (v/v) containing between 0 and 964 µmol/L of Na_2HPO_4 (a), $Na_4P_2O_7$, (b), and $Na_5P_3O_{10}$ (c). The spectra were measured 10 min after each addition.

Table S9: Amounts of salt solutions and sequence of addition to the solution of $NP^{35\text{-}Zn}$ in water/methanol 1:2 (v/v) and concentrations resulting after each addition.

entry	# stock solution	*c*(stock solution) / mmol/L	*V*(salt solution) / µL	*c*(salt) in sample / µmol/L
1	-	-	0	0
2	1	1	10	10
3	2	10	1.5	25
4	2	10	2.5	49
5	2	10	2.5	74
6	2	10	2.5	98
7	3	100	1	196
8	3	100	1	294
9	3	100	1	391
10	3	100	1	489
11	3	100	1	586
12	3	100	1	683
13	3	100	1	780
14	3	100	1	876
15	3	100	1	973
16	3	100	5	1452

(a)

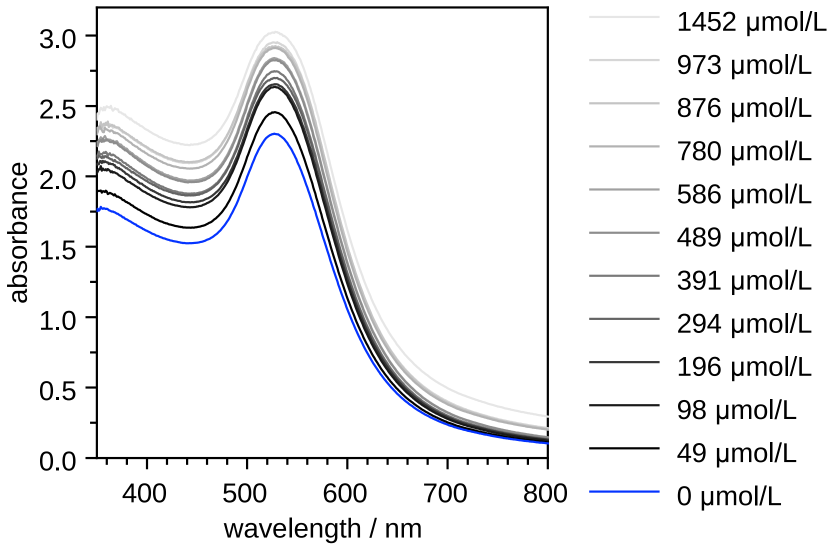

(b)

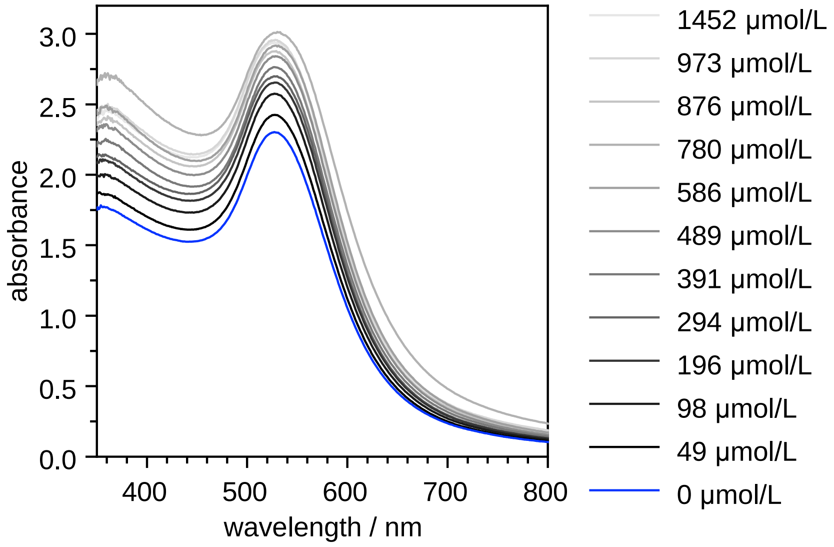

(c)

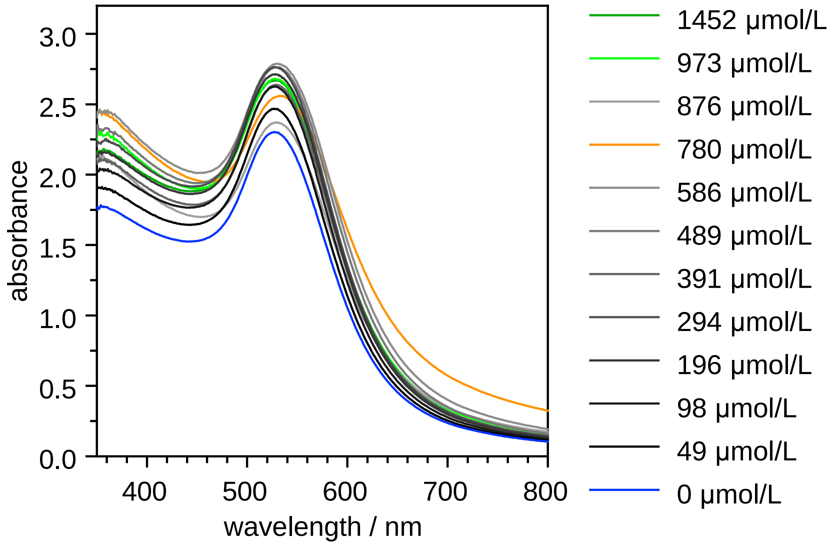

Figure S15: Selected UV–vis spectra of $NP^{35\text{-}Zn}$ (0.25 mg/mL in the initial measurement) in water/methanol 1:2 (v/v) containing between 0 and 1452 µmol/L of Na_2HPO_4 (a), $Na_4P_2O_7$, (b), and $Na_5P_3O_{10}$ (c). The spectra were measured 10 min after each addition.

Transmission electron microscopy. The experiments were performed by using HPLC grade water and methanol. $NP^{10\text{-}Zn}$ and sodium diphosphate were weighed by using an analytical precision balance. The nanoparticles were dissolved in water/methanol 1:2 (v/v) to give a concentration of 0.25 mg/mL and the salt to give a concentration of 10 mmol/L. A TEM image of the nanoparticle stock solution was recorded prior to the salt addition. The stock solution (200 µL) of $NP^{10\text{-}Zn}$ was then treated with the sodium diphosphate solution (3 µL), which caused precipitation of the nanoparticles, and the TEM image was recorded 5 min after the salt addition. Subsequently, additional 7 µL of the salt solution were added and the TEM image recorded after another 5 min.

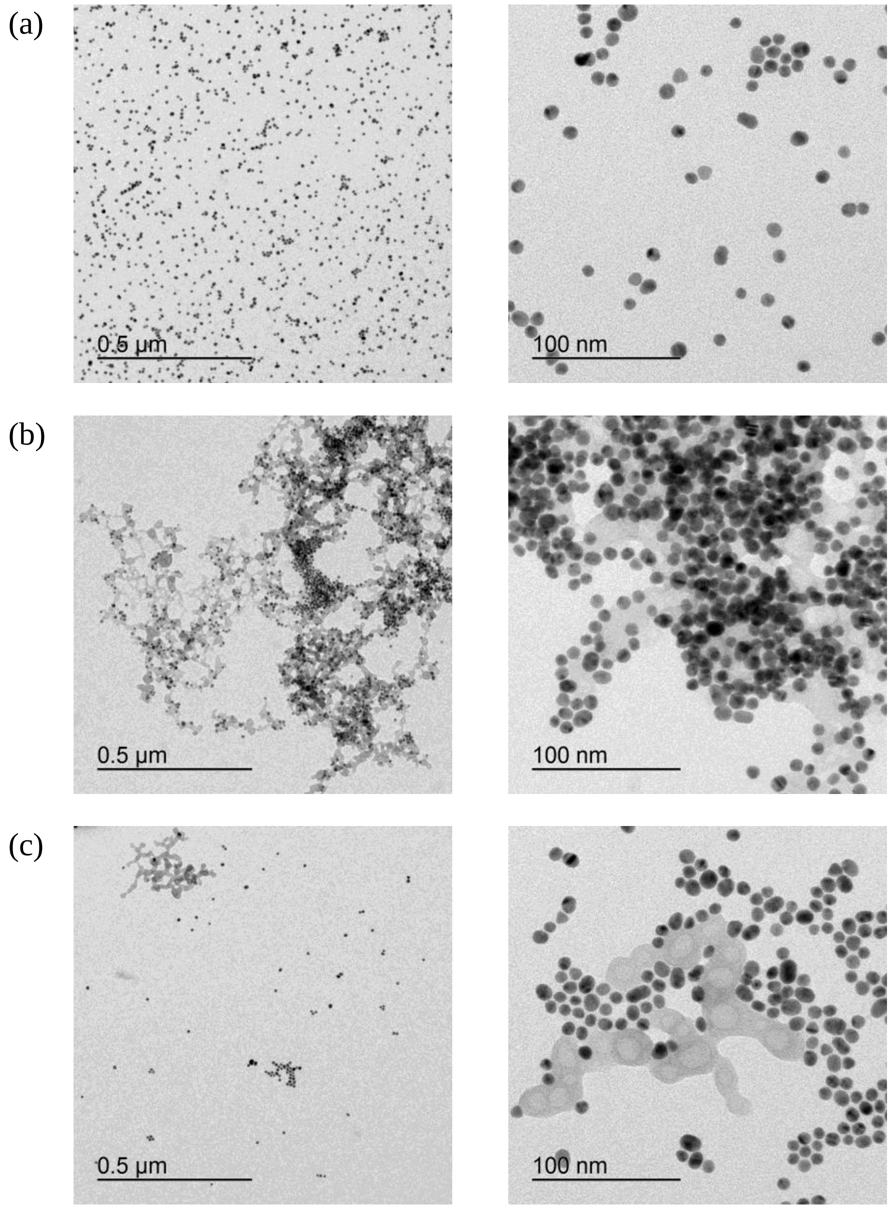

Figure S16: TEM images of $NP^{10\text{-}Zn}$ (0.25 mg/mL) in water/methanol 1:2 (v/v) before (a) and after the addition of $Na_4P_2O_7$ to give a concentration of 148 μmol/L (b). The image in (c) was obtained after increasing the diphosphate concentration to 476 μmol/L.

For the images obtained for $NP^{R\text{-}\mathbf{1}}$, a nanoparticle stock solution was prepared in water/methanol 1:2 (v/v) to give a concentration of 0.25 mg/mL. In addition, a stock solution of $Zn(NO_3)_2$ was prepared in water/methanol 1:2 (v/v) and a stock solution of $N_4P_2O_7$ in water, both having a concentration of 0.1 mol/L. The solution of $NP^{R\text{-}\mathbf{1}}$ (200 μL) was treated with the $Zn(NO_3)_2$ stock solution (2 μL) and a TEM imaged recorded after 5 min. Subsequently, the $N_4P_2O_7$ stock solution was added (2 μL) and another TEM image recorded after 5 min.

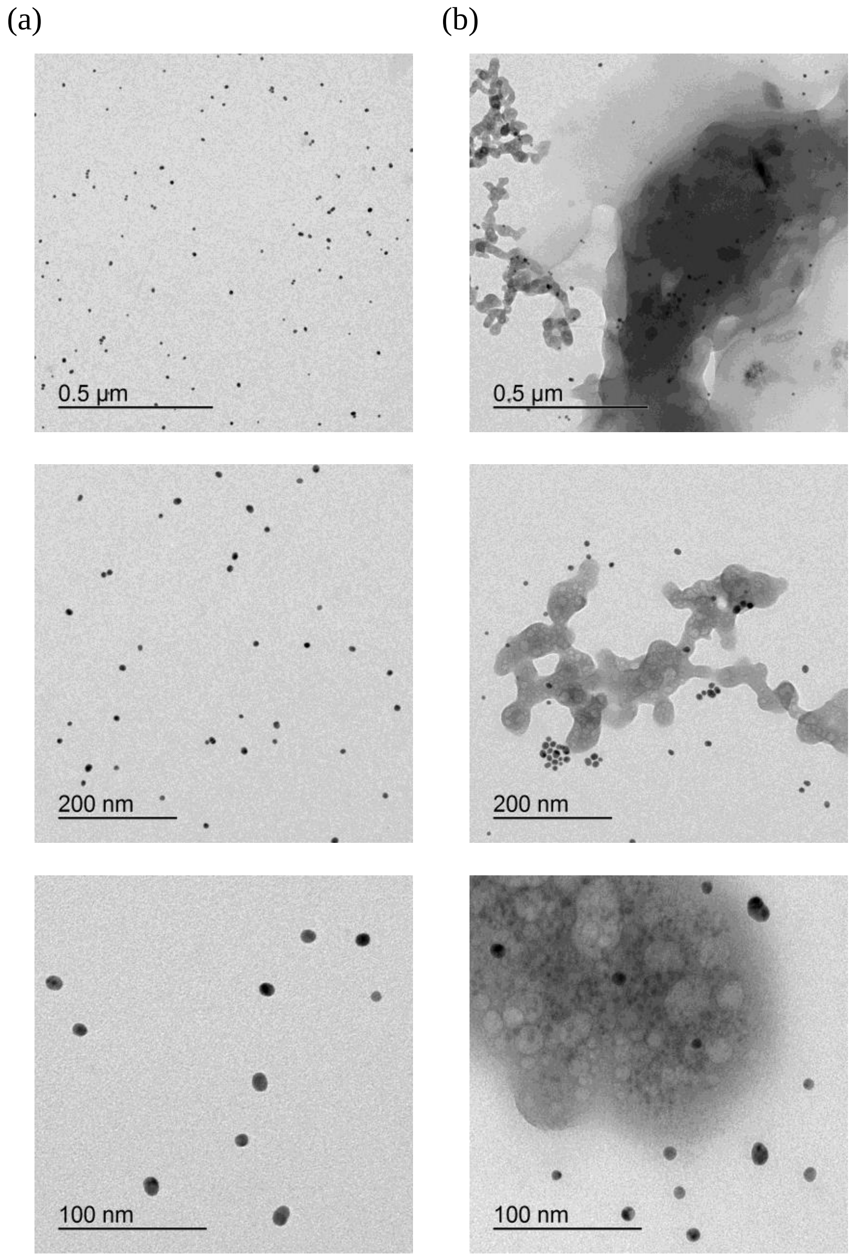

Figure S17: TEM images of $NP^{\textit{R}\text{-}\mathbf{1}}$ (0.25 mg/mL) in water/methanol 1:2 (v/v) in the presence of 0.98 mmol/L of $Zn(NO)_3$ before (a) and after the addition of $Na_4P_2O_7$ to give a concentration of 0.98 mmol/L (b).

References

1. Fiala, T.; Sleziakova, K.; Marsalek, K.; Salvadori, K.; Sindelar V. *J. Org. Chem.* **2018**, *83*, 1903–1912.
2. Incarvito, C.; Lam, M.; Rhatigan, B.; Rheingold, A. L.; Qin, C. J.; Gavrilova, A. L.; Bosnich, B. *J. Chem. Soc., Dalton Trans.* **2001**, *23*, 3478–3488.
3. Pfammatter, M. J.; Siljegovic, V.; Darbre, T.; Keese, K. *Helv. Chim. Acta* **2001**, *84*, 678–689.
4. Ojea-Jiménez, I.; Bastús, N. G.; Puntes, V. J. *Phys. Chem. C* **2011**, *115*, 15752–15757.
